# Automated generation of discharge summaries: leveraging large language models with clinical data

**DOI:** 10.1038/s41598-025-01618-7

**Published:** 2025-05-12

**Authors:** Matthias Ganzinger, Nicola Kunz, Pascal Fuchs, Cornelia K. Lyu, Martin Loos, Martin Dugas, Thomas M. Pausch

**Affiliations:** 1 Institute of Medical Informatics, Heidelberg University, Heidelberg, Germany; 2 Department of General, Visceral, and Transplantation Surgery, Heidelberg University Hospital, Heidelberg, Germany

**Keywords:** Health care, Pancreatic disease

## Abstract

This study explores the use of open-source large language models (LLMs) to automate generation of German discharge summaries from structured clinical data. The structured data used to produce AI-generated summaries were manually extracted from electronic health records (EHRs) by a trained medical professional. By leveraging structured documentation collected for research and quality management, the goal is to assist physicians with editable draft summaries. After de-identifying 25 patient datasets, we optimized the output of the LLaMA3 model through prompt engineering and evaluated it using error analysis, as well as quantitative and qualitative metrics. The LLM-generated summaries were rated by physicians on comprehensiveness, conciseness, correctness, and fluency. Key results include an error rate of 2.84 mistakes per summary, and low-to-moderate alignment between generated and physician-written summaries (ROUGE-1: 0.25, BERTScore: 0.64). Medical professionals rated the summaries 3.72 ± 0.89 for comprehensiveness and 3.88 ± 0.97 for factual correctness on a 5-point Likert-scale; however, only 60% rated the comprehensiveness as good (4 or 5 out of 5). Despite overall informativeness, essential details—such as patient history, lifestyle factors, and intraoperative findings—were frequently omitted, reflecting gaps in summary completeness. While the LLaMA3 model captured much of the clinical information, complex cases and temporal reasoning presented challenges, leading to factual inaccuracies, such as incorrect age calculations. Limitations include a small dataset size, missing structured data elements, and the model’s limited proficiency with German medical terminology, highlighting the need for large, more complete datasets and potential model fine-tuning. In conclusion, this work provides a set of real-world methods, findings, experiences, insights, and descriptive results for a focused use case that may be useful to guide future work in the LLM generation of discharge summaries, perhaps especially for those working with German and possibly other non-English content.

**Supplementary Information:**

The online version contains supplementary material available at 10.1038/s41598-025-01618-7.

## Introduction

Clinical documentation serves a variety of purposes, each with specific requirements. Typically, these documentation efforts fall into two categories: structured documentation and free text documentation. In this context, we consider structured documentation to be data stored with reference to a well-defined coding scheme, as opposed to a defined structure within a narrative text. Structured documentation is particularly important when patient data are analyzed across cases, for example in quality assurance, clinical research, or outcome benchmarking. Research relies on structured data for enrolling patients in clinical trials and for quantitative analysis of patient data in general. This is also true for quality assurance in clinical care^1–4^. Structured data are also more accessible to decision support systems, helping to improve patient care.

On the other hand, narrative documentation still plays a vital role in the hospital: At the end of a hospital stay, each patient receives a discharge summary describing medical history that has caused hospitalization, diagnostics, treatment, and the course of the inpatient stay. This important document is written by a physician for each discharged patient and is the basis for post-hospital care^1^. It is typically shared with the patients, their general practitioners, and any relevant outpatient specialists. By facilitating communication between healthcare professionals, the discharge summary plays a vital role in ensuring continuity of care and guiding the patient’s post-hospital treatment^5–7^. Given its importance, both the completeness and accuracy of the discharge summary are essential.

Several studies have examined the impact and quality of structured (tabular, e.g.) discharge summaries as opposed to narrative summaries, which may be richer in describing the individual case^2,3^. Our work is not intended to add to that discussion or to convince physicians to change their documentation practices. In our hospital, it is standard practice to provide a narrative discharge summary for each patient. In addition, a second, structured documentation is maintained for research and quality assurance purposes. We are exploring how we can possibly support medical professionals in such a scenario.

In any case, the process of writing discharge summaries is a time-consuming task for physicians. Arndt et al.^8^ found that physicians spend approximately 44% of their daily working hours on electronic health record (EHR) management, with a significant portion of this time dedicated to documentation tasks. In addition, timely completion of discharge summaries is crucial. These documents should ideally be available on discharge and are for that reason time-critical documents. If the summaries are sent later, the risk of rehospitalization and medication errors might increase^9,10^.

This dual requirement to provide both a comprehensive narrative discharge summary and structured documentation places an additional burden on documenting physicians. To assist physicians and possibly save them some time, automatically generating discharge summaries from structured EHR data could reduce the need for manual writing, allowing physicians to focus more on patient care and clinical decision-making^4^. Several efforts have been made to automate the generation of discharge summaries using natural language generation techniques. Early methods focused on rule-based systems that leveraged text components and medical ontologies^5^. These systems relied on a narrowly defined domain-specific rule base, making it difficult to scale this approach to other areas of medicine. More recently, transformer-based models, such as Bidirectional Encoder Representations from Transformers (BERT) and Bidirectional and Auto-Regressive Transformers (BART), have been employed to classify relevant text and generate summaries based on EHR data^3^. Additionally, generative pre-trained transformer (GPT) models have shown promising results in automating this task^5–8^.

For instance, Aali et al. and Van Veen et al. compared various LLM and benchmarked them based on a task of summarizing free text clinical documents. They compared several LLMs, including LLaMA2-13B and GPT4, against human written texts, both qualitatively and quantitatively^9,10^. Schwieger et al. followed a similar approach by generating psychiatric discharge summaries with ChatGPT-4 from electronic health records^8^. They reported that one of their most significant evaluation questions for physicians was whether the generated summaries were ready for use without manual revision. In contrast, Ellershaw et al. focused on generating discharge summaries based on clinical guidelines and physician notes by means of LLM. For their study, the authors used the Medical Information Mart for Intensive Care III (MIMIC-III) data set and had their results evaluated by clinicians^11,12^.

Despite these advancements, the effectiveness of LLMs varies significantly depending on the domain and language in which they are applied^6^. Many existing models are trained primarily on English datasets that often lack the specialized medical terminology necessary for clinical settings. So far, there are only few approaches for German medical data^7^.

Here, we explore approaches to generating German discharge summaries from structured German clinical data using open-source LLMs and describe the quality of the generated summaries. Unlike other studies, our goal is not to produce a discharge summary that can be sent without further review. Instead, we focus on supporting physicians with a body of text that they can revise during the important process of reflecting on the patient’s course of treatment. This study explores whether structured data, originally collected for science and quality management, can be secondarily used for generating clinical documentation. We utilized EHR data from 25 cases of pancreatic surgery at Heidelberg University Hospital, aiming to evaluate LLM-generated summaries in a non-English medical context. To accomplish this, we applied prompt engineering (PE) techniques to create a tailored prompt, developed a structured data scheme, and successfully generated discharge summaries. This study builds upon prior work that established structured data collection in pancreatic surgery integrating an electronic data collection platform, called “IMI-EDC”. We connected the platform to the established research patient registry of our pancreatic surgery center via API to gain a next generation database with semi-automated data collection^15^. We addressed both the technical and clinical challenges of automated summary generation for this complex surgical field. We conducted a systematic error analysis, as well as both quantitative and qualitative evaluations of the generated summaries to assess the accuracy, completeness, fluency, and relevance compared to those written by physicians, identifying potential improvements and limitations of the automated approach. With this study, we aim to explore a potential pathway for integrating AI-driven documentation tools into clinical workflows, to assist documentation where structured *and* narrative documentation are required.

## Results

### Clinical data included

This study utilized data from 25 patients who had undergone pancreatic surgery at the European Pancreas Center of Heidelberg University Hospital and had been treated in either an inpatient unit or an intermediate care (IMC) unit. Patients requiring admission to the intensive care unit (ICU) were excluded to focus on the majority of standard cases. The data were collected from four primary sources, a patient self-disclosure form and three inpatient documentation forms:

The patient self-disclosure form, filled out by the patients during their initial outpatient pancreatic consultation, contained general demographic information, as well as the patient’s medical and family history. The inpatient documentation forms consisted of three components: the *admission questionnaire* served as a structured summary of the relevant previous medical information by the admitting physician. *Intraoperative documentation* should include details of the surgery directly from the operating surgeon. Finally, the *course of inpatient treatment* should be recorded by the attending ward physician at the point of discharge. At the time of the study, structured data collection using IMI-EDC had not been fully implemented at the Pancreas Center, so we decided to collect data retrospectively from completed cases to investigate the secondary benefit of this data, which was originally collected for a different purpose, for automated clinical documentation. The inpatient documentation was extracted retrospectively from the patients’ unstructured EHR by a medical doctoral candidate with one year of clinical experience (PF). The EHR primarily contained free-text data, such as documentation of the clinical course, histological reports, and radiological result letters. Some information, such as care documentation, was recorded only by hand and was available as a scanned document, which was considered if it was legible. The inpatient stays occurred between January 2023 and March 2024.

A detailed review of existing original discharge summaries of these patients revealed that sections such as “Histologic Findings” had often been copied verbatim from existing documents as well as lists like “previous diagnoses”. In contrast, sections such as “Medical History and Findings” and “Therapy and Course” were largely written in running text by physicians, making them the primary focus of this study. For comparison, a physician-authored discharge summary was available for each case.

We assessed the extent of information available in the abstracted EHR data compared to the physician-written discharge summaries by analyzing 10 randomly selected summaries. For the “Medical History and Findings” and “Therapy and Course” sections, 54% of the content in the physician-written summaries—measured as shared text volume (number of characters)—was present in the structured dataset and could be used by the LLM with a goal to mirror the structure and key elements of physician-authored summaries. This 54% overlap, based on character count, provides a rough approximation of the extent to which structured data aligned with physician-written summaries. However, this metric does not distinguish between clinically meaningful content and redundant or stylistic differences. The remaining 46% may include information that was undocumented, only present in free-text scanned forms, synthesized by the physician, or outside the abstraction scope.

### Data scheme

To create an understandable data structure for the LLM from the available patient data, specific rules were developed in collaboration with an experienced pancreatic surgeon (TMP). Data fields were included only if the associated conditions were met:

1. *Size and weight* The Body Mass Index (BMI) was calculated using the patient’s height and weight. If the BMI fell outside the normal range, both height and weight were added to the data set.
2. *Alcohol consumption* This information was included if the patient had a diagnosis of pancreatitis or reported frequent alcohol consumption.
3. *Tobacco consumption* Data on smoking was included if the patient was a current smoker or had a history of smoking.
4. *Drainages* If the intraoperative drainage was removed within three days of the surgery, it was recorded as “timely removal”. If the drainage was kept for a longer period, the actual removal date was noted.
5. *Laboratory values* The laboratory results were included only if they fell outside the normal range.
6. *Bowel movements* The number of daily bowel movements was included if it exceeded three or if the patient experienced diarrhea or fatty stools.

Additionally, a new data structure was developed, organized into four main sections reflecting the data sources as described above: “General Information”, “Before Surgery”, “During Surgery” and “Inpatient Stay”. This structure organized the data in broad chronological categories to improve its logical flow and comprehensibility. Table 1 shows a corresponding example data set of a patient used to generate the discharge summary. The two columns have the same content, left hand side in English, right hand side in German.

**Table 1:** Example data of a patient (Left Column: English, Right Column: German; Dates were shifted forward by a random, fixed interval).

English	German
*General information* Gender: Male Date of Birth: 07/30/1985 Known Pre-existing Conditions: Arterial Hypertension, Coronary Artery Disease (CAD) Diabetes Mellitus: Diagnosed since 09/2043 Existing Conditions: Colon Polyps (First diagnosed: 09/2045) Smoking Status: Smoker for 30 years, 10 cigarettes/day Alcohol Consumption: Never	*Allgemeine informationen* Geschlecht: männlich Geburtsdatum: 30.07.1985 Bekannte Vorerkrankungen: Arterielle Hypertonie, Koronare Herzkrankheit (KHK) Diabetes mellitus liegt vor seit 09/2043 Vorliegende Veränderungen: Kolonpolypen (Erstdiagnose: 09/2045) Raucherstatus: Raucher: Seit 30 Jahren, Zigaretten pro Tag: 10 Alkoholkonsum: Häufigkeit: Nie
*Pre-surgery information* Reason for Visit: Acute Pancreatitis, Pancreatic Cancer, Intraductal Papillary Mucinous Neoplasms (IPMN), Pancreatic Cyst Date of Initial Diagnosis: 04/03/2045 Current Suspected Diagnosis: Resectable Ductal Adenocarcinoma of the Pancreas (PDAC) Diagnosis Not Histologically Confirmed Unintentional Weight Loss: 30 kg in 24 weeks Chronic Pain: Present for 12 months, severe fluctuating pain, recurrent daily, treated with Morphine Digestion: 10 bowel movements/day, diarrhea, vomiting, pancreatic enzyme intake since 04/2045 Acute Pancreatitis Present: Since 04/2045 Endoscopic Retrograde Cholangiopancreatography (ERCP): Date: 02/12/2045, number of procedures: 1 Recent Nausea Present Laboratory Values: CA 19–9: 42.0 U/ml, elevated; AP: 119.0 U/L, elevated; GGT: 105.0 U/L, elevated Previous Pancreatic Surgery: DHC Stent Placement, Surgery Date: 04/03/2045 Preoperative Treatment: Bile duct stent; Neoadjuvant therapy: Chemotherapy, 6 cycles, completed, last chemotherapy date: 09/25/2045 Radiological Findings of Suspected Diagnosis: PDAC, Resectable Tumor Therapy Recommendation: Explorative Laparotomy, Total Pancreatectomy	*Informationen Vor operation* Grund des Besuchs: Akute Pankreatitis, Pankreaskarzinom, Intraduktale papillär muzinöse Neoplasien (IPMN), Bauchspeicheldrüsenzyste Datum der Erstdiagnose: 03.04.2045 Aktuelle Verdachtsdiagnose: Resektables duktales Adenokarzinom des Pankreas (PDAC) Diagnose histologisch nicht gesichert Ungewollter Gewichtsverlust: 30 kg in 24 Wochen Chronische Schmerzen liegen vor: Beschwerdedauer: Seit 12 Monaten, Schmerzcharakter: Stark schwankende Schmerzen, Schmerzverlauf: Rezidivierend, Schmerzfrequenz: Täglich, Schmerzmedikation: Morphium Verdauung: Anzahl Stuhlgänge pro Tag: 10, Durchfall liegt vor, Erbrechen liegt vor, Einnahme von Pankreasenzymen: seit 04/2045 Akute Pankreatitis liegt vor seit 04/2045 Endoskopisch retrograde Cholangiopankreatikographie liegt vor: Zeitpunkt: 12.02.2045, Anzahl Untersuchungen bisher: 1 Übelkeit liegt in letzter Zeit vor Laborwerte: Carbohydrat-Antigen 19–9 (CA 19–9): 42.0 U/ml: Wert erhöht; Alkalische Phosphatase (AP): 119.0 U/L: Wert erhöht; Gamma-glutamyl Transferase (GGT): 105.0 U/L: Wert erhöht Vorhergehende Pankreas-OP: DHC Stent Einlage, Datum der OP: 03.04.2045 Präoperative Behandlung: Gallengangsstent, Neoadjuvante Therapie: Chemotherapie, Anzahl Zyklen: 6, Vollständigkeit der Chemotherapie: Vollständig, Datum letzte Chemotherapie: 25.09.2045 Radiologischer Befund der Verdachtsdiagnose: PDAC / maligne Pankreastumoren, Resezierbarkeit des Tumors: Resektabel Therapieempfehlung: Explorative Laparotomie, Totale Pankreatektomie
*Surgery information* Hospital Admission Date: 10/12/2045 Surgery Date: 13.10.2045 Confirmed Diagnosis: PDAC Type of Surgery: Total Pancreatectomy, Open Surgery Extended Surgery: Additional multivisceral resection of the stomach Intraoperative Drains: Easy-Flow Drain placed on 10/13/2045, removed on 10/25/2045 Additional Vascular Resection: Portal vein interposition graft, Divestment of Superior Mesenteric Artery, Divestment of Celiac Trunk, Arterial Surgery: Triangle OP Other Intraoperative Details: 800 ml blood loss, transfusion given, antibiotics continued postoperatively	*Informationen Zur Operation* Datum der stationären Aufnahme: 12.10.2045 OP-Datum: 13.10.2045 Diagnose (gesichert): PDAC Art der OP: Totale Pankreatektomie, OP-Zugangsmethode: Offene OP OP wurde erweitert: Zusätzliche multiviszerale Resektion des Magens durchgeführt Intraoperative Drainagen: Easy-Flow Drainage: Eingelegt am 13.10.2045, entfernt am 25.10.2045 Zusätzliche Gefäßresektion: Pfortader Interponat: Sonstiges, Divestment Arteria Mesenterica Superior, Divestment Truncus Coeliacus, Arterielle OP: Traingle OP Sonstige Informationen aus der OP: Transfusion gegeben, Blutverlust von 800 ml, Antibiose weiter postoperativ verabreicht
*Post-surgery course* Post-Surgery Transfer: Recovery room on 10/13/2045, then transfer to IMC Inpatient Antibiotic Therapy: Ceftriaxone/Metronidazole i.v. from 10/12/2045 to 10/16/2045 (perioperative antibiotic); Piperacillin/Tazobactam i.v. from 10/24/2045 to 10/29/2045 (suspected central venous catheter infection) Analgesics Administered: NSAIDs, oral opioids, intravenous opioids, patient-controlled analgesia, regional anesthesia (including epidural) Imaging: CT scan due to infection marker increase, treated with antibiotics Additional Complications: Isolated CRP increase Wound Status: No complications Patient Mobilization: As before surgery Diet Progression: Timely Sufficient Bowel Movement: Since 10/17/2045; vomiting and nausea persisted from day 1 to day 3 postoperatively Other Observations: In addition to a regular diet, parenteral nutrition with Olimel 1500 ml/day was continued to ensure adequate caloric intake and prevent postoperative catabolic metabolism Discharge Date: 10/30/2045	*Stationärer verlauf* Verlegung nach OP in Aufwachraum am 13.10.2045, Verlegung auf IMC Stationäre antibiotische Therapie: Gabe von Ceftriaxon/Metronidazol i.v. von 12.10.2045 bis 16.10.2045, Indikation: Perioperative Antibiose; Gabe von Piperacillin/Tazobactam i.v. von 24.10.2045 bis 29.10.2045, Indikation: Verdacht ZVK Infektion Folgende Analgetika wurden verabreicht: Nicht steroidale Antirheumatika (NSAID), Opioide p.o., Opioide i.v., patientenkontrollierte Analgesie, Regionalanästhesie (inkl. Periduralanästhesie (PDA)) Bildgebung: CT Schnittbild: -Indikation: (Erneuter) Infektwertanstieg, Behandlung durch Gabe von Antibiotika Weitere Komplikationen: Isolierter CRP Anstieg Es zeigten sich reizlose Wundverhältnisse Mobilisation des Patienten war wie vor Operation möglich Der Kostaufbau des Patienten erfolgte zeitgerecht Suffiziente Darmpassage seit 17.10.2045; Erbrechen und Übelkeit ab Tag 1 postoperativ und über den dritten Tag postoperativ hinaus Sonstige Auffälligkeiten: führten zusätzlich zur Vollkost eine parenterale Ernährungstherapie mit Olimel 1500 ml/d weiter, um eine adäquate Kalorienaufnahme zu gewährleisten und eine post-operative katabole Stoffwechsellage zu vermeiden Entlassungsdatum: 30.10.2045

### Prompt engineering

Prompt Engineering (PE) is an iterative and exploratory process driven by both creativity and intuition^8,9^. The literature provides various patterns for structuring prompts, which are employed to enhance the performance of the LLM. Table 2 presents the different prompt patterns we experimented with, along with their corresponding outcomes. The column labeled “improvement” indicates whether any observable improvement in model performance was achieved for each pattern.

**Table 2:** Applied prompt engineering (PE) principles.

PE principle	Description	Application in prompt	Improvement?	Reference
Role	Physician writing for another physician; use precise medical terminology	“You’re a ward doctor at the university hospital and write discharge summaries for the patient’s general practitioner.”	Yes	^10^
	Write in German	“You only speak German.”	Yes	
	Mind the grammar	“You pay attention to the correct use of German grammar.”	No	
Goal	Detailed description of the goal	“Write the sections ‘Medical history and findings’ and ‘Therapy and course’ of a discharge summary for a patient.”	Yes	^10^
Constraints	Output length	“The discharge summary should be approximately 2000 characters long.”	No	^11^
Context Manager	Content description of both paragraphs	“The “Medical History and Findings” section should include the patient’s medical history and explain why the patient received the surgery.”	Yes	^12^
	Decide which information is relevant	“Decide for yourself which information from the patient file is relevant and only use this in the discharge summary.”	No	
	Use placeholder for missing information	“If you are missing information, you can use MISSING as a placeholder”	No	
Template	Structuring the output into the sections “Anamnese und Befund” and “Therapie und Verlauf”	At the end of the summary: “Medical history and findings: Therapy and course:”	Yes	^12^

To evaluate improvements, we first selected a complex test case as a benchmark. The test patient was diagnosed with a resectable ductal adenocarcinoma of the pancreas (PDAC) and underwent a total pancreatectomy via open surgery. The patient was treated on the IMC unit and had a prolonged hospital stay of 18 days. The details of his case are presented in Table 1.

Next, NK prompted the LLM using the various engineered prompt patterns and compared the generated discharge summaries to the physician-written summary. Improvements were noted based on the following criteria: how closely the LLM-generated summary aligned with the physician’s version and its readability in terms of comprehensiveness, conciseness, fluency and factual correctness. If an improvement was observed, the corresponding row in the “improvement” column was marked “Yes”. For example, the ‘Mind the grammar’ prompt was evaluated by comparing the number of grammatical mistakes before and after the change, with a reduction in errors indicating improved fluency. The principle ‘Content description of both paragraphs’ was assessed by comparing whether the revised summary better aligned with the structure of the original physician-written summary—specifically, whether key information was included and placed in the appropriate paragraphs, indicating improved comprehensiveness and conciseness.

Two prompt patterns emerged as particularly useful: The template and the role pattern. The template pattern ensured a consistent structure of the generated discharge summaries, with each summary containing identically formatted sections “Medical History and Findings” and “Therapy and Course”. The role pattern was helpful in maintaining an appropriate medical tone and factual accuracy, ensuring that the summaries were written exclusively in German. The final prompt, incorporating the patterns that demonstrated measurable improvement, is provided in Table 3.

**Table 3:** Final prompt containing all useful prompt engineering principles.

Role	Prompt
System	You are a ward physician at the university hospital and write discharge letters for the patient’s general practitioner. You use correct medical terminology. You only speak German
User	Write the ‘Medical history and findings’ and ‘Therapy and course’ sections of the discharge letter for a patient The ‘Medical history and findings’ section should contain the patient’s medical history and explain why the patient had the operation The ‘Therapy and course’ section describes the course of the operation and information about the patient’s inpatient treatment that is relevant for the general practitioner after discharge All other sections (‘Diagnosis’, ‘Therapy’, ‘Histology’, ‘Procedure’ and ‘Medication’) are created automatically and should not be written by you. Information from the patient’s EHR is available to you for writing the ‘Medical history and findings’ and ‘Therapy and course’ sections Here is the data from the patient file: {data}^a^ Medical history and findings: Therapy and course:

^a^The patient’s data are automatically inserted in place of the placeholder.

The prompt was used in German and was only translated in this paper for reasons of comprehensibility. The original prompt in German is provided in the supplement.

*Prompt Chaining* In addition to standard PE principles, we explored the technique of prompt chaining, which is particularly useful for handling complex, detailed tasks by breaking them down into manageable subtasks. In prompt chaining, the output from one prompt is used as input for the next, creating a sequence of interconnected prompts. This method enhances the model’s controllability and improves the reliability of its responses^13^.

We applied this technique using the initial prompt developed in the previous step (see Table 3), dividing it into discrete sections that were sequentially adapted and executed. By chaining prompts in this way, the model was able to manage more complex cases with promising results, as demonstrated in the supplementary materials.

However, a key challenge with prompt chaining was extracting the final generated discharge summary. The model tended to embed the summary within the ongoing conversation, making it difficult to isolate the final version. Sometimes the most up-to-date summary appeared in the last response, while other times it was found in earlier responses. Table 4 highlights the sections that needed to be extracted, shown in italics. To address this, we developed a pattern-matching approach that reliably extracts the discharge summary in most cases. Our method identifies summaries based on structural features, such as the presence of specific headings (‘History and Findings’ and ‘Therapy and Course’), the organization of text into paragraphs, and the absence of paragraph breaks in the main section. While not perfect, this approach allows for accurate extraction in the majority of cases.

**Table 4:** Example of a generated discharge summary using prompt chaining.

Generated text	
I have reviewed and adjusted the two sections again to ensure that no content is duplicated. Here are the two sections: **History and findings** *The 65-year-old male patient was admitted to our clinic on 30.06.2033 with acute pancreatitis. The medical history revealed previous treatment for colon cancer, which was operated on 03/2033, as well as a colon polyp initial diagnosis on 02/2033. The patient also suffered from arterial hypertension and diabetes mellitus since 02/2032, for which he has been insulin-dependent since 03/2032* **Therapy and course** *The patient underwent an exploratory laparotomy, left pancreatic resection with splenectomy and cholecystectomy on 04/07/2033. The operation was without complications. Following the operation, the patient received pain therapy with opioids p.o. and i.v. as well as regional anesthesia (including epidural anesthesia (PDA)). The wound healed without irritation and the patient could be mobilized as before the operation. The patient’s diet was built up in good time and adequate bowel transit was achieved from 08.07.2033* I hope that these are the corrected sections!	

*In-Context Learning (ICL)* Another promising approach explored in this study was ICL, a lightweight method that integrates example-based learning directly into the model’s prompt during inference. By including in-context examples, the model learned the structure, inputs, and labels of the dataset and could apply this understanding to new test cases^14^.

Building on the prompt from Table 3, we experimented with ICL as an enhancement option. To generate discharge summaries, the prompt was extended using one- and two-shot examples, randomly selected from the dataset. Both complex and simple examples were tested. In a further variation, only the relevant sections of the discharge summaries (“Medical History and Findings,” “Therapy and Course”) were provided, omitting the corresponding structured dataset.

Across all scenarios, it became clear that the model heavily relied on the provided examples, often reproducing sentence structures from them verbatim—no matter if they were contextually appropriate or not. This resulted in discharge summaries containing significantly more errors compared to outputs generated without ICL. Additionally, due to hardware limitations, a maximum of two examples could be included per prompt.

### Discharge summary generation

All 25 discharge summaries were generated successfully, each containing the required running text sections “Medical History and Findings” and “Therapy and Course”. The summaries were written entirely in German and, aside from a few minor details, were readily comprehensible. The generated summaries are displayed in the supplement.

The average time to generate each discharge summary was 112.89 ± 8.19 s, excluding the preprocessing of the structured dataset. The “Medical History and Findings” section had an average length of 698 ± 100 characters, while the “Therapy and Course” section was typically longer, averaging 886 ± 158 characters. In comparison, physician-written discharge summaries had average lengths of 652 ± 374 characters for the “Medical History and Findings” section and 2047 ± 1248 characters for the “Therapy and Course” section.

### Error analysis

*Frequent mistakes* The generated discharge summaries were analyzed for errors, with an average of 2.84 ± 1.71 mistakes found per summary. Below are some frequent types of errors we observed and potential solutions.

1. *Incorrect Age Calculation* in two-thirds of the summaries, the patient’s age was inferred correctly from the date of birth and discharge date. However, in one-third, the age was incorrect. We could resolve this by calculating the age during preprocessing and including it in the structured dataset. We could observe that the incorrectly inferred age calculations not only considered the wrong month and day and were therefore one year off, but several years off. Calculating the age during preprocessing and explicitly including it in the structured dataset could prevent these errors. When we explicitly provided the current date (corresponding to the discharge date) in the prompt, the model was able to determine the age difference in years but could not account for the exact month and day, leading to deviations of up to one year.
2. *Date Confusion* the model often used the date of first diagnosis for the date of first clinical presentation, which led to incorrect dates in half of the cases. However, these two dates were only identical in two cases. Since the first date of clinical presentation was not available in the structured data we collected, it should be added to our future experiments to address this issue. Notably, when we explicitly provided the correct date of first clinical presentation, the model used it correctly, suggesting that the issue arises from missing data.
3. *Pathological Bowel Movements* in 10 cases, when bowel movements per day were provided in the structured dataset, the summaries incorrectly described them as pathological, even when normal (Summary 16). According to the rules in section “Data Scheme”, one to three bowel movements per day were considered physiological. The number of bowel movements mentioned did not affect this misclassification, suggesting that the model did not know this rule.
4. *Imprecise or Incomplete Information* some summaries contained vague or misleading details. For example, one summary inaccurately described the procedure as being stopped and replaced by another, when in fact it was converted to a different type of surgery (Summary 3). In another case, while adjustments were made to the patient’s anti-hypertensive medication, the summary failed to mention that the medication was later discontinued due to intolerance (Summary 16). Additionally, some summaries incorrectly presented cause-and-effect relationships, such as suggesting that the removal of a drainage was part of the treatment for a lymphatic fistula, when it was just a routine step in the patient’s overall recovery process (Summary 6). In total we could observe 4 cases in 3 summaries.
5. *Literal Use of Information from the Structured Dataset* the model often reproduced text directly from the EHR without adjusting for context, such as when referring to the patient’s gender. In multiple instances, it incorrectly used a male pronoun and descriptor for a female patient (Summary 2). To address this, a possible solution would be to modify the language during preprocessing to ensure the text reflects the correct gender.
6. *Grammatical Errors* various grammatical mistakes were identified throughout the summaries. Some examples involved incorrect grammatical cases, such as incorrect article usage (Summary 3). Other issues included improper verb conjugation (Summary 13), as well as the use of uncommon or awkward phrasing that appears to be literal translations from English, which are not typical in German medical language (Summary 11).
7. *Spelling Errors* some summaries contained spelling mistakes, such as misspellings of medical terms (Summary 2), even though the correct versions were available in the structured dataset. These errors are likely caused by inconsistencies in the model’s training data.
8. *Hallucinations* the model occasionally generated false information. For instance, a summary mentioned delayed patient mobilization, while the structured dataset indicated timely mobilization (Summary 3). In another case, nausea was incorrectly described as decreasing from day 3, despite the structured dataset showing it persisted from day 1 onward (Summary 3). In total we could observe 5 cases in 4 summaries.

*Missing Information* We analyzed details that had been present in the structured dataset but were either partially or entirely omitted from the generated discharge summaries. Our analysis aimed to highlight the most commonly missed information and assess the completeness of the summaries.

In the “General Information” section, key details like the patient’s height and weight were consistently absent from the generated discharge summaries, despite being available in the structured dataset. Previous illnesses were included in fewer than half of the cases. Family history was fully or partially included in only 4 cases and omitted in 10. Similarly, information about the patient’s smoking and alcohol consumption habits was missing in approximately three-quarters of the summaries, even when available in the structured dataset.

In the “Before Surgery” section, histological findings were included in only one-third of the summaries, reflecting a significant gap in capturing important preoperative details.

For the “During Surgery” section, intraoperative assessments, such as the condition of the pancreatic parenchyma, were included in only 25% of cases. However, other intraoperative details, such as blood loss and whether a transfusion was required, were at least partially included in two-thirds of the summaries.

In the “Inpatient Stay” section, the transfer of patients from the operating room to recovery, and subsequently to the normal ward or IMC, was included in about two-thirds of the summaries.

Overall, no consistent pattern could be recognized as to which information was included, and which was omitted by the LLM. Determining the clinical relevance of each missing piece of information, and whether it should be consistently included in discharge summaries, requires further evaluation by experienced clinicians.

### Quantitative evaluation

To align the quality of the LLM-generated discharge summaries with physician-written summaries, we employed two widely recognized metrics: the Recall-Oriented Understudy for Gisting Evaluation (ROUGE) score and BERTScore.

The ROUGE score served as standard metric to evaluate summarization tasks and to measure syntactic similarity by comparing n-grams, word sequences, and sentence structures between the generated text and a human-written reference summary^15^. Specifically, we used ROUGE-1 (unigrams), ROUGE-2 (bigrams), and ROUGE-L (longest common subsequences). We also utilized BERTScore, a semantic metric to evaluate the similarity of meanings between the words in the generated and original reference texts.

Table 5 presents the average scores and standard deviations for all calculated metrics:

**Table 5:** Calculated metrics.

Type of score	Score^+^
ROUGE-1	0.25 ± 0.04
ROUGE-2	0.06 ± 0.03
ROUGE-L	0.24 ± 0.04
BERTScore*	0.64 ± 0.01

^+^Mean ± Standard deviation.

*Computed with “facebook/bart-large-mnli”.

ROUGE-1 and ROUGE-L indicated that around 25% of the words or word sequences in the summaries generated matched the reference summaries. ROUGE-2 (0.06 ± 0.03) reflected lower bigram overlap, which could suggest variability in sentence structure and the use of different combinations of words.

The BERTScore of 0.64 showed moderate semantic similarity.

### Qualitative evaluation

**Fig. 1:**
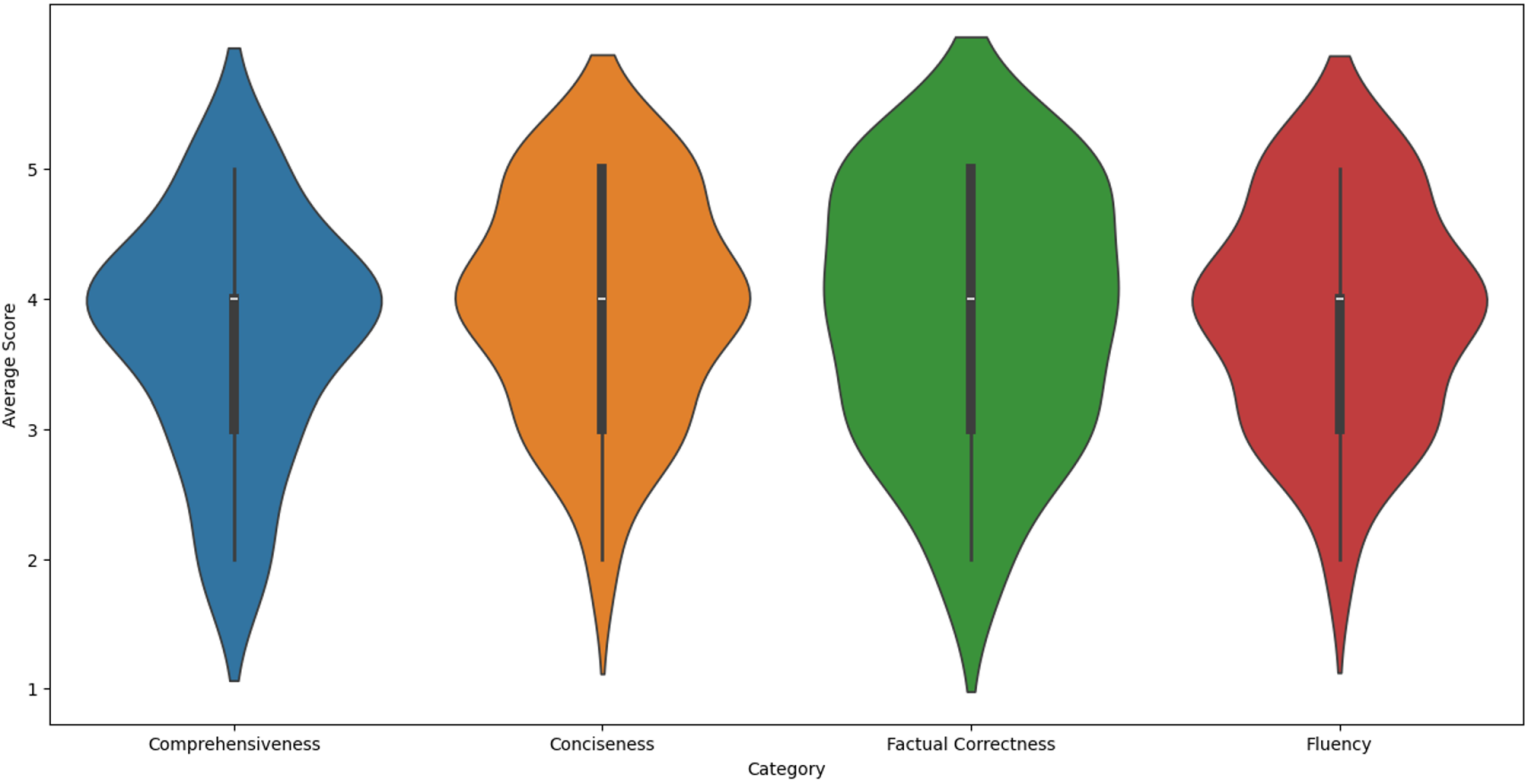
Qualitative evaluation per category; The width of each violin at a given rating value reflects the density of responses. Although most ratings clustered at 4 and 5, a minority of ratings at 2–3 created a visible distribution spread. This helps visualize variability beyond just reporting averages.

A qualitative evaluation of clinicians and medical students provided initial feedback from medical professionals. Five individuals participated in the evaluation, using a questionnaire adapted from Aali et al.^23^ and translated into German. Each participant rated five summaries on four criteria—comprehensiveness, conciseness, fluency, and factual correctness—using a 5-point Likert scale from 1 (very poor) to 5 (very good). For each case, the evaluators were provided with the LLM-generated summary and the corresponding structured input data used to generate it. Figure 1 displays the ratings on each criterion across the five included summaries. Summaries 1, 2, and 4 performed slightly better compared to summaries 3 and 5. Importantly, none of the summaries received a score of 1 (very poor) on any criterion. Notably, summary 3 scored approximately one point lower than the others in the comprehensiveness criterion, indicating that the participants found it to be less detailed or missing key information.

Table 6 displays the mean values and standard deviation for each criterion across all summaries. Overall, the average scores across all criteria ranged from 3.72 to 3.96, with factual correctness and fluency scoring the highest. However, the standard deviation extended below 3 for some criteria. The percentage of ratings classified as “good” (4 or 5) was 60% for comprehensiveness, 72% for conciseness, 80% for factual correctness, and 68% for fluency.

**Table 6:** Qualitative evaluation scores.

Criteria	Score^+^
Comprehensiveness	3.72 ± 0.89
Conciseness	3.96 ± 0.84
Factual correctness	3.88 ± 0.97
Fluency	3.88 ± 0.83

^+^Mean ± Standard deviation.

The findings indicate that while AI-generated summaries have potential, certain limitations remain. Specifically, the model occasionally oversimplifies complex clinical cases and also exhibits significant issues with completeness—likely due to missing or insufficient input data, and potentially related to the same mechanisms that drive oversimplification.

## Discussion

This study introduces several new findings in the application of LLMs for generating German-language discharge summaries from structured datasets. It is among the first to assess how well LLMs can capture clinically relevant information in German, and shows that summaries generated by the LLaMA3 model can be a first step toward aligning with physician-written summaries in both structure and content. This might eventually lead to added value for clinicians by collecting structured data through secondary use for automated physician letter writing. The data were collected using the modern data capture platform IMI-EDC, also with a scientific focus.

Our results suggest that structuring the input data into broad chronological categories and applying specific inclusion rules may help organize the summary content. However, handling complex cases, such as intermediate care (IMC) patients, remains challenging for the model. Additionally, PE and prompt chaining techniques seem to help enhancing summary quality, albeit with considerable processing time.

Compared to the literature, our study underscores similar challenges in data completeness and consistency seen in LLM-generated summaries. About 46% of the content typically found in physician-written summaries, measured as shared text-volume, was missing from the structured dataset. This echoes findings from earlier research, which suggest that some clinically relevant information resides only in physicians’ notes or unstructured data sources outside the EHR^16^. This finding also aligns with our qualitative evaluation, which showed that only 60% of summaries were rated “good” in terms of comprehensiveness.

Our study supports previous research findings on the limitations and potentials of PE and in-context learning (ICL) in improving LLM performance in complex data-to-text tasks. For instance, while ICL has demonstrated potential in improving model performance on specific tasks, our findings echo Reynolds and McDonell’s^17^ observation that examples do not always improve model outputs, particularly for complex and nuanced tasks like clinical documentation. Overall, iterative prompt engineering and prompt chaining were judged as more fruitful than ICL for generating higher quality summaries.

The model’s settings were tuned to optimize performance. As recommended in existing literature^18–20^, a low temperature setting was used to minimize hallucinations and ensure a more objective output. While this resulted in fewer factual inaccuracies, slightly increasing the temperature improved fluency and readability, suggesting the need for fine-tuning based on specific use cases. Careful testing of these parameters remains essential.

Some errors made by the model—such as the literal use of information from the structured dataset, grammatical errors, and spelling errors—only marginally affect the comprehensibility of the generated discharge summaries. These issues primarily stem from the model’s limited proficiency in medical terminology and German.

A more significant challenge is the incorrect age calculation observed in one-third of the cases. As noted by Tan et al.^21^, many LLMs struggle with temporal reasoning. In our analysis, we found that while LLaMA3 can accurately compute the difference between two given dates as a standalone task, it fails to apply this ability when generating full discharge summaries that require multiple reasoning tasks. This limitation arises because LLMs do not inherently possess a structured understanding of time; instead, they rely on pattern recognition from their training data rather than explicit date arithmetic. Tan et al.^21^ highlight that LLMs often default to heuristics, such as assuming that later-mentioned dates are more relevant or interpreting temporal relationships based on co-occurrence statistics rather than logical computation. These biases may explain why the model occasionally miscalculates the patient’s age.

The LLaMA3 model^24,25^ used in this study showed strong performance in handling minor inconsistencies in the structured dataset. For instance, despite the presence of conflicting information—such as antibiotics being documented as administered only intraoperatively in one section of the EHR and recorded again in the inpatient course—the model correctly synthesized this and included antibiotic administration in the generated discharge summary (Summary 3).

Hallucinations, vague or misleading statements, or otherwise imprecise information were identified in 9 out of 25 summaries. This refers to individual content issues, not the overall completeness of each summary. Such errors are particularly difficult to detect, making mitigation strategies essential. One potential approach is the integration of Retrieval-Augmented Generation (RAG)^22^, which could help reduce inaccuracies caused by a lack of medical knowledge—such as the misclassification of bowel movement frequency—by incorporating up-to-date information from literature and clinical guidelines. Some errors may arise due to issues with reasoning or context integration. In these cases, implementing Human-in-the-Loop (HITL) validation within clinical workflows could provide real-time oversight. A potential HITL workflow could involve generating a draft discharge summary that is then reviewed and corrected by a physician before being finalized and handed to the patient. The data gathered from these HITL interactions could also be leveraged for reinforcement learning with human feedback (RLHF) to further refine model outputs in future research^23^.

For quantitative evaluation we applied ROUGE and BERTScore as commonly used in related research and while they provided valuable insights into syntactic and semantic similarity, they were limited in capturing clinical relevance and domain-specific language quality. As highlighted by Jung et al.^24^, these metrics do not account for critical aspects of medical documentation, such as the correct use of medical terminology, underscoring the need for clinically oriented evaluation metrics.

We achieved low to moderate ROUGE and BERTScore scores compared to the literature. However, it is difficult to compare the absolute metrics with other studies because we compared the generated summaries with those written by physicians. Most other studies use these metrics as relative values to compare summaries generated by different language models on the same base text within the self-contained systems of their studies^9,23,27^. In particular, ROUGE-L could suffer from our approach of generating texts from structured data, which are typically short concepts that do not lead to long overlaps with texts written by physicians. Our qualitative survey findings are in line with those of Aali et al.^24^, who conducted a similar survey. Although they do not report numbers, according to the violin plots of their publication, GPT-4 achieved the highest ratings (average 4–5, small standard deviation), while LLaMA2-13B and physician-written summaries were rated lower, but at a comparable level (comprehensiveness/factual accuracy: average 2–3, very large standard deviation; conciseness/fluency: 3–4, large standard deviation). Considering Table 6 the LLaMA3 model used in our study appears to fall between GPT-4 and LLaMA2 in terms of performance across all criteria.

However, several limitations must be considered. First, the relatively small dataset of 25 cases restricted both the diversity of clinical contexts and the generalizability of our findings. This sample size limits our ability to fine-tune models with techniques like Quantized Low-Rank Adaptation (QLoRA)^25^, which would likely improve LLM performance with larger datasets. Additionally, inconsistencies, missing structured data, and errors in the raw EHR data highlight the challenges of retrospective data collection. However, the completeness of LLM-generated discharge summaries depends upon the completeness and/or curation of the data fed to the LLM. The model also showed limitations in handling missing information, especially when critical details—such as hand-written notes or non-EHR information—were absent from the structured input data. This missing data reduced the accuracy of the summaries generated, underscoring the need for more comprehensive data integration in future studies. Further, the ability to incorporate a larger number of ICL examples was constrained by GPU limitations, potentially impacting the model’s performance. Also, model-generated summaries exhibited issues with German grammar, spelling, and medical terminology, underscoring the need for model fine-tuning on medical data or integrating RAG^28^, which could improve the model’s medical competency and reduce the need for manual corrections^32^. Additionally, alternative models, such as GPT-4, which performed well in similar studies^24,33^, may have produced better results in our context. The rapid evolution of LLMs also suggests that new models in the near future may significantly outperform those used in the current study, potentially addressing some of the limitations observed here.

Future research should consider larger datasets and improved data collection methods to enhance the diversity and completeness of training data, which could improve model accuracy and reduce the need for physician oversight. Expanding data sources to include unstructured clinical texts and nursing notes could also enrich input quality, addressing limitations due to incomplete raw EHR data. Testing alternative model fine-tuning approaches, such as QLoRA, or incorporating RAG could enhance domain-specific accuracy and medical terminology use. Using ICL with an increased and carefully selected number of examples can have a significant impact and should therefore be tested with more eligible hardware^26,27^. More extensive qualitative evaluations are also needed, involving a broader group of healthcare professionals to evaluate the practical relevance, acceptance, and error identification in automatically generated summaries. Future research should also address questions of time efficiency, acceptance among clinicians, and the ability to categorize errors in generated content^20^. With improved model accuracy and clinically oriented evaluation metrics, LLMs could offer a viable tool for discharge documentation in real-world settings.

## Conclusion

Our study describes the application of an open-source LLM to generate German-language discharge summaries from structured clinical data in a real-world setting. The approach demonstrated the ability to produce coherent and moderately accurate drafts. However, our data also underscore significant limitations—particularly in temporal reasoning, error-prone verbatim copying in ICL, and inconsistent inclusion of clinically relevant content. These findings highlight that while LLM-based tools may one day be useful for supporting the clinical documentation process, achieving high-quality outputs remains dependent on multiple factors, including data completeness, task framing, and post-processing pipelines. Our methods, findings, and descriptive analyses can inform future research and implementation efforts, particularly in the context of non-English medical documentation.

## Methods

### LLM selection

To generate discharge summaries from raw EHR data, we first identified a suitable LLM based on specific requirements: the model needed to be open source for transparency and local deployment, compatible with available GPU constraints, able to generate text from structured data, support German language comprehension, handle medical terminology, and maintain a context window of at least 4000 tokens.

We reviewed domain-specific literature, consulted experts, and evaluated models using leaderboards like Open Medical LLM Leaderboard. SauerkrautLM^28^, OpenBioLLM^29^, and LLaMA3^30^ emerged as the top candidates. OpenBioLLM scored highly in medical comprehension, particularly on medical multiple-choice benchmarks, suggesting suitability for clinical language tasks. SauerkrautLM, fine-tuned on German texts, demonstrated strong language proficiency. LLaMA3, with a robust multilingual foundation, showed versatility in generating high-quality German medical text without domain-specific fine-tuning. All three models support an 8192-token context window.

While GPT-4 is widely used in comparable research^18–20^, it was excluded from consideration due to the requirement for an open-source model that could be deployed locally, ensuring compliance with strict data protection protocols.

Due to its high performance on our qualitative criteria (comprehensiveness, conciseness, factual correctness, and fluency), LLaMA3 was selected for this project.

### Data preprocessing and prompt engineering

For this study, a total of 25 datasets from completed cases from the European Pancreas Center of Heidelberg University Hospital were retrospectively collected by a medical doctoral candidate (PF, one year clinical practice) and recorded in two distinct forms: a self-disclosure form and an inpatient documentation form. The data was collected with the electronic data capture tool “IMI-EDC” and exported as CSV file. The use of the hospital data was conducted in accordance with the Declaration of Helsinki and approved by the Ethics Committee of the medical faculty at the University of Heidelberg (S301/2001; S708/2019; S083/2021).

To protect patient privacy, to the extent feasible and as described below, all protected health information (PHI) was removed. While the German Data Protection Regulation does not specifically define how to de-identify clinical documents, we adhered to best practices established by the Health Insurance Portability and Accountability Act (HIPAA)^31^, similar to other German and European studies^32–34^. This process involved an initial automated PHI removal using a pattern-matching approach to identify and redact personal identifiers such as names, titles, organizations, histological findings, locations, and phone numbers. In a second step, any remaining PHI was manually reviewed and removed. Additionally, dates were shifted forward by a random, fixed interval—adjusting the day, month, and year—to further anonymize the data, in line with HIPAA standards^31^.

The design of the prompts for the LLM followed an iterative process to determine the most effective approach. Various principles identified in the literature were tested on a sample case. The generated discharge summaries were evaluated based on qualitative criteria, including comprehensiveness, conciseness, factual accuracy, and fluency. Principles that led to improvements in the output were retained, while those that did not were discarded.

Additionally, we tested two advanced techniques, Prompt Chaining and ICL, which may further enhance prompt effectiveness. Prompt Chaining is a method used to handle complex tasks by breaking them into smaller, more manageable subtasks. The model’s output from one prompt serves as the input for the next, creating a chain of prompts. This approach can increase both the controllability and reliability of the model’s responses. For this study, the initially developed prompt (see Table 3) was divided into five stages: first, the model was asked to organize the provided structured dataset; next, it generated the “Medical History and Findings” section; followed by the “Therapy and Course” section; fourth, it combined and refined both sections and finally, the content was reviewed by comparing it against the original data. The complete chat, including all prompts, is provided in Table 2 of the supplementary material. For ICL, we provided the model with one- or two-shot examples, which were included in the prompt to serve as references for the task at hand. These examples were intended to guide the model and help it adapt its responses by learning from the patterns and structures presented in the examples.

The structured data provided was transformed into a rule-based natural language description of each feature. Through an iterative process, an optimal structure and sequence were developed to guide the LLM. This involved adding explanatory information for specific data points and formulating conditional rules under which certain attributes were made available to the model.

### Model implementation and setup

The preprocessed data, along with the prompt developed during the PE process, was used with the LLaMA3 chat template to generate the discharge summaries. The generation process followed these steps for each patient record:

1. Prompt Generation:
  Abstracted EHR Data Preprocessing: The structured data was extracted from the completed forms and formatted according to the structure presented in Table 1.
  Prompt Formatting: The extracted data was inserted into the predefined prompt structure (as shown in Table 3), replacing the {data} placeholder.
2. Summary Generation:
  The formatted prompt was processed by LLaMA3 using the specified chat template to generate the discharge summary.
3. Summary Evaluation:
  The generated summary was evaluated as described in the ‘Model Evaluation’ section.

- For text generation, the following libraries and versions were employed: Transformers (Version 4.39.3)
- PyTorch (Version 2.2.2)
- BitsAndBytesConfig (Version 0.43.1) for 4-bit quantization and bfloat16 inference.

The Python (v3.11.5) script ran on an NVIDIA RTX A6000 GPU (48 GB), with hyperparameters set to a temperature of 0.2 for deterministic outputs and a 1000-token limit for new content generation.

### Model evaluation

*Error Analysis* Where possible, underlying causes or explanations for errors were explored, along with potential strategies for improvement. Additionally, the average number of errors per summary was calculated to quantify the model’s performance.

The error analysis was conducted by one of the authors (NK), with a second author (TMP) consulted in cases of uncertainty. The analysis of frequent mistakes involved a systematic comparison between structured data from the EHR and the generated summaries. An error was defined as any piece of information in the summary generated that either did not match the corresponding structured dataset or could not be inferred from it. All identified errors were labeled within the respective summaries, recurring errors were categorized.

Furthermore, instances where information present in the structured dataset was absent from the generated summaries were identified and marked in the structured datset. This “missing information” was defined as any information available in the structured EHR dataset but not included in the generated summary.

*Quantitative Evaluation* Summary quality was evaluated using BERTScore and ROUGE by comparing the generated summaries to the physician-written summaries. BERTScore assessed semantic similarity using the BERTScore library (v0.3.13) and contextualized embeddings from “facebook/bart-large-mnli”^35^. ROUGE metrics (ROUGE-1, ROUGE-2, and ROUGE-L) measured n-gram overlaps and sequence commonalities. Consistent with prior literature, both ROUGE (syntactic similarity) and BERTscore (semantic similarity) were used^9,10,23,27^.

*Qualitative Evaluation* Physicians and medical students conducted an anonymous survey evaluating five randomly selected summaries, along with their corresponding structured dataset. The survey consisted of four questions, adapted from Aali et al.^18^ and translated into German. Respondents rated each summary on a 5-point Likert scale, ranging from 1 (very poor) to 5 (very good), based on the following criteria:

1. Comprehensiveness: how well does the summary capture important information? This assesses the recall of clinically significant details from the input text.
2. Conciseness: how well does the summary exclude non-important information? This compares how well the summary is condensed, considering the value of a summary decreases with superfluous information.
3. Factual Correctness: how well does the summary agree with the facts outlined in the clinical note? This evaluates the precision of the information provided.
4. Fluency: how well does the summary exhibit fluency? This assesses the readability and natural flow of the content.

The goal of the evaluation was to gather preliminary insights into how well the LLaMA3-generated summaries align with clinical expectations.

## Electronic supplementary material

Below is the link to the electronic supplementary material.

Supplementary Material 1

## Data Availability

The datasets used and/or analysed during the current study are available from the corresponding author on reasonable request.

## Code availability

The underlying code for this study is available on GitHub and can be accessed via this link https://github.com/IMI-HD/llm-discharge-summaries.
