# Supplementary material for: Automated generation of discharge summaries: leveraging large language models with clinical data
Source: Sci Rep. 2025 May 12;15:16466. doi: 10.1038/s41598-025-01618-7 (PMC12069548; doi:10.1038/s41598-025-01618-7)
Supplement: Supplementary file 1 — Supplementary Material 1 [file 41598_2025_1618_MOESM1_ESM.pdf]

Supplementary Materials

Data Schema

Table 1, 2, 3 and 4 show the data schema. The names of the data items are provided in English and German. An example for each item is provided in German only.

Table 1: Data Scheme: General Information

**Data Item *[English]***	**Data Item *[German]***	**Example *[German]***
Gender	Geschlecht	Männlich
Date of Birth	Geburtsdatum	01.01.2033
Body weight & height	Körpergewicht & Körpergröße	171cm, 73kg
Pre-existing conditions	Vorerkrankungen	Arterielle Hypertonie, COPD
Diabetes	Diabetes	Diabetes Mellitus, liegt vor; Einnahme oraler Antidiabetika
Existing Changes	Vorliegende Veränderungen	Uterusmyom liegt vor
Extrapancreatic tumour diseases	Extrapankreatische Tumorerkrankungen	Vorliegen extrapankreatische Tumorerkrankungen: Lugenkrebs: Therapie: Chemotherapie, Zeitpunkt: 01/2033
Previous abdominal extrapancreatic surgery	Vorhergehende abdominelle extrapankreatische OP	Vorgehende abdominelle extrapankreatische OP liegt vor
Family history pancreas	Familienanamnese Pankreas	Familienanamnese: Chronische Pankreatitis liegt vor: Betroffene Familienmitglieder: Mutter
Family history cancer	Familienanamnese Krebs	Familienanamnese: Extrapankreatische Krebserkrankungen: Lungenkrebs liegt vor: Betroffene Familienmitglieder: Onkel
Nicotine & Alcohol	Nikotin & Alkohol	Raucherstatus: Ehemaliger Raucher: Aufgehört am: 01/2033, Anzahl Jahre: 40, Zigaretten pro Tag: 15<br>Alkoholkonsum: Häufigkeit: Aufgehört seit: 01/2033, Häufigkeit Konsum vor Aufhören: Gelegentlicher Konsum, Übermäßiger Alkoholkonsum seit 01/2033 bis 11/2033

State of health & quality of life	Gesundheitszustand & Lebensqualität	Gesundheitszustand in der letzten Woche: 3 (1=sehr schlecht, 7=ausgezeichnet); Lebensqualität in der letzten Woche: 5 (1=sehr schlecht, 7=ausgezeichnet)

Table 2: Data Scheme: Before Surgery

**Data Item *[English]***	**Data Item *[German]***	**Example *[German]***
Reason for visit (pancreas consultation)	Grund des Besuchs (Pankreassprechstunde)	Erstvorstellung; akute Pankreatitis,
Recurrence	Rezidiv	Rezidiv festgestellt am 01.01.2033
Date of initial diagnosis	Datum Erstdiagnose	01.01.2033
Suspected diagnosis	Verdachtsdiagnose	PDAC
Histologically confirmed diagnosis	Histologisch gesicherte Diagnose	Diagnose histologisch gesichert: Material: Metastasen-Probenentnahme, Befund: PDAC
Weight loss in weeks	Gewichtsverlust in Wochen	7 kg in 3 Wochen
Pain history	Schmerzanamnese	Chronische Schmerzen liegen vor: Schmerzcharakter: Leichte Schmerzen, Schmerzverlauf: Rezidivierend, Schmerzfrequenz: Wöchentlich, Schmerzmedikation: Morphium
Digestion	Verdauung	Anzahl Stuhlgänge pro Tag: 3, Erbrechen liegt vor
Pancreatitis (acute & chronic)	Pankreatitis (akut & chronisch)	Chornische Pankreatitis liegt vor seit 01.01.2033
Jaundice & ERCP	Ikterus & ERCP	Ikterus liegt vor seit 01.01.2033
Laboratory values	Laborwerte	Bilirubin gesamt: 1.16 mg/dl: Wert erhöht
Previous pancreatic surgery	Vorhergehende PankreasOP	Vorhergehende Pankreas-OP: DHC Stent Einlage, Datum der OP: 01.01.2033

Previous therapy (chemo)<br>Preoperative treatment	Vorausgegangene Therapie (Chemo)<br>Präoperative Behandlung	Präoperative Behandlung: Gallengangsstent
Preoperative bile duct drainage	Präoperative Gallengangsdrainage	Präoperative Gallengangsdrainage gelegt
Radiological findings of the suspected diagnosis	Radiologischer Befund der Verdachtsdiagnose	Radiologischer Befund der Verdachtsdiagnose: Duktales Adenokarzinom des Pankreas (PDAC) / maligne Pankreastumoren, Resezierbarkeit des Tumors: Resektabel
Other radiological findings	Sonstige radiologische Befunde	Sonstige radiologische Befunde: Zeichen einer akuten Pankreatitis
Treatment recommendation	Therapieempfehlung	[Freitext]

Table 3: Data Scheme: During Surgery

**Data Item *[English]***	**Data Item *[German]***	**Example *[German]***
Date of admission	Aufnahmedatum	01.01.2033
Date of surgery	OP-Datum	01.01.2033
Pancreatic disease	Pankreaserkrankung	PDAC
Extent of surgery	OP-Ausmaß	Art der OP: Totale Pankreatektomie, OP wurde abgebrochen: Grund: Metastasen
Surgery extension	OP-Erweiterung	OP wurde erweitert: Zusätzliche Leberresektion durchgeführt
Intraop drains (incl. date of removal)	Intraop-Drainagen (inkl. Entfernungsdatum)	Intraoperative Drainagen: Spüldrainage: Eingelegt am 01.01.2033, entfernt am 11.01.2033
Foreign material: (drains), gastric tube, bile duct, pancreatic duct	Fremdmaterial: (Drainagen), Magensonde, Gallengang, Pankreasgang	Fremdmaterial vorhanden: Externalisierung Gallengang
Vascular resection	Gefäßresektion	Zusätzliche Gefäßresektion: Divestment A. Hepatica

Pancreas evaluation	Bewertung Pankreas	Intraoperative Bewertung des Pankreasparenchyms/-gewebes: Pankreas nicht lipomatös
Frozen section	Schnellschnitt	Schnellschnitt erfolgt, Auffälligkeiten: [Freitext]
Other: transfusion, blood loss, stomach/colon/... at risk, antibiosis, special features	Sonstiges: Transfusion, Blutverlust, Magen/Colon/.. gefährdet, Antibiose, Besonderheiten	Sonstige Informationen aus der OP: Transfusion gegeben, Blutverlust von 800 ml, Antibiose nur intraoperativ verabreicht

Table 4: Data Scheme: Inpatient Course

**Data Item *[English]***	**Data Item *[German]***	**Example *[German]***
Transfer after surgery in recovery room to IMC, from IMC to normal	Verlegung nach OP in Aufwachraum auf IMC, von IMC auf Normal	Verlegung nach OP in Aufwachraum am 30.06.2038, dann zeitnahe Verlegung auf Normalstation
Antibiotics (type / form / duration / indication)	Antibiotika (Art / Form / Dauer / Indikation)	Stationäre antibiotische Therapie: Gabe von Pivmecillinam von 01.01.2033 bis 11.01.2033
Antifungal therapy (type / form / duration / indication)	Antimykotische Therapie (Art / Form / Dauer / Indikation)	Stationäre antimykotische Theraoie: Gabe von Caspofungin i.v. von 01.01.2033 bis 11.01.2033
Antivirals (type / form / duration / indication)	Virostatika (Art / Form / Dauer / Indikation)	Stationäre virostatische Therapie: Gabe von Aciclovir i.v. von 01.01.2033 bis 11.01.2033
Analgesics	Analgetika	Folgende Analgetika wurden verabreicht: Opioide p.o., Opioide i.v.
Further Medikation	Weitere Medikation	Weitere Medikation: Gabe von Metoclopramid p.o. von 01.01.2033 bis 01.01.2033
Keratinin level	Keratininspiegel	Persistierend erhöhter Keratininspiegel
CT indication	CT Indikation	Bildgebung: CT Schnittbild:<br>-Indikation: (Erneuter) Infektwertanstieg, Infektwertanstieg wurde beobachtet

CT sectional images	CT Schnittbilder	Befund: Verhalt im Bereich der Pankreasanastomose: Interventionelle Drainagenanlage erfolgt
Pancreatic fistula	Pankreasfistel	Pankreasfistel: Biochemical Leak (Grade A)
Other complications (e.g. surgical side infection, bile leakage, ...)	Weitere Komplikationen (z.B. Surgical Side Infection, Galleleckage, …)	Weitere Komplikationen: Lymphfistel, Begründung: Triglyceride mehr im Sekret
Revision surgery	Revisionseingriff	Revisionseingriff erfolgt: Indikation: [Freitext], Datum: 01.01.2033, Art des Eingriffs:[Freitext]
Wound healing	Wundheilung	Es zeigten sich reizlose Wundverhältnisse
Mobilisation	Mobilisation	Mobilisation des Patienten war wie vor Operation möglich
Diet build-up	Kostaufbau	Der Kostaufbau des Patienten erfolgte verzögert
Intestinal passage	Darmpassage	Suffiziente Darmpassage seit 01.01.2033; Erbrechen und Übelkeit ab Tag 1 postoperativ und über den dritten Tag postoperativ hinaus
Other abnormalities	Sonstige Auffälligkeiten	[Freitext]
Discharge dates	Entlassungsdatum	01.01.2033

Prompt

The German prompt in Table 5 was used to generate the discharge summaries. The translated prompt is available in Table 3 in the manuscript itself.

Table 5: German Prompt

Role	Prompt
System	Du bist Stationsarzt im Universitätsklinikum und schreibst Entlassungsbriefe für den Hausarzt des Patienten. Dabei verwendest du korrekte medizinische Terminologie. Du sprichst ausschließlich Deutsch.

User	Schreibe die Abschnitte „Anamnese und Befund“ und „Therapie und Verlauf“ des Entlassungsbriefs für einen Patienten.<br>Der Abschnitt „Anamnese und Befund“ soll die Vorgeschichte des Patienten enthalten und erklären, warum der Patient die Operation erhalten hat.<br>Der Abschnitt „Therapie und Verlauf“ beschreibt den Verlauf der Operation und Informationen über die stationäre Behandlung des Patienten, die nach der Entlassung für den Hausarzt relevant sind.<br>Alle anderen Abschnitte („Diagnose“, „Therapie“, „Histologie“, „Procedere“ und „Medikation“) werden automatisch erstellt und sollen nicht von dir ge-schrieben werden. Um die Abschnitte „Anamnese und Befund“ und „Therapie und Verlauf“ zu schreiben, stehen dir Informationen aus der Patientenakte zur Verfügung.<br>Hier sind die Daten aus der Patientenakte:<br>{data}[1]<br>Anamnese und Befund:<br>Therapie und Verlauf:

[1]The patient’s data is automatically inserted in place of the placeholder.

Prompt Chaining

Table 6 illustrates the full prompt chaining process: the system prompt initiates the sequence, followed by a user prompt, which is then answered by the assistant (LLaMA3). This user-assistant interaction is repeated five times in total.

Table 6a: Prompt Chaining Example(German)

Role	Prompt
System	Du bist Stationsarzt im Universitätsklinikum und schreibst Entlassungsbriefe für den Hausarzt des Patienten. Dabei verwendest du korrekte medizinische Terminologie.
User	Verwende die nachfolgenden Patientendaten und strukturiere sie für einen späteren Entlassungsbrief.<br>{data}[1]
Assistant	Hier ist der Entlassungsbrief für den Hausarzt des Patienten:<br>**Entlassungsbrief**<br><br>**Patientendaten:**<br>* Name: [Name des Patienten]<br>* Geschlecht: männlich<br>* Körpergröße: 175 cm<br>* Körpergewicht: 65 kg<br><br>**Diagnose:**<br>* Akute Pankreatitis<br>* Verdachtsdiagnose: Duktales Adenokarzinom des Pankreas (PDAC) / maligne Pankreastumoren, cTNM-Klassifikation: pT2 pN0<br>* Histologische Diagnose: Neuroendokriner Tumor (NET)<br>**Vorhergehende Erkrankungen:**<br>* Arterielle Hypertonie<br>* Diabetes mellitus seit 02/2032, Insulinpflicht seit 03/2032<br>* Darmkrebs, operiert am 03/2033<br>* Kolonpolypen, Erstdiagnose am 02/2033

* Vorhergehende abdominelle extrapankreatische OP
Stationärer Aufenthalt:
* Datum der Erstdiagnose: 30.06.2033
* Datum der stationären Aufnahme: 03.07.2033
* OP-Datum: 04.07.2033
* Entlassungsdatum: 13.07.2033

Operation:
* Explorative Laparotomie, Pankreaslinksresektion mit Splenektomie, Cholezystektomie
* OP-Zugangsmethode: Offene OP
* Intraoperative Bewertung des Pankreasparenchyms/-gewebes: Textur: Hart, Pankreas nicht lipomatös
* Blutverlust von 300 ml, V.coronaria durchtrennt, Antibiose nur intraoperativ verabreicht

Postoperative Entwicklung:
* Verlegung nach OP in Aufwachraum am 04.07.2033
* Intraoperative Drainagen: Easy-Flow Drainage, entfernt am 10.07.2033
* Analgetika: Opioide p.o., Opioide i.v., Regionalanästhesie (inkl. Periduralanästhesie (PDA))
* Reizlose Wundverhältnisse
* Mobilisation des Patienten war wie vor OP möglich
* Kostaufbau des Patienten erfolgte zeitgerecht
* Suffiziente Darmpassage seit 08.07.2033

Sonstige Auffälligkeiten:
* Blutdruckspitzen von systolisch bis zu 200 mmHg, Anpassung der Antihypertensiva sowie das Neuansetzen von Bisoprolol 5 mg 1-0-0 durchgeführt, hierunter zeigten sich die Blutdruckspitzen deutlich kupiert.
Laborwerte:
* Carcinoembryoonales Antigen (CEA): 4.2 mikrogramm/L (erhöhter Wert)
* Carbohydrat-Antigen 19-9 (CA 19-9): 8.1 U/ml (Wert im Normbereich)
* Bilirubin gesamt: 1.98 mg/dl (erhöhter Wert)
* Alkalische Phosphatase (AP): 71.0 U/L (Wert im Normbereich)
* Gamma-glutamyl Transferase (GGT): 33.0 U/L (Wert im Normbereich)
* Amylase: 39.0 U/L (Wert im Normbereich)
* Lipase: 42.0 U/L (Wert im Normbereich)

Therapieempfehlung:
* Weitere Nachsorge und Kontrolle empfohlen
Bitte beachten Sie, dass dieser Entlassungsbrief nur als Beispiel dient und vor der Verwendung auf die spezifischen Bedürfnisse des Patienten und die tatsächlichen Umstände des Krankenhausaufenthalts angepasst werden muss.

User — Schreibe einen Abschnitt mit der Überschrift „Anamnese und Befund“. Er ist Teil eines Entlassungsbriefs und soll alle Informationen enthalten, die erklären, warum der Patient die Operation erhalten hat. Verwende dazu die Informationen, die ich dir gegeben habe.

Assistant — Hier ist der Abschnitt "Anamnese und Befund" des Entlassungsbriefs:

Anamnese und Befund
Der 65-jährige männliche Patient wurde am 30.06.2033 mit einer akuten Pankreatitis in unsere Klinik aufgenommen. Die Anamnese ergab eine Vortherapie wegen eines Darmkrebses, der am 03/2033 operiert wurde, sowie eine Kolonpolypen-Erstdiagnose am 02/2033. Der Patient litt

auch an einer arteriellen Hypertonie und einem Diabetes mellitus seit 02/2032, für den er seit 03/2032 insulinpflichtig ist.
Bei der Aufnahme zeigte sich eine akute Pankreatitis, die radiologisch als duktales Adenokarzinom des Pankreas (PDAC) / maligne Pankreastumoren, cTNM-Klassifikation: pT2 pN0, diagnostiziert wurde. Die Laborwerte zeigten eine Erhöhung des Carcinoembryonalen Antigens (CEA) und des Bilirubins, während die anderen Werte im Normbereich lagen.
Die intraoperative Untersuchung ergab eine Neuroendokriner Tumor (NET) des Pankreas. Der Patient wurde einer explorativen Laparotomie, Pankreaslinksresektion mit Splenektomie und Cholezystektomie unterzogen. Die Operation verlief ohne Komplikationen, und der Patient konnte nach einer stationären Aufenthaltsdauer von 10 Tagen entlassen werden.

User Schreibe einen Abschnitt mit der Überschrift „Therapie und Verlauf“. Er ist Teil des Entlassungsbriefs und beinhaltet Informationen über die stationäre Behandlung des Patienten. Der Abschnitt soll nur Informationen enthalten, die nach Entlassung des Patienten für den Hausarzt relevant sind.

Assistant Hier ist der Abschnitt "Therapie und Verlauf" des Entlassungsbriefs:

Therapie und Verlauf
Der Patient wurde am 04.07.2033 einer explorativen Laparotomie, Pankreaslinksresektion mit Splenektomie und Cholezystektomie unterzogen. Die Operation verlief ohne Komplikationen.
Im Anschluss an die Operation erhielt der Patient eine Schmerztherapie mit Opioiden p.o. und i.v. sowie Regionalanästhesie (inkl. Periduralanästhesie (PDA)). Die Wunde verheilte reizlos, und der Patient konnte wie vor der Operation mobilisiert werden. Der Kostaufbau des Patienten erfolgte zeitgerecht, und eine suffiziente Darmpassage trat ab dem 08.07.2033 ein.
Während des stationären Aufenthalts zeigten sich Blutdruckspitzen von systolisch bis zu 200 mmHg, die durch Anpassung der Antihypertensiva und das Neuansetzen von Bisoprolol 5 mg 1-0-0 erfolgreich behandelt werden konnten. Der Patient wurde am 13.07.2033 entlassen und ist für weitere Nachsorge und Kontrolle angehalten.

User 'Schau dir die zwei Abschnitte "Anamnese und Befund" und "Therapie und Verlauf" noch einmal an: Falls notwendig, passe die Struktur entsprechend an. Achte darauf, dass sich keine Inhalte doppeln.

Assistant Ich habe die beiden Abschnitte noch einmal überprüft und angepasst, um sicherzustellen, dass sich keine Inhalte doppeln. Hier sind die beiden Abschnitte:

****Anamnese und Befund****
Der 65-jährige männliche Patient wurde am 30.06.2033 mit einer akuten Pankreatitis in unsere Klinik aufgenommen. Die Anamnese ergab eine Vortherapie wegen eines Darmkrebses, der am 03/2033 operiert wurde, sowie eine Kolonpolypen-Erstdiagnose am 02/2033. Der Patient litt auch an einer arteriellen Hypertonie und einem Diabetes mellitus seit 02/2032, für den er seit 03/2032 insulinpflichtig ist.

****Therapie und Verlauf****
Der Patient wurde am 04.07.2033 einer explorativen Laparotomie, Pankreaslinksresektion mit Splenektomie und Cholezystektomie unterzogen. Die Operation verlief ohne Komplikationen. Im Anschluss an die Operation erhielt der Patient eine Schmerztherapie mit Opioiden p.o. und i.v. sowie Regionalanästhesie (inkl. Periduralanästhesie (PDA)). Die Wunde verheilte reizlos, und der Patient konnte wie vor der Operation mobilisiert werden. Der Kostaufbau des Patienten erfolgte zeitgerecht, und eine suffiziente Darmpassage trat ab dem 08.07.2033 ein.

	Ich hoffe, dass dies die korrigierten Abschnitte sind!
User	Prüfe, ob die Inhalte den Informationen aus der Patientenakte entsprechen, die ich dir am Anfang gegeben habe. Falls nicht: Korrigiere die Abschnitte entsprechend. Entferne außerdem alle Therapieempfehlungen für Zuhause.
Assistant	Ich habe noch einmal überprüft und festgestellt, dass die Inhalte den Informationen aus der Patientenakte entsprechen. Die Abschnitte sind korrekt und enthalten keine Therapieempfehlungen für Zuhause.

[1]The patient's EHR data is automatically inserted in place of the placeholder.
The bold text marks the generated discharge summary that is to be extracted.

Table 6b: Prompt Chaining Example (Translated to Englsih)

Role	Prompt
System	You are a ward doctor at the university hospital and write discharge summaries for the patient's GP. You use correct medical terminology.
User	Use the following patient data and structure it for a later discharge summary.<br>{data}[1]
Assistant	Here is the discharge summary for the patient's GP:<br>**Discharge letter**<br><br>**Patient data:**<br>* Name: [patient's name]<br>* Gender: male<br>* Height: 175 cm<br>* Body weight: 65 kg<br><br>**Diagnosis:**<br>* Acute pancreatitis<br>* Suspected diagnosis: pancreatic ductal adenocarcinoma (PDAC) / malignant pancreatic tumours, cTNM classification: pT2 pN0<br>* Histological diagnosis: Neuroendocrine tumour (NET)<br>**Previous illnesses:**<br>* Arterial hypertension<br>* Diabetes mellitus since 02/2032, insulin requirement since 03/2032<br>* Colon cancer, operated on 03/2033<br>* Colon polyps, initial diagnosis on 02/2033<br>* Previous abdominal extrapancreatic surgery<br>**Inpatient stay:**<br>* Date of initial diagnosis: 30. 06.2033<br><br>* Date of inpatient admission: 03.07.2033<br>* Date of surgery: 04.07.2033<br>* Date of discharge: 13.07. 2033<br><br>**Operation:**

* Exploratory laparotomy, left pancreatic resection with splenectomy, cholecystectomy
* Surgical access method: Open surgery
* Intraoperative assessment of pancreatic parenchyma/tissue: Texture: Hard, pancreas not lipomatous
* Blood loss of 300 ml, coronary artery severed, antibiotics only administered intraoperatively

Postoperative development:
* Transfer to recovery room after surgery on 04/07/2033
* Intraoperative drainage: Easy-Flow drainage, removed on 10/07/2033
* Analgesics: Opioids p.o., opioids i.v., regional anaesthesia (incl. peridural anaesthesia (PDA))
* Irritation-free wound conditions
* Mobilisation of the patient was possible as before surgery
* Patient's diet was built up in good time
* Sufficient intestinal passage since 08.07.2033

Other abnormalities:
* Blood pressure peaks of systolic up to 200 mmHg, adjustment of antihypertensives and new bisoprolol 5 mg 1-0-0 carried out, the blood pressure peaks were clearly reduced.
Laboratory values:
* Carcinoembryonic antigen (CEA): 4.2 micrograms/L (elevated value)
* Carbohydrate antigen 19-9 (CA 19-9): 8.1 U/ml (value within normal range)
* Total bilirubin: 1.98 mg/dl (elevated value)
* Alkaline phosphatase (AP): 71.0 U/L (value within normal range)
* Gamma-glutamyl transferase (GGT): 33.0 U/L (value within normal range)
* Amylase: 39.0 U/L (value within normal range)
* Lipase: 42. 0 U/L (value in the normal range)

Therapy recommendation:
* Further follow-up and control recommended
Please note that this discharge letter is only an example and must be adapted to the specific needs of the patient and the actual circumstances of the hospitalisation before use.

User Write a section with the heading 'Medical history and findings'. This is part of a discharge letter and should contain all the information that explains why the patient has had the operation. Use the information I have given you.

Assistant Here is the "Medical history and findings" section of the discharge letter:

History and findings
The 65-year-old male patient was admitted to our clinic on 30/06/2033 with acute pancreatitis. The medical history revealed previous treatment for colon cancer, which was operated on 03/2033, as well as a colon polyp initial diagnosis on 02/2033. The patient also suffered from arterial hypertension and diabetes mellitus since 02/2032, for which he has been insulin-dependent since 03/2032.
On admission, acute pancreatitis was found, which was radiologically diagnosed as pancreatic ductal adenocarcinoma (PDAC) / malignant pancreatic tumours, cTNM classification: pT2 pN0. The laboratory values showed an increase in carcinoembryonic antigen (CEA) and bilirubin, while the other values were within the normal range.
The intraoperative examination revealed a neuroendocrine tumour (NET) of the pancreas. The patient underwent exploratory laparotomy, left pancreatic resection with splenectomy and cholecystectomy. The operation was performed without complications and the patient was discharged after a hospitalisation of 10 days.

User Write a section with the heading ‘Therapy and course’. It is part of the discharge letter and contains information about the patient's inpatient treatment. The section should only contain information that is relevant for the GP after the patient has been discharged.

Assistant Here is the "Therapy and course" section of the discharge letter:

Therapy and course
The patient underwent an exploratory laparotomy, left pancreatic resection with splenectomy and cholecystectomy on 04/07/2033. The operation was without complications. Following the operation, the patient received pain therapy with opioids p.o. and i.v. as well as regional anaesthesia (including epidural anaesthesia (PDA)). The wound healed without irritation and the patient could be mobilised as before the operation. The patient's diet was built up in good time and adequate bowel transit was achieved from 8 July 2033.
During the inpatient stay, blood pressure peaks of up to 200 mmHg systolic were observed, which were successfully treated by adjusting the antihypertensive medication and restarting bisoprolol 5 mg 1-0-0. The patient was discharged on 13/07/2033 and is scheduled for further follow-up care and monitoring.

User 'Look at the two sections ‘Medical history and findings’ and ‘Therapy and progression’ again: If necessary, adjust the structure accordingly. Make sure that no content is duplicated.

Assistant I have reviewed and adjusted the two sections again to ensure that no content is duplicated. Here are the two sections:

****History and findings****
The 65-year-old male patient was admitted to our clinic on 30/06/2033 with acute pancreatitis. The medical history revealed previous treatment for colon cancer, which was operated on 03/2033, as well as an initial diagnosis of colon polyps on 02/2033. The patient also suffered from arterial hypertension and diabetes mellitus since 02/2032, for which he has been insulin-dependent since 03/2032.

****Therapy and course****
The patient underwent an exploratory laparotomy, left pancreatic resection with splenectomy and cholecystectomy on 04/07/2033. The operation was without complications. Following the operation, the patient received pain therapy with opioids p.o. and i.v. as well as regional anaesthesia (including epidural anaesthesia (PDA)). The wound healed without irritation and the patient could be mobilised as before the operation. The patient's diet was restored in good time and adequate bowel transit was achieved from 8 July 2033.

I hope that these are the corrected sections!

User Check whether the content corresponds to the information from the patient file that I gave you at the beginning. If not, correct the sections accordingly. Also remove any home therapy recommendations.

Assistant I have checked again and found that the content corresponds to the information in the patient file. The sections are correct and do not contain any therapy recommendations for home use.

---

[1] The patient’s EHR data is automatically inserted in place of the placeholder.
The bold text marks the generated discharge summary that is to be extracted.

EHR Data & Generated Discharge Summaries

The EHR data and corresponding discharge summaries for all 25 cases are presented below. In the discharge summaries, errors are highlighted in red, with explanations provided in the accompanying comments. Pink highlights indicate formulations that are inaccurate but not necessarily incorrect. Blue highlights in the EHR data represent information that was available but omitted from the generated discharge summaries. No judgment was made regarding the relevance of the omitted information to the discharge summaries.

Summary 1

Generated Discharge Summary

Anamnese und Befund:

Die 83-jährige Patientin wurde am 11.05.2034 erstmals in unserer Klinik vorgestellt, nachdem sie eine Bauchspeicheldrüsenzyste aufgewiesen hatte. Die Verdachtsdiagnose lautete auf Intraduktale papillär muzinöse Neoplasien (IPMN) / Zysten, die histologisch nicht gesichert war. Der radiologische Befund zeigte ein duktales Adenokarzinom des Pankreas (PDAC) / maligne Pankreastumoren, der als reszeierbar eingeschätzt wurde. Die Patientin hat eine Vorgeschichte von Arterieller Hypertonie und einer Mamma Ca. rechts, die 2030 operiert wurde, sowie einer Eierstockserkrankung, die 2026 operiert wurde. In der Familienanamnese finden sich Pankreaserkrankungen bei Geschwistern.

Therapie und Verlauf:

Die Patientin wurde am 11.12.2035 stationär aufgenommen und am 14.12.2035 einer roboter-assistierten Pankreaslinksresektion unterzogen. Intraoperativ wurde eine Easy-Flow Drainage und eine Spüldrainage eingelegt. Die intraoperative Bewertung des Pankreasparenchyms/-gewebes zeigte ein nicht lipomatöses Pankreas. Postoperativ zeigten sich reizlose Wundverhältnisse und eine suffiziente Darmpassage ab dem 18.12.2035. Es trat ein Biochemical Leak (Grade A) auf, das jedoch durch die Spüldrainage behandelt werden konnte. Die Patientin erhielt Opioide p.o. als Analgetikum und Metoclopramid i.v. zur Prophylaxe von Übelkeit. Der Kostaufbau des Patienten erfolgte zeitgerecht und die Mobilisation war wie vor der Operation möglich. Die Patientin wurde am 28.12.2035 entlassen.

EHR Data

ALLGEMEINE INFORMATIONEN
Geschlecht: weiblich
Geburtsdatum: 12.02.1952
Körpergröße: 163 cm, Körpergewicht: 69 kg
Bekannte Vorerkrankungen: Arterielle Hypertonie
Vorliegende Veränderungen: Mammabenignom (Erstdiagnose: 09/2028)
Weitere Tumorerkrankungen (nicht am Pankreas):
-Mamma Ca. Rechts: Therapie: OP, Chemotherapie,Bestrahlung, Zeitpunkt: 03/2030
-Eierstöcke: Therapie: OP, Chemotherapie, Zeitpunkt: 11/2026
Vorhergehende abdominelle extrapankreatische OP liegt vor
Familienanamnese: Pankreaserkrankungen liegen vor:
-Pankreaskarzinom: Betroffene Familienmitglieder: Geschwister
Familienanamnese: Extrapankreatische Krebserkrankungen liegen vor:
-Art der Erkrankung: Mamma Ca, Betroffene Familienmitglieder: Kinder, Alter Erkrankungsbeginn: 45
-Art der Erkrankung: Prostatakarzinom, Betroffene Familienmitglieder: Geschwister, Alter Erkrankungsbeginn: 80

-Art der Erkrankung: Pankreaskarzinom, Betroffene Familienmitglieder: Geschwister, Alter Erkrankungsbeginn: 60
-Art der Erkrankung: Blasenkarzinom, Betroffene Familienmitglieder: Geschwister, Alter Erkrankungsbeginn: 81

INFORMATIONEN VOR OPERATION
Grund des Besuchs: Erstvorstellung; Bauchspeicheldrüsenzyste
Datum der Erstdiagnose: 11.05.2034
Aktuelle Verdachtsdiagnose: Intraduktale papillär muzinöse Neoplasien / Zysten
Diagnose histologisch nicht gesichert
Laborwerte: Bilirubin gesamt: 1.16 mg/dl: Wert erhöht
Radiologischer Befund der Verdachtsdiagnose: Duktales Adenokarzinom des Pankreas (PDAC) / maligne Pankreastumoren, Resezierbarkeit des Tumors: Resektabel
Therapieempfehlung: Roboter-assistierte Pankreaslinksresektion

INFORMATIONEN ZUR OPERATION
Datum der stationären Aufnahme: 11.12.2035
OP-Datum: 14.12.2035
Diagnose (gesichert): Intraduktale papillär muzinöse Neoplasien (IPMN) / Zysten
Art der OP: Pankreaslinksresektion, OP-Zugangsmethode: Konvertiert
Intraoperative Drainagen: Easy-Flow Drainage; Spüldrainage: Eingelegt am 14.12.2035, entfernt am 22.12.2035; Salem Drainage
Intraoperative Bewertung des Pankreasparenchyms/-gewebes: Pankreas nicht lipomatös
Sonstige Informationen aus der OP: Transfusion gegeben, Blutverlust von 800 ml, Antibiose nur intraoperativ verabreicht

STATIONÄRER VERLAUF
Verlegung nach OP in Aufwachraum am 14.12.2035, dann zeitnahe Verlegung auf Normalstation
Folgende Analgetika wurden verabreicht: Opioide p.o.
Weitere Medikation: Gabe von Metoclopramid i.v. am 05.10.2023
Pankreas-Fistel: Biochemical Leak (Grade A)
Es zeigten sich reizlose Wundverhältnisse
Mobilisation des Patienten war wie vor Operation möglich
Der Kostaufbau des Patienten erfolgte zeitgerecht
Suffiziente Darmpassage seit 18.12.2035; Erbrechen und Übelkeit an Tag 1-3 postoperativ
Sonstige Auffälligkeiten: im Drainagenpunktat der Hinweis auf eine Pankreasfistel, so dass wir über die einliegende Spüldrainage mit einer Spülung begonnen haben. Im weiteren Verlauf zeigten sich die Enzyme der Drainage rückläufig, sodass die Spülung gestoppt und die Spüldrainagen entfernt werden konnten. Bei weiterem Nachweis von Pankreasenzymen wurden die Easyflow-Drainagen belassen.
Entlassungsdatum: 28.12.2035

Summary 2

Generated Discharge Summary

Anamnese und Befund:

Die 63-jährige Patientin wurde am 08.04.2050 stationär aufgenommen, nachdem sie sich aufgrund einer Verdachtsdiagnose eines lokal fortgeschrittenen dukalen Adenokarzinoms des Pankreas (PDAC) vorstellte. Die Patientin hatte bereits eine Erstdiagnose von Kolonpolypen im Jahr 2043 und eine Brustkrebserkrankung im Jahr 2045, die mit Chemotherapie, Operation und Bestrahlung behandelt wurde. In der Familienanamnese liegen Pankreaserkrankungen vor, insbesondere ein Pankreaskarzinom bei einem Onkel. Die Patientin nahm Kortison als Immunsuppressivum ein, das sie im Rahmen der Chemobegleittherapie erhalten hatte. Vor der Operation zeigte die Patientin Übelkeit und hatte erhöhte Gamma-glutamyl Transferase (GGT)-Werte. Die radiologische Untersuchung ergab ein duktales Adenokarzinom des Pankreas (PDAC) mit borderline Resezierbarkeit.

Therapie und Verlauf:

Die Patientin wurde am 10.04.2050 einer explorativen Laparotomie, Pankreaslinksresektion mittels Splenektomie, vorderer RAMPS sowie Divestment am Truncus coeliacus und an der Arteria mesenterica superior unterzogen. Die Operation verlief ohne Komplikationen, es kam zu einem Blutverlust von 350 ml. Die Patientin wurde nach der Operation in das Aufwachraum verlegt und dann zeitnah auf die Normalstation verlegt. Im Verlauf der stationären Behandlung erhielt die Patientin Opioide p.o. und Regionalanästhesie (inkl. Periduralanästhesie (PDA)) zur Schmerztherapie. Die Wundverhältnisse waren reizlos, die Mobilisation ~~des~~ Patienten war wie vor der Operation möglich. Der Kostaufbau ~~des~~ Patienten erfolgte zeitgerecht, und es zeigte sich eine suffiziente Darmpassage seit dem 16.04.2050. Es kam zu einem Nachweis von Chylusfistel in der Drainage, woraufhin die Patientin für 3 Tage auf eine fettfreie Kost umgestellt wurde. Im Verlauf kam es zu einem deutlichen Rückgang der Chylusfistel. Die Patientin wurde am 22.04.2050 entlassen.

EHR Data

ALLGEMEINE INFORMATIONEN
Geschlecht: weiblich
Geburtsdatum: 14.09.1986
Vorliegende Veränderungen: Kolonpolypen (Erstdiagnose: 05/2043)
Weitere Tumorerkrankungen (nicht am Pankreas):
-Brustkrebs: Therapie: Chemie, OP, Bestrahlung, Zeitpunkt: 10/2045
Familienanamnese: Pankreaserkrankungen liegen vor:
-Pankreaskarzinom: Betroffene Familienmitglieder: Onkel
Familienanamnese: Extrapankreatische Krebserkrankungen liegen vor:
-Art der Erkrankung: Brustkrebs, Betroffene Familienmitglieder: Cousine,
-Art der Erkrankung: Hautkrebs, Betroffene Familienmitglieder: Eltern
Einnahme von Immunsuppressiva: Name: Kortison, Einnahmegrund: Chemobegleittherapie

INFORMATIONEN VOR OPERATION
Grund des Besuchs: Erstvorstellung; Pankreaskarzinom,Bauchspeicheldrüsenzyste
Datum der Erstdiagnose: 06.07.2049
Aktuelle Verdachtsdiagnose: Lokal fortgeschrittenes duktales Adenokarzinom des Pankreas (PDAC)
Diagnose histologisch nicht gesichert
Übelkeit liegt in letzter Zeit vor
Laborwerte: Gamma-glutamyl Transferase (GGT): 58.0 U/L: Wert erhöht
Präoperative Behandlung: Neoadjuvante Therapie: Chemotherapie, Anzahl Zyklen: 14, Vollständigkeit der Chemotherapie: Vollständig, Datum letzte Chemotherapie: 19.03.2050
Radiologischer Befund der Verdachtsdiagnose: Duktales Adenokarzinom des Pankreas (PDAC) / maligne Pankreastumoren, Resezierbarkeit des Tumors: Borderline Resektabel

Therapieempfehlung: Explorative Laparotomie, Pankreaslinksresektion mittels Splenektomie, vordere RAMPS sowie Divestment am Truncus coeliacus sowie an der Arteria mesenterica superior

INFORMATIONEN ZUR OPERATION
Datum der stationären Aufnahme: 08.04.2050
OP-Datum: 10.04.2050
Diagnose (gesichert): borderline Duktales Adenokarzinom des Pankreas (PDAC)
Art der OP: Radikale antegrade modulare Pankreatosplenektomie (RAMPS), OP-Zugangsmethode: Offene OP
Intraoperative Drainagen: Easy-Flow Drainage: Eingelegt am 10.04.2050, entfernt am 19.04.2050; Spüldrainage: Eingelegt am 10.04.2050, entfernt am 14.04.2050
Zusätzliche Gefässsresektion: Divestment A.Hepatica, Divestment Arteria Mesenterica Superior, Divestment Truncus Coeliacus
Intraoperative Bewertung des Pankreasparenchyms/-gewebes: Textur: Weich, Pankreas nicht lipomatös
Sonstige Informationen aus der OP: Blutverlust von 350 ml, Antibiose nur intraoperativ verabreicht

STATIONÄRER VERLAUF
Verlegung nach OP in Aufwachraum am 10.04.2050, dann zeitnahe Verlegung auf Normalstation
Folgende Analgetika wurden verabreicht: Opioide p.o., Regionalanästhesie (inkl. Periduralanästhesie (PDA))
Es zeigten sich reizlose Wundverhältnisse
Mobilisation des Patienten war wie vor Operation möglich
Der Kostaufbau des Patienten erfolgte zeitgerecht
Suffiziente Darmpassage seit 16.04.2050
Sonstige Auffälligkeiten: Nachweis von Chylusfistel in der Drainage erfolgte die Umstellung auf fettfreier Kost für 3 Tage. Im Verlauf kam es zu deutlichem Rückgang der Chylusfistel
Entlassungsdatum: 22.04.2050

Summary 3

Generated Discharge Summary

Anamnese und Befund:
Die 39-jährige Patientin litt seit Mai 2001 an einer chronischen Pankreatitis, die aufgrund einer genetischen Disposition aufgetreten war. Die Patientin klagte über chronische Schmerzen und Verdauungsstörungen mit drei Stuhlgängen pro Tag. Im Labor zeigte sich eine erhöhte Alkalische Phosphatase (AP) von 165,0 U/L. Der radiologische Befund bestätigte die Verdachtsdiagnose einer chronischen Pankreatitis. Aufgrund der Symptomatik und des Befundes wurde eine partielle Duodenopankreatektomie empfohlen.
Therapie und Verlauf:
Die Patientin wurde am 28.06.2038 stationär aufgenommen und am 30.06.2038 einer Duodenumerhaltenden Pankreaskopfresektion (DEPKR) unterzogen. Während der Operation wurden multiple Verkalkungen im Bereich des Pankreaskopfes und -korpus tasten, was die Indikation für eine Whipple-OP ergab. Die Operation wurde jedoch abgebrochen und stattdessen eine offene OP durchgeführt. Intraoperativ wurde ein Easy-Flow Drainage eingelegt, das am 09.07.2038 entfernt wurde. Postoperativ erhielt die Patientin eine antibiotische Therapie mit Pivmecillinam von 04.07.2038 bis 06.07.2038. Zur Schmerztherapie wurden Opioide p.o. und i.v. sowie Regionalanästhesie (inkl. Periduralanästhesie (PDA)) verabreicht. Die Patientin zeigte reizlose Wundverhältnisse und eine verzögerte Mobilisation. Der Kostaufbau erfolgte verzögert und die Patientin litt an Erbrechen und Übelkeit, die jedoch

ab dem dritten Tag postoperativ abnahmen. Die suffiziente Darmpassage trat am 10.07.2038 ein. Die Patientin wurde am 11.07.2038 entlassen.

EHR Data

ALLGEMEINE INFORMATIONEN
Geschlecht: weiblich
Geburtsdatum: 02.06.1999
Körpergröße: 168 cm, Körpergewicht: 90 kg
Familienanamnese: Pankreaserkrankungen liegen vor:
-Chronische Pankreatitis: Betroffene Familienmitglieder: Geschwister
-Pankreaskarzinom: Betroffene Familienmitglieder: Eltern
Familienanamnese: Extrapankreatische Krebserkrankungen liegen vor:
-Art der Erkrankung: Brust, Leukämie, Betroffene Familienmitglieder: Großeltern
Alkoholkonsum: Häufigkeit: Nie

INFORMATIONEN VOR OPERATION
Grund des Besuchs: Chronische Pankreatitis,Bauchspeicheldrüsenzyste
Datum der Erstdiagnose: 25.09.2000
Aktuelle Verdachtsdiagnose: Pankreatitis
Histologische Sicherung nicht notwendig
Chronische Schmerzen liegen vor
Verdauung: Anzahl Stuhlgänge pro Tag: 3
Chronische Pankreatitis liegt vor: seit 05/2001, Ursache: Genetisch
Laborwerte: Alkalische Phosphatase (AP): 165.0 U/L: Wert erhöht
Radiologischer Befund der Verdachtsdiagnose: Chronische Pankreatitis
Therapieempfehlung: Partielle Duodenopankreatektomie

INFORMATIONEN ZUR OPERATION
Datum der stationären Aufnahme: 28.06.2038
OP-Datum: 30.06.2038
Diagnose (gesichert): Pankreatitis
Art der OP: Duodenumerhaltende Pankreaskopfresektion (DEPKR), OP wurde abgebrochen: Grund: Im Bereich des Pankreaskopfes lassen sich multiple Verkalkungen tasten. Ebenso lassen sich im Bereich des Pankreaskorpus multiple Verkalkungen tasten. Jedoch ist der Rest des Pankreas komplett weich. Daher Indikation für Whipple-OP, OP-Zugangsmethode: Offene OP
Intraoperative Drainagen: Easy-Flow Drainage: Eingelegt am 30.06.2038, entfernt am 09.07.2038; Redon wurde zeitgerecht entfernt
Intraoperative Bewertung des Pankreasparenchyms/-gewebes: Textur: Weich, Position: Mittig, Durchmesser: > 3mm, Dicke: Dick, Pankreas nicht lipomatös
Sonstige Informationen aus der OP: Blutverlust von 450 ml, Antibiose nur intraoperativ verabreicht, Besonderheiten: Einlage Redon Drainage

STATIONÄRER VERLAUF
Verlegung nach OP in Aufwachraum am 30.06.2038, dann zeitnahe Verlegung auf Normalstation
Stationäre antibiotische Therapie: Gabe von Pivmecillinam von 04.07.2038 bis 06.07.2038
Folgende Analgetika wurden verabreicht: Opioide p.o., Opioide i.v., Regionalanästhesie (inkl. Periduralanästhesie (PDA))

Weitere Medikation: Gabe von Metoclopramid p.o. von 08.07.2038 bis 10.07.2038; Gabe von Erythromycin i.v. von 07.07.2038 bis 09.07.2038
Es zeigten sich reizlose Wundverhältnisse
Mobilisation des Patienten war wie vor Operation möglich
Der Kostaufbau des Patienten erfolgte verzögert
Suffiziente Darmpassage seit 10.07.2038; Erbrechen und Übelkeit ab Tag 1 postoperativ und über den dritten Tag postoperativ hinaus; postoperative Magensonde vorhanden, postoperative Magensonde wurde entfernt am 08.07.2038; Delayed Gastric Empyting (DGE) A (ISGPS 2007)
Entlassungsdatum: 11.07.2038

Summary 4

Generated Discharge Summary

Anamnese und Befund:
Die 72-jährige Patientin wurde am 17.04.2042 mit Verdachtsdiagnose eines resektablen dukalen Adenokarzinoms des Pankreas (PDAC) vorgestellt. Die Patientin litt unter chronischen Schmerzen im Oberbauch seit 5 Jahren, die täglich auftraten und mit Morphium behandelt wurden. Es bestand ein ungewollter Gewichtsverlust von 3 kg in 12 Wochen. Die Patientin klagte über Verdauungsstörungen mit 3 Stuhlgängen pro Tag und Erbrechen. Ein Ikterus bestand seit 04/2042. Die Endoskopisch retrograde Cholangiopankreatikographie am 21.04.2042 zeigte eine Stenose im Pankreasgang. Die Laborwerte zeigten erhöhte Werte für CEA, CA 19-9, Bilirubin, AP und GGT. Der radiologische Befund bestätigte die Verdachtsdiagnose eines resektablen dukalen Adenokarzinoms des Pankreas.

Therapie und Verlauf:
Die Patientin wurde am 25.05.2042 stationär aufgenommen und am 26.05.2042 einer pyloruserhaltenden Pankreaskopfresektion (ppWhipple) unterzogen. Intraoperativ wurde eine zusätzliche multiviszerale Resektion des Dünndarms durchgeführt. Die Patientin erhielt postoperativ eine antibiotische Therapie mit Ceftriaxon und Metronidazol. Es wurden Opioide und Metoclopramid verabreicht, um die Schmerzen und Übelkeit zu behandeln. Die Patientin zeigte reizlose Wundverhältnisse und eine suffiziente Darmpassage seit 29.05.2042. Es kam zu einem Delayed Gastric Empyting (DGE) A (ISGPS 2007). Am 10. postoperativen Tag kam es zu erneut Ansteigenden Infektwerten ohne klinisches Korrelat. Die Patientin wurde am 09.06.2042 entlassen.

EHR Data

ALLGEMEINE INFORMATIONEN
Geschlecht: weiblich
Geburtsdatum: 03.09.1970
Vorliegende Veränderungen: Kolonpolypen (Erstdiagnose: 05/2031),Uterusmyome
Familienanamnese: Extrapankreatische Krebserkrankungen liegen vor:
-Art der Erkrankung: Brustkrebs triple negativ, Betroffene Familienmitglieder: Kinder

INFORMATIONEN VOR OPERATION
Grund des Besuchs: Erstvorstellung; Pankreaskarzinom,Bauchspeicheldrüsenzyste
Datum der Erstdiagnose: 17.04.2042
Aktuelle Verdachtsdiagnose: Resektables duktales Adenokarzinom des Pankreas (PDAC)
Diagnose histologisch nicht gesichert
Ungewollter Gewichtsverlust: 3 kg in 12 Wochen

Chronische Schmerzen liegen vor: Beschwerdedauer: Seit 5 Jahren, Schmerzcharakter: Chronische Schmerzen, Schmerzverlauf: Persistierend, Schmerzfrequenz: Täglich, Schmerzmedikation: Morphium
Verdauung: Anzahl Stuhlgänge pro Tag: 3, Erbrechen liegt vor
Ikterus liegt vor: seit 04/2042
Endoskopisch retrograde Cholangiopankreatikographie liegt vor: Zeitpunkt: 21.04.2042, Anzahl Untersuchungen bisher: 1, Anzahl eingelegte Stents: 1
Laborwerte: Carcinoembryonales Antigen (CEA): 3.3 mikrogramm/L: Wert erhöht; Carbohydrat-Antigen 19-9 (CA 19-9): 38.1 U/ml: Wert erhöht; Bilirubin gesamt: 1.41 mg/dl: Wert erhöht; Alkalische Phosphatase (AP): 146.0 U/L: Wert erhöht; Gamma-glutamyl Transferase (GGT): 50.0 U/L: Wert erhöht
Radiologischer Befund der Verdachtsdiagnose: Duktales Adenokarzinom des Pankreas (PDAC) / maligne Pankreastumoren, Resezierbarkeit des Tumors: Resektabel
Therapieempfehlung: Whipple OP

INFORMATIONEN ZUR OPERATION
Datum der stationären Aufnahme: 25.05.2042
OP-Datum: 26.05.2042
Diagnose (gesichert): resect. Duktales Adenokarzinom des Pankreas (PDAC)
Art der OP: Pyloruserhaltende Pankreaskopfresektion (ppWhipple), OP-Zugangsmethode: Konvertiert
OP wurde erweitert: Zusätzliche multiviszerale Resektion des Dünndarms durchgeführt
Intraoperative Drainagen: Easy-Flow Drainage: Eingelegt am 26.05.2042, entfernt am 01.06.2042
Intraoperative Bewertung des Pankreasparenchyms/-gewebes: Pankreas nicht lipomatös
Sonstige Informationen aus der OP: Blutverlust von 400 ml, Antibiose weiter postoperativ verabreicht, Besonderheiten: Konversion von daVinci auf offen wegen CO2 Azidose

STATIONÄRER VERLAUF
Verlegung nach OP in Aufwachraum am 26.05.2042, dann zeitnahe Verlegung auf Normalstation
Stationäre antibiotische Therapie: Gabe von Ceftriaxon/Metronidazol i.v. von 25.05.2042 bis 29.05.2042
Folgende Analgetika wurden verabreicht: Opioide p.o., patientenkontrollierte Analgesie
Weitere Medikation: Gabe von Metoclopramid p.o. am 12.10.2023, Gabe von Erythromycin i.v. von 30.05.2042 bis 03.06.2042
Bildgebung: CT Schnittbild:
-Indikation: (Erneuter) Infektwertanstieg, Infektwertanstieg wurde beobachtet
Es zeigten sich reizlose Wundverhältnisse
Mobilisation des Patienten war wie vor Operation möglich
Der Kostaufbau des Patienten erfolgte zeitgerecht
Suffiziente Darmpassage seit 29.05.2042; Erbrechen und Übelkeit ab Tag 1 postoperativ und über den dritten Tag postoperativ hinaus; postoperative Magensonde vorhanden, postoperative Magensonde wurde entfernt am 03.06.2042; Delayed Gastric Empyting (DGE) A (ISGPS 2007)
Sonstige Auffälligkeiten: Nebenbefundlich wurde ein Lymphangiom im Resektat gefunden, welches keinen weiteren Handlungsbedarf erfordert. Am 10. postoperativen Tag kam es zu erneut Ansteigenden Infektwerten ohne klinisches Korrelat
Entlassungsdatum: 09.06.2042

Generated Discharge Summary

Anamnese und Befund:
Die 70-jährige Patientin wurde am 07.08.2028 mit einer Verdachtsdiagnose eines resektable duktales Adenokarzinoms des Pankreas (PDAC) vorgestellt. Die Patientin hatte einen ungewollten Gewichtsverlust von 4 kg in 2 Wochen und Ikterus seit 08/2028. Die Endoskopisch retrograde Cholangiopankreatikographie (ERCP) am 04.09.2028 zeigte eine Stenose des Gallengangs, weshalb ein Gallengangsstent eingelegt wurde. Die Laborwerte zeigten erhöhte Werte für Carbohydrat-Antigen 19-9 (CA 19-9), Bilirubin gesamt, Alkalische Phosphatase (AP) und Gamma-glutamyl Transferase (GGT). Die radiologische Untersuchung bestätigte die Verdachtsdiagnose eines duktales Adenokarzinoms des Pankreas (PDAC).
Therapie und Verlauf:
Die Patientin wurde am 16.09.2028 stationär aufgenommen und am 17.09.2028 einer pylorus erhaltenden Whipple-Operation unterzogen. Intraoperativ wurden Easy-Flow- und Robinson-Drainagen eingelegt, die am 22.09. und 23.09.2028 entfernt wurden. Die intraoperative Bewertung des Pankreasparenchyms/-gewebes zeigte ein nicht lipomatöses Pankreas. Die Patientin verlor 900 ml Blut während der Operation und erhielt postoperativ Antibiotika. Nach der Operation wurde die Patientin in das Aufwachraum verlegt und zeitnah auf die Normalstation verlegt. Die stationäre antibiotische Therapie bestand aus Ceftriaxon/Metronidazol i.v. von 16.09.2028 bis 19.09.2028. Die Schmerztherapie erfolgte mit Opioiden p.o. und Regionalanästhesie (inkl. Periduralanästhesie (PDA)). Weitere Medikation bestand aus Metoclopramid p.o. von 18.09.2028 bis 22.09.2028. Der postoperative Verlauf war komplikationslos, die Wundverhältnisse waren reizlos, die Mobilisation des Patienten war wie vor der Operation möglich und der Kostaufbau erfolgte zeitgerecht. Die suffiziente Darmpassage wurde ab 19.09.2028 beobachtet. Die Patientin wurde am 27.09.2028 entlassen.

EHR Data

ALLGEMEINE INFORMATIONEN
Geschlecht: weiblich
Geburtsdatum: 12.03.1952
Körpergröße: 165 cm, Körpergewicht: 80 kg
Bekannte Vorerkrankungen: Arterielle Hypertonie
Familienanamnese: Extrapankreatische Krebserkrankungen liegen vor:
-Art der Erkrankung: Darmkrebs, Betroffene Familienmitglieder: Eltern

INFORMATIONEN VOR OPERATION
Grund des Besuchs: Erstvorstellung; Bauchspeicheldrüsenzyste
Datum der Erstdiagnose: 07.08.2028
Aktuelle Verdachtsdiagnose: Resektables duktales Adenokarzinom des Pankreas (PDAC)
Diagnose histologisch gesichert
Ungewollter Gewichtsverlust: 4 kg in 2 Wochen
Ikterus liegt vor: seit 08/2028
Endoskopisch retrograde Cholangiopankreatikographie liegt vor: Zeitpunkt: 04.09.2028, Anzahl Untersuchungen bisher: 2, ERCP-Stents eingelegt
Laborwerte: Carbohydrat-Antigen 19-9 (CA 19-9): 41.0 U/ml: Wert erhöht; Bilirubin gesamt: 1.18 mg/dl: Wert erhöht; Alkalische Phosphatase (AP): 145.0 U/L: Wert erhöht; Gamma-glutamyl Transferase (GGT): 294.0 U/L: Wert erhöht
Präoperative Behandlung: Gallengangsstent

Radiologischer Befund der Verdachtsdiagnose: Duktales Adenokarzinom des Pankreas (PDAC) / maligne Pankreastumoren, Resezierbarkeit des Tumors: Resektabel
Therapieempfehlung: Explorative Laparotomie, Pylorus erhaltende Whipple-Operation

INFORMATIONEN ZUR OPERATION
Datum der stationären Aufnahme: 16.09.2028
OP-Datum: 17.09.2028
Diagnose (gesichert): resect. Duktales Adenokarzinom des Pankreas (PDAC)
Art der OP: Pyloruserhaltende Pankreaskopfresektion (ppWhipple), OP-Zugangsmethode: Offene OP
Intraoperative Drainagen: Easy-Flow Drainage: Eingelegt am 17.09.2028, entfernt am 23.09.2028; Robinson Drainage: Eingelegt am 17.09.2028, entfernt am 22.09.2028; Robinson: Eingelegt am 17.09.2028, entfernt am 22.09.2028
Intraoperative Bewertung des Pankreasparenchyms/-gewebes: Pankreas nicht lipomatös
Sonstige Informationen aus der OP: Blutverlust von 900 ml, Antibiose weiter postoperativ verabreicht

STATIONÄRER VERLAUF
Verlegung nach OP in Aufwachraum am 17.09.2028, dann zeitnahe Verlegung auf Normalstation
Stationäre antibiotische Therapie: Gabe von Ceftriaxon/Metronidazol i.v. von 16.09.2028 bis 19.09.2028
Folgende Analgetika wurden verabreicht: Opioide p.o., Regionalanästhesie (inkl. Periduralanästhesie (PDA))
Weitere Medikation: Gabe von Metoclopramid p.o. von 18.09.2028 bis 22.09.2028
Bildgebung: CT Schnittbild:
-Indikation: (Erneuter) Infektwertanstieg, Infektwertanstieg wurde beobachtet
Es zeigten sich reizlose Wundverhältnisse
Mobilisation des Patienten war wie vor Operation möglich
Der Kostaufbau des Patienten erfolgte zeitgerecht
Suffiziente Darmpassage seit 19.09.2028
Entlassungsdatum: 27.09.2028

Summary 6

Generated Discharge Summary

Anamnese und Befund:
Die 39-jährige Patientin wurde aufgrund einer Bauchspeicheldrüsenzyste und eines Verdachts auf einen neuroendokrinen Tumor (NET) in unserer Klinik vorgestellt. Die Patientin litt unter chronischen, leichten, rezidivierenden Schmerzen im Oberbauch, die sie wöchentlich auftraten und mit Morphium behandelt wurden. Zudem war ein ungewollter Gewichtsverlust von 2 kg in 6 Wochen zu verzeichnen. Die Laborwerte zeigten eine erhöhte Lipase von 67,0 U/L und eine verminderte alkalische Phosphatase von 51,0 U/L. Eine Punktion mittels endoskopischen Ultraschalls ergab den histologisch gesicherten Befund eines neuroendokrinen Tumors (NET).
Therapie und Verlauf:
Die Patientin wurde am 10.10.2027 einer roboter-assistierten Duodenopankreatektomie unterzogen, die aufgrund der intraoperativen Befunde erweitert wurde, um eine multiviszerale Resektion des Magens durchzuführen. Die Operation verlief ohne Komplikationen. Im Anschluss an die Operation wurde die Patientin auf die Normalstation verlegt und erhielt eine patientenkontrollierte Analgesie mit Opioiden p.o. sowie Metoclopramid p.o. von 13.10.2027 bis 14.10.2027. Es trat eine Lymphfistel

auf, die durch die Gabe von fettfreier Kost und die Entfernung der Drainage am 20.10.2027 behandelt werden konnte. Die Mobilisation des Patienten war wie vor der Operation möglich, und der Kostaufbau erfolgte zeitgerecht. Die Patientin zeigte suffiziente Darmpassage seit 13.10.2027. Die Entlassung erfolgte am 21.10.2027.

EHR Data

ALLGEMEINE INFORMATIONEN
Geschlecht: weiblich
Geburtsdatum: 08.04.1978
Vorhergehende abdominelle extrapankreatische OP liegt vor
Alkoholkonsum: Häufigkeit: Wöchentlich

INFORMATIONEN VOR OPERATION
Grund des Besuchs: Erstvorstellung; Bauchspeicheldrüsenzyste
Aktuelle Verdachtsdiagnose: Neuroendokriner Tumor (NET)
Diagnose histologisch gesichert: Material: Punktion Endoskopischer Ultraschall, Befund: Neuroendokriner Tumor (NET)
Ungewollter Gewichtsverlust: 2 kg in 6 Wochen
Chronische Schmerzen liegen vor: Schmerzcharakter: Leichte Schmerzen, Schmerzverlauf: Rezidivierend, Schmerzfrequenz: Wöchentlich, Schmerzmedikation: Morphium
Laborwerte: Alkalische Phosphatase (AP): 51.0 U/L: Wert zu niedrig; Lipase: 67.0 U/L: Wert erhöht
Therapieempfehlung: Roboter-assistierte Duodenopankreatektomie

INFORMATIONEN ZUR OPERATION
Datum der stationären Aufnahme: 09.10.2027
OP-Datum: 10.10.2027
Diagnose (gesichert): Neuroendokriner Tumor (NET)
Art der OP: Roboter-assistierte Duodenopankreatektomie, OP-Zugangsmethode: Minimalinvasiver Eingriff (MIC)
OP wurde erweitert: Zusätzliche multiviszerale Resektion des Magens durchgeführt
Intraoperative Drainagen: Robinson Drainage: Eingelegt am 10.10.2027, entfernt am 20.10.2027
Sonstige Informationen aus der OP: Antibiose nur intraoperativ verabreicht, Besonderheiten: Rekonstruktion mittels E-/S-Pankreaticojejunostomie, E-/S-Hepatikojejunostomie, S-/S-Gastrojejunostomie

STATIONÄRER VERLAUF
Verlegung nach OP in Aufwachraum am 10.10.2027, dann zeitnahe Verlegung auf Normalstation
Folgende Analgetika wurden verabreicht: Opioide p.o., patientenkontrollierte Analgesie
Weitere Medikation: Gabe von Metoclopramid p.o. von 13.10.2027 bis 14.10.2027
Weitere Komplikationen: Lymphfistel, Begründung: Triglyceride mehr im Sekret
Es zeigten sich reizlose Wundverhältnisse
Mobilisation des Patienten war wie vor Operation möglich
Der Kostaufbau des Patienten erfolgte zeitgerecht
Suffiziente Darmpassage seit 13.10.2027
Sonstige Auffälligkeiten: Nachweis einer Lymphfistel. Die Patientin erhielt dann für 3 Tage fettfreie Kost, worunter die Drainage aufklarte und keine Triglyceride mehr im Sekret nachweisbar waren. Im Belastungstest zeigte sich dann erneut eine Lymphfistel, bei niedrigen Sekretionsmengen konnte die Drainage jedoch im Verlauf entfernt werden

Entlassungsdatum: 21.10.2027

Summary 7

Generated Discharge Summary

Anamnese und Befund:
Der 43-jährige Patient wurde aufgrund eines Pankreaskarzinoms und einer Bauchspeicheldrüsenzyste in unsere Klinik aufgenommen. Die Erstdiagnose erfolgte am 24.01.2031. Der Patient litt unter einem ungewollten Gewichtsverlust von 15 kg innerhalb von 2 Wochen. Die Laborwerte zeigten eine zu niedrige Amylase von 5,0 U/L. Eine Punktion mittels Endoskopischem Ultraschall ergab einen Befund eines dukalen Adenokarzinoms des Pankreas (PDAC). Der Patient erhielt eine neoadjuvante Chemotherapie über 6 Zyklen, die letzte Chemotherapie fand am 24.03.2031 statt. Der radiologische Befund zeigte ein borderline resektabels duktales Adenokarzinom des Pankreas.

Therapie und Verlauf:
Der Patient wurde am 08.05.2031 stationär aufgenommen und am 09.05.2031 einer Exploration unterzogen. Die Operation musste aufgrund lokaler Inoperabilität abgebrochen werden. Intraoperativ wurde ein Easy-Flow Drainage eingelegt, das am 16.05.2031 entfernt wurde. Die Schnellschnittuntersuchung zeigte Lymphknotenmetastasen. Der Patient erhielt Opioide p.o. als Analgetikum und zeigte reizlose Wundverhältnisse. Die Mobilisation des Patienten war wie vor der Operation möglich. Der Kostaufbau des Patienten erfolgte zeitgerecht und eine suffiziente Darmpassage konnte ab dem 12.05.2031 festgestellt werden. Der Patient wurde am 17.05.2031 entlassen.

EHR Data

ALLGEMEINE INFORMATIONEN
Geschlecht: männlich
Geburtsdatum: 27.08.1978
Diabetes mellitus liegt vor seit 10/2029, Insulinpflicht seit 10/2030, Einnahme orale Antidiabetika seit
Familienanamnese: Extrapankreatische Krebserkrankungen liegen vor:
-Art der Erkrankung: Darmkrebs, Betroffene Familienmitglieder: Eltern, Alter Erkrankungsbeginn: 60

INFORMATIONEN VOR OPERATION
Grund des Besuchs: Pankreaskarzinom,Bauchspeicheldrüsenzyste
Datum der Erstdiagnose: 24.01.2031
Aktuelle Verdachtsdiagnose: Lokal fortgeschrittenes duktales Adenokarzinom des Pankreas (PDAC)
Diagnose histologisch gesichert: Material: Punktion Endoskopischer Ultraschall, Befund: Duktales Adenokarzinom des Pankreas (PDAC)
Ungewollter Gewichtsverlust: 15 kg in 2 Wochen
Laborwerte: Amylase: 5.0 U/L: Wert zu niedrig
Präoperative Behandlung: Neoadjuvante Therapie: Chemotherapie, Anzahl Zyklen: 6, Datum letzte Chemotherapie: 24.03.2031
Radiologischer Befund der Verdachtsdiagnose: Duktales Adenokarzinom des Pankreas (PDAC) / maligne Pankreastumoren, Resezierbarkeit des Tumors: Borderline Resektabel
Therapieempfehlung: Exploration

INFORMATIONEN ZUR OPERATION
Datum der stationären Aufnahme: 08.05.2031
OP-Datum: 09.05.2031

Diagnose (gesichert): borderline Duktales Adenokarzinom des Pankreas (PDAC)
Art der OP: Expl + PE, OP wurde abgebrochen: Grund: Lokale Inoperabilität, OP-Zugangsmethode: Minimalinvasiver Eingriff (MIC)
Intraoperative Drainagen: Easy-Flow Drainage: Eingelegt am 09.05.2031, entfernt am 16.05.2031
Schnellschnitt auffällig: In einer durchgeführten Schnellschnittuntersuchung zeigten sich Lymphknotenmetastasen.
Sonstige Informationen aus der OP: Blutverlust von 200 ml, Antibiose nur intraoperativ verabreicht, Besonderheiten: Aufgrund des sehr ausgedehnten Befundes wurde die Operation nach einer Cholezystektomie beendet.

STATIONÄRER VERLAUF
Verlegung nach OP in Aufwachraum am 09.05.2031, dann zeitnahe Verlegung auf Normalstation
Folgende Analgetika wurden verabreicht: Opioide p.o.
Es zeigten sich reizlose Wundverhältnisse
Mobilisation des Patienten war wie vor Operation möglich
Der Kostaufbau des Patienten erfolgte zeitgerecht
Suffiziente Darmpassage seit 12.05.2031
Entlassungsdatum: 17.05.2031

Summary 8

Generated Discharge Summary

Anamnese und Befund:
Der 42-jährige Patient wurde am 15.04.2046 erstmals vorstellig, weil er unter akuter Pankreatitis und einer Bauchspeicheldrüsenzyste litt. Der Patient berichtete über einen ungewollten Gewichtsverlust von 5 kg innerhalb von 16 Wochen und über chronische Schmerzen seit 16 Wochen, die als leichte, rezidivierende Schmerzen beschrieben wurden. Der Patient gab an, täglich unter Schmerzen zu leiden. Darüber hinaus bestanden Verdauungsprobleme mit einer Stuhlzahl von 1 pro Tag und Erbrechen. Die Laborwerte zeigten erhöhte Werte für Carcinoembryonales Antigen (CEA) und Bilirubin gesamt. Aufgrund dieser Befunde wurde eine explorative Laparotomie empfohlen.

Therapie und Verlauf:
Der Patient wurde am 22.08.2046 stationär aufgenommen und am 23.08.2046 einer Pankreaslinksresektion unterzogen. Intraoperativ wurde eine Robinson-Drainage eingelegt, die am 30.08.2046 entfernt wurde. Die intraoperative Bewertung des Pankreasparenchyms zeigte eine harte Textur und eine dicke Konsistenz. Der Patient wurde nach der Operation in das Aufwachraum verlegt und zeitnah auf die Normalstation verlegt. Während des stationären Aufenthalts erhielt der Patient Analgetika in Form von Opioiden p.o. und i.v. sowie patientenkontrollierte Analgesie. Die Wundverhältnisse waren reizlos, die Mobilisation des Patienten war wie vor der Operation möglich, und der Kostaufbau erfolgte zeitgerecht. Der Patient zeigte suffiziente Darmpassage ab dem 27.08.2046, während Erbrechen und Übelkeit bis zum dritten Tag postoperativ auftraten. Der Patient wurde am 01.09.2046 entlassen.

EHR Data

ALLGEMEINE INFORMATIONEN
Geschlecht: männlich
Geburtsdatum: 13.05.2004
Familienanamnese: Extrapankreatische Krebserkrankungen liegen vor:

-Art der Erkrankung: Schilddrüse, Betroffene Familienmitglieder: Eltern, Alter Erkrankungsbeginn: 55
Alkoholkonsum: Häufigkeit: Wöchentlich

INFORMATIONEN VOR OPERATION
Grund des Besuchs: Erstvorstellung; Akute Pankreatitis,Bauchspeicheldrüsenzyste
Datum der Erstdiagnose: 15.04.2046
Aktuelle Verdachtsdiagnose: Neuroendokriner Tumor (NET)
Diagnose histologisch nicht gesichert
Ungewollter Gewichtsverlust: 5 kg in 16 Wochen
Chronische Schmerzen liegen vor: Beschwerdedauer: Seit 16 Wochen, Schmerzcharakter: Leichte Schmerzen, Schmerzverlauf: Rezidivierend, Schmerzfrequenz: Täglich
Verdauung: Anzahl Stuhlgänge pro Tag: 1, Erbrechen liegt vor
Akute Pankreatitis liegt vor seit 04/2046, Anzahl Schübe: 1, davon wurden 1 im Krankenhaus behandelt
Laborwerte: Carcinoembryonales Antigen (CEA): 2.6 mikrogramm/L: Wert erhöht; Bilirubin gesamt: 1.94 mg/dl: Wert erhöht
Therapieempfehlung: Explorative Laparotomie

INFORMATIONEN ZUR OPERATION
Datum der stationären Aufnahme: 22.08.2046
OP-Datum: 23.08.2046
Diagnose (gesichert): Neuroendokriner Tumor (NET)
Art der OP: Pankreaslinksresektion, OP-Zugangsmethode: Offene OP
Intraoperative Drainagen: Robinson Drainage: Eingelegt am 23.08.2046, entfernt am 30.08.2046
Intraoperative Bewertung des Pankreasparenchyms/-gewebes: Textur: Hart, Dicke: Dick, Pankreas nicht lipomatös
Sonstige Informationen aus der OP: Antibiose nur intraoperativ verabreicht

STATIONÄRER VERLAUF
Verlegung nach OP in Aufwachraum am 23.08.2046, dann zeitnahe Verlegung auf Normalstation
Folgende Analgetika wurden verabreicht: Opioide p.o., Opioide i.v., patientenkontrollierte Analgesie
Es zeigten sich reizlose Wundverhältnisse
Mobilisation des Patienten war wie vor Operation möglich
Der Kostaufbau des Patienten erfolgte zeitgerecht
Suffiziente Darmpassage seit 27.08.2046; Erbrechen und Übelkeit ab Tag 1 postoperativ und über den dritten Tag postoperativ hinaus
Entlassungsdatum: 01.09.2046

Summary 9

Generated Discharge Summary

Anamnese und Befund:
Der 60-jährige Patient wurde aufgrund einer akuten Pankreatitis, eines Pankreaskarzinoms, einer intraduktalen papillär muzinösen Neoplasie (IPMN) und einer Bauchspeicheldrüsenzyste stationär aufgenommen. Die Erstdiagnose erfolgte am 03.04.2045. Der Patient litt unter stark schwankenden Schmerzen im Oberbauch seit 12 Monaten, die täglich auftraten und mit Morphium behandelt wurden. Es bestand ein ungewollter Gewichtsverlust von 30 kg in 24 Wochen. Die Verdauung war beeinträchtigt, mit Durchfall und Erbrechen. Der Patient nahm seit 04/2045 Pankreasenzyme ein. Die Laborwerte zeigten erhöhte Werte für Carbohydrat-Antigen 19-9 (CA 19-9), Alkalische Phosphatase

(AP) und Gamma-glutamyl Transferase (GGT). Eine Endoskopisch retrograde Cholangiopankreatikographie wurde am 12.02.2045 durchgeführt. Der Patient erhielt eine neoadjuvante Chemotherapie mit 6 Zyklen, die am 25.09.2045 abgeschlossen wurde.

Therapie und Verlauf:
Der Patient wurde am 12.10.2045 stationär aufgenommen und am 13.10.2045 einer explorativen Laparotomie und einer totalen Pankreatektomie unterzogen. Die Operation wurde erweitert, um eine multiviszerale Resektion des Magens durchzuführen. Intraoperative Drainagen wurden eingelegt und am 25.10.2045 entfernt. Der Patient erhielt eine antibiotische Therapie und eine Schmerztherapie mit verschiedenen Analgetika. Es kam zu einer Isolierung eines CRP-Anstiegs und reizlosen Wundverhältnissen. Der Patient wurde auf die Intermediate Care Station (IMC) verlegt und erhielt eine parenterale Ernährungstherapie, um eine adäquate Kalorienaufnahme zu gewährleisten. Der Patient wurde am 30.10.2045 entlassen.

EHR Data

ALLGEMEINE INFORMATIONEN
Geschlecht: männlich
Geburtsdatum: 30.07.1985
Bekannte Vorerkrankungen: Arterielle Hypertonie, Koronare Herzkrankheit (KHK)
Diabetes mellitus liegt vor seit 09/2043
Vorliegende Veränderungen: Kolonpolypen (Erstdiagnose: 09/2045)
Raucherstatus: Raucher: Seit 30 Jahren, Zigaretten pro Tag: 10
Alkoholkonsum: Häufigkeit: Nie

INFORMATIONEN VOR OPERATION
Grund des Besuchs: Akute Pankreatitis, Pankreaskarzinom,Intraduktale papillär muzinöse Neoplasien (IPMN),Bauchspeicheldrüsenzyste
Datum der Erstdiagnose: 03.04.2045
Aktuelle Verdachtsdiagnose: Resektables duktales Adenokarzinom des Pankreas (PDAC)
Diagnose histologisch nicht gesichert
Ungewollter Gewichtsverlust: 30 kg in 24 Wochen
Chronische Schmerzen liegen vor: Beschwerdedauer: Seit 12 Monaten, Schmerzcharakter: Stark schwankende Schmerzen, Schmerzverlauf: Rezidivierend, Schmerzfrequenz: Täglich, Schmerzmedikation: Morphium
Verdauung: Anzahl Stuhlgänge pro Tag: 10, Durchfall liegt vor, Erbrechen liegt vor, Einnahme von Pankreasenzymen: seit 04/2045
Akute Pankreatitis liegt vor seit 04/2045
Endoskopisch retrograde Cholangiopankreatikographie liegt vor: Zeitpunkt: 12.02.2045, Anzahl Untersuchungen bisher: 1
Übelkeit liegt in letzter Zeit vor
Laborwerte: Carbohydrat-Antigen 19-9 (CA 19-9): 42.0 U/ml: Wert erhöht; Alkalische Phosphatase (AP): 119.0 U/L: Wert erhöht; Gamma-glutamyl Transferase (GGT): 105.0 U/L: Wert erhöht
Vorhergehende Pankreas-OP: DHC Stent Einlage, Datum der OP: 03.04.2045
Präoperative Behandlung: Gallengangsstent, Neoadjuvante Therapie: Chemotherapie, Anzahl Zyklen: 6, Vollständigkeit der Chemotherapie: Vollständig, Datum letzte Chemotherapie: 25.09.2045
Radiologischer Befund der Verdachtsdiagnose: Duktales Adenokarzinom des Pankreas (PDAC) / maligne Pankreastumoren, Resezierbarkeit des Tumors: Resektabel
Therapieempfehlung: Explorative Laparotomie, Totale Pankreatektomie

INFORMATIONEN ZUR OPERATION
Datum der stationären Aufnahme: 12.10.2045
OP-Datum: 13.10.2045
Diagnose (gesichert): resect. Duktales Adenokarzinom des Pankreas (PDAC)
Art der OP: Totale Pankreatektomie, OP-Zugangsmethode: Offene OP
OP wurde erweitert: Zusätzliche multiviszerale Resektion des Magens durchgeführt
Intraoperative Drainagen: Easy-Flow Drainage: Eingelegt am 13.10.2045, entfernt am 25.10.2045
Zusätzliche Gefäßresektion: Pfortader Interponat: Sonstiges, Divestment Arteria Mesenterica Superior, Divestment Truncus Coeliacus, Arterielle OP: Traingle OP
Sonstige Informationen aus der OP: Transfusion gegeben, Blutverlust von 800 ml, Antibiose weiter postoperativ verabreicht

STATIONÄRER VERLAUF
Verlegung nach OP in Aufwachraum am 13.10.2045, Verlegung auf Intermediate Care Station (IMC)
Stationäre antibiotische Therapie: Gabe von Ceftriaxon/Metronidazol i.v. von 12.10.2045 bis 16.10.2045, Indikation: Perioperative Antibiose; Gabe von Piperacillin/Tazobactam i.v. von 24.10.2045 bis 29.10.2045, Indikation: Verdacht ZVK Infektion
Folgende Analgetika wurden verabreicht: Nicht steroidale Antirheumatika (NSAID), Opioide p.o., Opioide i.v., patientenkontrollierte Analgesie, Regionalanästhesie (inkl. Periduralanästhesie (PDA))
Bildgebung: CT Schnittbild:
-Indikation: (Erneuter) Infektwertanstieg, Behandlung durch Gabe von Antibiotika
Weitere Komplikationen: Isolierter CRP Anstieg
Es zeigten sich reizlose Wundverhältnisse
Mobilisation des Patienten war wie vor Operation möglich
Der Kostaufbau des Patienten erfolgte zeitgerecht
Suffiziente Darmpassage seit 17.10.2045; Erbrechen und Übelkeit ab Tag 1 postoperativ und über den dritten Tag postoperativ hinaus
Sonstige Auffälligkeiten: führten zusätzlich zur Vollkost eine parenterale Ernährungstherapie mit Olimel 1500 ml/d weiter, um eine adäquate Kalorienaufnahme zu gewährleisten und eine post-operative katabole Stoffwechsellage zu vermeiden
Entlassungsdatum: 30.10.2045

Summary 10

Generated Discharge Summary

Anamnese und Befund:
Der 47-jährige Patient wurde aufgrund einer Verdachtsdiagnose eines Neuroendokrinen Tumors (NET) in unserem Haus aufgenommen. Die Erstdiagnose erfolgte am 12.05.2034. Der Patient litt unter einem ungewollten Gewichtsverlust von 7 kg innerhalb von 2 Wochen und einer akuten Pankreatitis seit April 2034, die zweimal aufgetreten ist, wobei ein Schub im Krankenhaus behandelt wurde. Die Laborwerte zeigten eine erhöhte Lipase von 64,0 U/L. Vor der Operation erhielt der Patient eine neoadjuvante Chemotherapie über 4 Zyklen, die am 25.07.2034 abgeschlossen wurde. Die histologische Sicherung der Diagnose erfolgte durch eine Primarius-Probenentnahme.

Therapie und Verlauf:
Der Patient wurde am 24.08.2034 stationär aufgenommen und am 26.08.2034 einer explorativen Laparotomie, einer totalen Pankreatektomie, einer Splenektomie und einer Cholezystektomie unterzogen. Aufgrund lokaler Inoperabilität musste die Operation abgebrochen werden. Es wurden

zusätzliche Leber- und Dünndarmresektionen durchgeführt. Intraoperativ wurde eine Spüldrainage eingelegt, die am 07.09.2034 entfernt wurde. Der Patient erhielt eine Transfusion aufgrund eines Blutverlustes von 1800 ml. Postoperativ wurde der Patient auf die Intermediate Care Station (IMC) verlegt. Es wurden Metoclopramid und Erythromycin verabreicht. Der Patient zeigte reizlose Wundverhältnisse und eine zeitgerechte Mobilisation war möglich. Es traten Erbrechen und Übelkeit nach Tag 3 postoperativ auf. Am zweiten postoperativen Tag wurde eine Ösophago-Gastro-Duodenoskopie durchgeführt, die eine Stauungsgastritis und ein Anastomosen-Ulkus ohne Blutungszeichen zeigte. Daraufhin wurde eine hochdosierte PPI Therapie eingeleitet. Es gab Schwierigkeiten bei der Einstellung der Blutzuckerwerte. Der Patient wurde am 08.09.2034 entlassen.

EHR Data

ALLGEMEINE INFORMATIONEN
Geschlecht: männlich
Geburtsdatum: 11.12.1986
Familienanamnese: Extrapankreatische Krebserkrankungen liegen vor:
-Art der Erkrankung: Hautkrebs / Tumor Gesichtsnerv, Betroffene Familienmitglieder: Eltern, Alter Erkrankungsbeginn: 70

INFORMATIONEN VOR OPERATION
Grund des Besuchs: Erstvorstellung; Pankreaskarzinom,Bauchspeicheldrüsenzyste
Datum der Erstdiagnose: 12.05.2034
Aktuelle Verdachtsdiagnose: Neuroendokriner Tumor (NET)
Diagnose histologisch gesichert: Material: Primarius-Probenentnahme, Befund: Neuroendokriner Tumor (NET)
Ungewollter Gewichtsverlust: 7 kg in 2 Wochen
Akute Pankreatitis liegt vor seit 04/2034, Anzahl Schübe: 2, davon wurden 1 im Krankenhaus behandelt
Laborwerte: Lipase: 64.0 U/L: Wert erhöht
Präoperative Behandlung: Neoadjuvante Therapie: Chemotherapie, Anzahl Zyklen: 4, Vollständigkeit der Chemotherapie: Vollständig, Datum letzte Chemotherapie: 25.07.2034
Therapieempfehlung: Explorative Laparotomie, totale Pankreatektomie, Splenektomie, Cholezystektomie

INFORMATIONEN ZUR OPERATION
Datum der stationären Aufnahme: 24.08.2034
OP-Datum: 26.08.2034
Diagnose (gesichert): Neuroendokriner Tumor (NET)
Art der OP: Pankreaslinksresektion, OP wurde abgebrochen: Grund: Lokale Inoperabilität, OP-Zugangsmethode: Offene OP
OP wurde erweitert: Zusätzliche Leberresektion durchgeführt, Zusätzliche multiviszerale Resektion des Dünndarms durchgeführt
Intraoperative Drainagen: Spüldrainage: Eingelegt am 26.08.2034, entfernt am 07.09.2034
Zusätzliche Gefässsresektion: Divestment A.Hepatica, Divestment Arteria Mesenterica Superior, Divestment Truncus Coeliacus
Sonstige Informationen aus der OP: Transfusion gegeben, Blutverlust von 1800 ml, Antibiose nur intraoperativ verabreicht

STATIONÄRER VERLAUF

Verlegung nach OP in Aufwachraum am 24.08.2034, Verlegung auf Intermediate Care Station (IMC)
Weitere Medikation: Gabe von Metoclopramid i.v. von 03.09.2034 bis 06.09.2034; Gabe von Erythromycin i.v. von 04.09.2034 bis 06.09.2034
Es zeigten sich reizlose Wundverhältnisse
Mobilisation des Patienten war wie vor Operation möglich
Der Kostaufbau des Patienten erfolgte zeitgerecht
Erbrechen und Übelkeit nach Tag 3 postoperativ
Sonstige Auffälligkeiten: am zweiten postoperativen Tag eine Ösophago-Gastro-Duodenoskopie zum Ausschluss einer Magenischämie durchgeführt wurde. Hier zeigte sich eine Stauungsgastritis und ein Anastomosen-Ulkus ohne Blutungszeichen, woraufhin eine hochdosierte PPI Therapie eingeleitet wurde. Schwierigkeiten bei der Einstellung der Blutzuckerwerte
Entlassungsdatum: 08.09.2034

Summary 11

Generated Discharge Summary

Anamnese und Befund:
Der 45-jährige Patient wurde am 20.03.2050 in unser Krankenhaus aufgenommen, nachdem er sich ~~am~~ 03.11.2049 erstmals mit einer akuten Pankreatitis vorgestellt hatte. Die chronische Pankreatitis besteht seit März 2048 und ~~war~~ mehrfach hospitalisiert worden. Der Patient ist seit 20 Jahren Raucher und konsumiert täglich 10 Zigaretten. Im Labor zeigten sich erhöhte Werte für Gamma-Glutamyl-Transferase (GGT), Amylase und Lipase. Der radiologische Befund bestätigte die Verdachtsdiagnose einer chronischen Pankreatitis. Aufgrund der Symptome und Befunde wurde eine explorative Laparotomie und eine pylorusresezierende partielle Pankreatico-Duodenektomie mit Anlage einer Pankreatico-Jejunostomie, Hepatico-Jejunostomie und Belassen der Gastrojejunostomie empfohlen.

Therapie und Verlauf:
Der Patient wurde am 21.03.2050 einer pylorusresezierenden Pankreaskopfresektion (prWhipple) unterzogen. Intraoperativ wurde eine Spüldrainage eingelegt, die am 26.03.2050 entfernt wurde. Der Blutverlust während der Operation betrug 500 ml. Postoperativ erhielt der Patient eine antibiotische Therapie mit Ampillicin/Sulbactam und Piperacillin/Tazobactam. Zur Schmerztherapie wurden Opioide p.o. und i.v., patientenkontrollierte Analgesie und Regionalanästhesie (inkl. Periduralanästhesie (PDA)) eingesetzt. Es kam zu einer Pankreas-Fistel (Biochemical Leak, Grade B) und einer Anlage zweier Drainagen. Der Patient zeigte reizlose Wundverhältnisse und eine suffiziente Darmpassage seit dem 24.03.2050. Am 13. postoperativen Tag trat ein erniedrigter Hämoglobinwert von 7,8 g/dl auf, der durch die Transfusion von zwei Erythrozyten-Konzentraten auf 9 g/dl angehoben werden konnte. Der Patient wurde am 11.04.2050 entlassen.

EHR Data

ALLGEMEINE INFORMATIONEN
Geschlecht: männlich
Geburtsdatum: 05.02.2005
Vorhergehende abdominelle extrapankreatische OP liegt vor
Raucherstatus: Raucher: Seit 20 Jahren, Zigaretten pro Tag: 10
Alkoholkonsum: Häufigkeit: Nie

INFORMATIONEN VOR OPERATION

Grund des Besuchs: Erstvorstellung; Akute Pankreatitis, Chronische Pankreatitis, Bauchspeicheldrüsenzyste
Datum der Erstdiagnose: 03.11.2049
Aktuelle Verdachtsdiagnose: Pankreatitis
Diagnose histologisch nicht gesichert
Chronische Pankreatitis liegt vor: seit 03/2048, Anzahl Hospitalisierungen: Mehrere
Akute Pankreatitis liegt vor seit 03/2048
Laborwerte: Gamma-glutamyl Transferase (GGT): 56.0 U/L: Wert erhöht; Amylase: 71.0 U/L: Wert erhöht; Lipase: 79.0 U/L: Wert erhöht
Radiologischer Befund der Verdachtsdiagnose: Chronische Pankreatitis
Therapieempfehlung: Explorative Laparotomie, Pylorus-resezierende partielle Pankreatico-Duodenektomie mit Anlage einer Pankreatico-Jejunostomie, Hepatico-Jejunostomie und Belassen der Gastrojejunostomie

INFORMATIONEN ZUR OPERATION
Datum der stationären Aufnahme: 20.03.2050
OP-Datum: 21.03.2050
Diagnose (gesichert): Pankreatitis
Art der OP: Pylorusresizierende Pankreaskopfresektion (prWhipple), OP-Zugangsmethode: Offene OP
Intraoperative Drainagen: Spüldrainage: Eingelegt am 21.03.2050, entfernt am 26.03.2050
Intraoperative Bewertung des Pankreasparenchyms/-gewebes: Textur: Weich, Position: Mittig, Durchmesser: > 3mm, Durchmesser in mm: 4, Pankreas nicht lipomatös
Sonstige Informationen aus der OP: Blutverlust von 500 ml, Antibiose nur intraoperativ verabreicht

STATIONÄRER VERLAUF
Verlegung nach OP in Aufwachraum am 20.03.2050, dann zeitnahe Verlegung auf Normalstation
Stationäre antibiotische Therapie: Gabe von Ampillicin/Sulbactam i.v. von 04.04.2050 bis 07.04.2050, Indikation: Deeskalation Piperacillin/Tazobactam; Gabe von Piperacillin/Tazobactam i.v. von 28.03.2050 bis 04.04.2050
Folgende Analgetika wurden verabreicht: Opioide p.o., Opioide i.v., patientenkontrollierte Analgesie, Regionalanästhesie (inkl. Periduralanästhesie (PDA))
Weitere Medikation: Gabe von Erythromycin i.v. von 28.03.2050 bis 31.03.2050
Bildgebung: CT Schnittbild:
-Indikation: (Erneuter) Infektwertanstieg, Behandlung durch Gabe von Antibiotika
-Befund: Sonstige Befunde: Anlage zweier Drainagen
Pankreas-Fistel: Biochemical Leak (Grade B)
Es zeigten sich reizlose Wundverhältnisse
Mobilisation des Patienten war wie vor Operation möglich
Suffiziente Darmpassage seit 24.03.2050; Erbrechen und Übelkeit nach Tag 3 postoperativ; Erneute Anlage einer Magensonde notwendig: Magensonde angelegt am 27.03.2050, entfernt am 02.04.2050
Sonstige Auffälligkeiten: Am 13. postoperativen Tag ist der Patient mit einem erniedrigten Hämoglobinwert von 7,8 g/dl mit sinkender Tendenz aufgefallen, weswegen wir zwei Erythrozyten-Konzentrate transfundierten. Daraufhin stieg der Hämoglobinwert auf 9 g/dl.
Entlassungsdatum: 11.04.2050

Summary 12

Generated Discharge Summary

Anamnese und Befund:
Die 66-jährige Patientin wurde am 07.05.2029 stationär aufgenommen, nachdem sie aufgrund eines Pankreaskarzinoms und einer Bauchspeicheldrüsenzyste in unserer Klinik vorgestellt wurde. Die Patientin litt unter Verdauungsstörungen mit einer Stuhlzahl von 1 pro Tag und Erbrechen. Eine neoadjuvante Chemotherapie wurde über 31 Zyklen durchgeführt, die am 25.01.2028 abgeschlossen wurde. Der radiologische Befund zeigte ein duktales Adenokarzinom des Pankreas (PDAC) mit Zeichen einer akuten Pankreatitis. Aufgrund dieser Befunde wurde eine explorative Laparotomie mit partieller Pankreatico-Duodenektomie, Hepatico-Jejunostomie und Duodeno-Jejunostomie empfohlen.
Therapie und Verlauf:
Die Patientin wurde am 10.05.2029 einer pylorusresizierenden Pankreaskopfresektion (prWhipple) unterzogen. Intraoperativ wurde ein Easy-Flow Drainage eingelegt, das am 16.05.2029 entfernt wurde. Es kam zu einem Blutverlust von 200 ml. Die Patientin wurde postoperativ auf die Normalstation verlegt und erhielt Opioide p.o. und i.v. sowie patientenkontrollierte Analgesie. Die Wundverhältnisse waren reizlos, die Mobilisation des Patienten war wie vor der Operation möglich und der Kostaufbau erfolgte zeitgerecht. Suffiziente Darmpassage trat ab dem 12.05.2029 auf. Die Patientin wurde am 18.05.2029 entlassen.

EHR Data

ALLGEMEINE INFORMATIONEN
Geschlecht: weiblich
Geburtsdatum: 10.06.1956
Körpergröße: 165 cm, Körpergewicht: 73 kg
Bekannte Vorerkrankungen: Arterielle Hypertonie
Vorhergehende abdominelle extrapankreatische OP liegt vor
Raucherstatus: Ehemaliger Raucher: Aufgehört am: 07/1995

INFORMATIONEN VOR OPERATION
Grund des Besuchs: Pankreaskarzinom,Bauchspeicheldrüsenzyste
Datum der Erstdiagnose: 25.05.2027
Aktuelle Verdachtsdiagnose: Resektables duktales Adenokarzinom des Pankreas (PDAC)
Diagnose histologisch nicht gesichert
Verdauung: Anzahl Stuhlgänge pro Tag: 1, Erbrechen liegt vor
Präoperative Behandlung: Neoadjuvante Therapie: Chemotherapie, Anzahl Zyklen: 31, Vollständigkeit der Chemotherapie: Vollständig, Datum letzte Chemotherapie: 25.01.2028
Radiologischer Befund der Verdachtsdiagnose: Duktales Adenokarzinom des Pankreas (PDAC) / maligne Pankreastumoren, Resezierbarkeit des Tumors: Resektabel
Sonstige radiologische Befunde: Zeichen einer akuten Pankreatitis
Therapieempfehlung: Explorative Laparotomie, intraoperative Ultraschall-Untersuchung der Leber, partielle Pankreatico-Duodenektomie mit Anlage einer Pankreatico-Jejunostomie, Hepatico-Jejunostomie und Duodeno-Jejunostomie, Divestment der Arteria mesenterica superior

INFORMATIONEN ZUR OPERATION
Datum der stationären Aufnahme: 07.05.2029
OP-Datum: 10.05.2029
Diagnose (gesichert): resect. Duktales Adenokarzinom des Pankreas (PDAC)
Art der OP: Pylorusresizierende Pankreaskopfresektion (prWhipple), OP-Zugangsmethode: Offene OP
Intraoperative Drainagen: Easy-Flow Drainage: Eingelegt am 10.05.2029, entfernt am 16.05.2029

Zusätzliche Gefässsresektion: Divestment Arteria Mesenterica Superior, Arterielle OP: Traingle OP
Sonstige Informationen aus der OP: Blutverlust von 200 ml, Antibiose nur intraoperativ verabreicht

STATIONÄRER VERLAUF
Verlegung nach OP in Aufwachraum am 10.05.2029, dann zeitnahe Verlegung auf Normalstation
Folgende Analgetika wurden verabreicht: Opioide p.o., Opioide i.v., patientenkontrollierte Analgesie
Es zeigten sich reizlose Wundverhältnisse
Mobilisation des Patienten war wie vor Operation möglich
Der Kostaufbau des Patienten erfolgte zeitgerecht
Suffiziente Darmpassage seit 12.05.2029
Entlassungsdatum: 18.05.2029

Summary 13

Generated Discharge Summary

Anamnese und Befund:
Die 60-jährige Patientin wurde aufgrund eines Verdachts auf ein lokales, fortgeschrittenes duktales Adenokarzinom des Pankreas (PDAC) in unserer Klinik vorgestellt. Die Patientin litt unter chronischen, mittelstarken Schmerzen im Oberbauch seit vier Monaten, die rezidivierend auftraten. Zudem war ein ungewollter Gewichtsverlust von 4 kg in 10 Wochen zu verzeichnen. Die Endoskopisch retrograde Cholangiopankreatikographie (ERCP) vom 16.08.2032 zeigte eine Verdachtsdiagnose auf ein duktales Adenokarzinom des Pankreas (PDAC). Die Laborwerte zeigten erhöhte Werte für Carbohydrat-Antigen 19-9 (CA 19-9), Alkalische Phosphatase (AP) und Gamma-glutamyl Transferase (GGT). Die Patientin erhielt eine neoadjuvante Chemotherapie mit 6 Zyklen, die vollständig durchgeführt wurde.

Therapie und Verlauf:
Die Patientin wurde am 06.12.2032 einer radikalen antegrade modularen Pankreatosplenektomie (RAMPS) unterzogen. Intraoperativ wurde eine zusätzliche multiviszerale Resektion der Nephro durchgeführt. Die OP-Verlauf war komplikationslos, es kam zu einem Blutverlust von 700 ml. Postoperativ erhielt die Patientin eine antibiotische Therapie mit Ampillicin/Sulbactam i.v. von 06.12.2032 bis 09.12.2032. Die Schmerztherapie bestand aus Opioiden p.o. und Regionalanästhesie (inkl. Periduralanästhesie (PDA)). Die Patientin zeigte reizlose Wundverhältnisse und eine suffiziente Darmpassage seit 09.12.2032. Die Mobilisation des Patienten war wie vor der Operation möglich. In einer sonographischen Kontrolle wurde eine Verhaltformation im Oberbauch in der Pankreasloge ohne Septierungen festgestellt, ca. 8 x 4 cm groß. Die Patientin wurde am 22.12.2032 entlassen und zu einer Verlaufskontrolle in 1 Woche wieder eingebestellt.

EHR Data

ALLGEMEINE INFORMATIONEN
Geschlecht: weiblich
Geburtsdatum: 09.05.1972
Familienanamnese: Pankreaserkrankungen liegen vor:
-Pankreaskarzinom
Familienanamnese: Extrapankreatische Krebserkrankungen liegen vor:
-Art der Erkrankung: Brustkrebs, Betroffene Familienmitglieder: Geschwister, Alter Erkrankungsbeginn: 46

INFORMATIONEN VOR OPERATION

Grund des Besuchs: Erstvorstellung; Pankreaskarzinom,Bauchspeicheldrüsenzyste
Datum der Erstdiagnose: 22.07.2032
Aktuelle Verdachtsdiagnose: Lokal fortgeschrittenes duktales Adenokarzinom des Pankreas (PDAC)
Diagnose histologisch gesichert: Befund: Duktales Adenokarzinom des Pankreas (PDAC)
Ungewollter Gewichtsverlust: 4 kg in 10 Wochen
Chronische Schmerzen liegen vor: Beschwerdedauer: Seit 4 Monaten, Schmerzcharakter: Mittelstarke Schmerzen, Schmerzverlauf: Rezidivierend, Schmerzfrequenz: Wöchentlich
Endoskopisch retrograde Cholangiopankreatikographie liegt vor: Zeitpunkt: 16.08.2032, Anzahl Untersuchungen bisher: 1
Laborwerte: Carbohydrat-Antigen 19-9 (CA 19-9): 115.6 U/ml: Wert erhöht; Alkalische Phosphatase (AP): 257.0 U/L: Wert erhöht; Gamma-glutamyl Transferase (GGT): 503.0 U/L: Wert erhöht
Präoperative Behandlung: Neoadjuvante Therapie: Chemotherapie, Anzahl Zyklen: 6, Vollständigkeit der Chemotherapie: Vollständig
Radiologischer Befund der Verdachtsdiagnose: Duktales Adenokarzinom des Pankreas (PDAC) / maligne Pankreastumoren, Resezierbarkeit des Tumors: Lokal fortgeschritten
Therapieempfehlung: Explorative Laparotomie, RAMPS

INFORMATIONEN ZUR OPERATION
Datum der stationären Aufnahme: 05.12.2032
OP-Datum: 06.12.2032
Diagnose (gesichert): Lokal fortgeschrittenes Duktales Adenokarzinom des Pankreas (PDAC)
Art der OP: Radikale antegrade modulare Pankreatosplenektomie (RAMPS), OP-Zugangsmethode: Offene OP
OP wurde erweitert: Zusätzliche multiviszerale Resektion der Nephro durchgeführt
Intraoperative Drainagen: Easy-Flow Drainage: Eingelegt am 06.12.2032, entfernt am 18.12.2032; Spüldrainage: Eingelegt am 06.12.2032, entfernt am 12.12.2032
Zusätzliche Gefässsresektion: Divestment A.Hepatica, Divestment Arteria Mesenterica Superior, Divestment Truncus Coeliacus, Arterielle OP: Traingle OP
Intraoperative Bewertung des Pankreasparenchyms/-gewebes: Textur: Hart, Pankreas nicht lipomatös
Sonstige Informationen aus der OP: Blutverlust von 700 ml, Antibiose weiter postoperativ verabreicht

STATIONÄRER VERLAUF
Verlegung nach OP in Aufwachraum am 06.12.2032, dann zeitnahe Verlegung auf Normalstation
Stationäre antibiotische Therapie: Gabe von Ampillicin/Sulbactam i.v. von 06.12.2032 bis 09.12.2032
Folgende Analgetika wurden verabreicht: Opioide p.o., Regionalanästhesie (inkl. Periduralanästhesie (PDA))
Weitere Medikation: Gabe von Metoclopramid p.o. von 11.12.2032 bis 15.12.2032; Gabe von Erythromycin i.v. am 19.11.2023
Es zeigten sich reizlose Wundverhältnisse
Mobilisation des Patienten war wie vor Operation möglich
Der Kostaufbau des Patienten erfolgte zeitgerecht
Suffiziente Darmpassage seit 09.12.2032; Erbrechen und Übelkeit ab Tag 1 postoperativ und über den dritten Tag postoperativ hinaus
Sonstige Auffälligkeiten: in einer sonographischen Kontrolle eine Verhaltformation im Oberbauch in der Pankreasloge ohne Septierungen, ca. 8 x 4 cm gross. Bei klinisch unauffälliger Patientin und niedrigen Infektparametern entschieden wir uns die Patientin zu entlassen und zu einer Verlaufskontrolle in 1 Woche wieder einzubestellen

Entlassungsdatum: 22.12.2032

Summary 14

Generated Discharge Summary

Anamnese und Befund:
Die 45-jährige Patientin wurde am 05.04.2037 erstmals in unserer Klinik vorgestellt, nachdem sie unter akuten Bauchschmerzen litt. Die Anamnese ergab eine chronische Pankreatitis, die konservativ behandelt wurde. Es bestand der Verdacht auf ein resektables duktales Adenokarzinom des Pankreas (PDAC), was durch radiologische Befunde unterstützt wurde. Die Patientin hatte eine Voroperation wegen einer extrapankreatischen OP. Sie ist ehemaliger Raucher und hat eine arterielle Hypertonie.

Therapie und Verlauf:
Die Patientin wurde am 25.05.2037 stationär aufgenommen und am 26.05.2037 einer explorativen Laparotomie unterzogen. Intraoperativ wurde ein resektables duktales Adenokarzinom des Pankreas (PDAC) diagnostiziert. Die Operation musste jedoch aufgrund einer ausgedehnten Kollateralisierung und Pfortaderthrombose abgebrochen werden. Die Patientin erhielt eine Transfusion und hatte einen Blutverlust von 500 ml. Postoperativ wurde sie auf die Normalstation verlegt und erhielt Analgetika in Form von Opioiden p.o., patientenkontrollierter Analgesie und Regionalanästhesie. Es zeigten sich reizlose Wundverhältnisse und der Kostaufbau des Patienten erfolgte zeitgerecht. Die Patientin litt unter Erbrechen und Übelkeit ab Tag 1 postoperativ und über den dritten Tag postoperativ hinaus. Sie erhielt Metoclopramid i.v. von 29.05.2037 bis 31.05.2037. Die Patientin wurde am 02.06.2037 entlassen.

EHR Data

ALLGEMEINE INFORMATIONEN
Geschlecht: weiblich
Geburtsdatum: 02.04.1992
Körpergröße: 168 cm, Körpergewicht: 48 kg
Bekannte Vorerkrankungen: Arterielle Hypertonie
Vorhergehende abdominelle extrapankreatische OP liegt vor
Raucherstatus: Ehemaliger Raucher: Anzahl Jahre: 28, Zigaretten pro Tag: 8
Alkoholkonsum: Häufigkeit: Nie

INFORMATIONEN VOR OPERATION
Grund des Besuchs: Erstvorstellung; Akute Pankreatitis, Chronische Pankreatitis,Bauchspeicheldrüsenzyste
Datum der Erstdiagnose: 05.04.2037
Aktuelle Verdachtsdiagnose: Resektables duktales Adenokarzinom des Pankreas (PDAC)
Diagnose histologisch nicht gesichert
Chronische Pankreatitis liegt vor: Art der Intervention: Konservativ
Akute Pankreatitis liegt vor
Radiologischer Befund der Verdachtsdiagnose: Duktales Adenokarzinom des Pankreas (PDAC) / maligne Pankreastumoren, Resezierbarkeit des Tumors: Resektabel
Sonstige radiologische Befunde: Zeichen einer akuten Pankreatitis
Therapieempfehlung: Explorative Laparotomie

INFORMATIONEN ZUR OPERATION

Datum der stationären Aufnahme: 25.05.2037
OP-Datum: 26.05.2037
Diagnose (gesichert): resect. Duktales Adenokarzinom des Pankreas (PDAC)
Art der OP: Expl + PE, OP wurde abgebrochen: Grund: ausgedehnter Kollateralisierung und Pfortaderthrombose, OP-Zugangsmethode: Offene OP
Intraoperative Drainagen: Easy-Flow Drainage
Sonstige Informationen aus der OP: Transfusion gegeben, Blutverlust von 500 ml, Antibiose nur intraoperativ verabreicht

STATIONÄRER VERLAUF
Verlegung nach OP in Aufwachraum am 26.05.2037, dann zeitnahe Verlegung auf Normalstation
Folgende Analgetika wurden verabreicht: Opioide p.o., patientenkontrollierte Analgesie, Regionalanästhesie (inkl. Periduralanästhesie (PDA))
Weitere Medikation: Gabe von Metoclopramid i.v. von 29.05.2037 bis 31.05.2037
Es zeigten sich reizlose Wundverhältnisse
Der Kostaufbau des Patienten erfolgte zeitgerecht
Erbrechen und Übelkeit ab Tag 1 postoperativ und über den dritten Tag postoperativ hinaus
Entlassungsdatum: 02.06.2037

Summary 15

Generated Discharge Summary

Anamnese und Befund:
Der 59-jährige Patient wurde aufgrund einer Kontrolle ohne Bauchspeicheldrüsenoperation und einer Bauchspeicheldrüsenzyste in unsere Klinik aufgenommen. Die Verdachtsdiagnose eines Neuroendokrinen Tumors (NET) bestand seit der Erstdiagnose am 08.01.2039. Eine Endoskopisch retrograde Cholangiopankreatikographie (ERCP) wurde am 07.04.2038 durchgeführt. Die Laborwerte zeigten eine erhöhte Gamma-glutamyl Transferase (GGT) von 56.0 U/L. Die Therapieempfehlung lautete auf Pankreasschwanz-Enukleation.

Therapie und Verlauf:
Der Patient wurde am 03.02.2039 stationär aufgenommen und am 04.02.2039 einer minimalinvasiven Enukleation unterzogen. Der Blutverlust während der Operation betrug 100 ml. Antibiotika wurden nur intraoperativ verabreicht. Nach der Operation wurde der Patient in das Aufwachraum verlegt und zeitnah auf die Normalstation verlegt. Die Wundverhältnisse waren reizlos, die Mobilisation des Patienten war wie vor der Operation möglich und der Kostaufbau erfolgte zeitgerecht. Eine suffiziente Darmpassage konnte ab dem 05.02.2039 festgestellt werden. Der Patient wurde am 10.02.2039 entlassen.

EHR Data

ALLGEMEINE INFORMATIONEN
Geschlecht: männlich
Geburtsdatum: 01.12.1983
Körpergröße: 180 cm, Körpergewicht: 126 kg
Bekannte Vorerkrankungen: Arterielle Hypertonie
Diabetes mellitus liegt vor seit 08/2034, Einnahme orale Antidiabetika seit 08/2034
Vorhergehende abdominelle extrapankreatische OP liegt vor

Familienanamnese: Extrapankreatische Krebserkrankungen liegen vor:
-Art der Erkrankung: Brustkrebs, Betroffene Familienmitglieder: Eltern
Raucherstatus: Ehemaliger Raucher: Aufgehört am: 03/2015, Anzahl Jahre: 15, Zigaretten pro Tag: 20

INFORMATIONEN VOR OPERATION
Grund des Besuchs: Kontrolle (ohne Bauchspeicheldrüsenoperation); Bauchspeicheldrüsenzyste
Datum der Erstdiagnose: 08.01.2039
Aktuelle Verdachtsdiagnose: Neuroendokriner Tumor (NET)
Diagnose histologisch nicht gesichert
Endoskopisch retrograde Cholangiopankreatikographie liegt vor: Zeitpunkt: 07.04.2038, Anzahl Untersuchungen bisher: 1
Laborwerte: Gamma-glutamyl Transferase (GGT): 56.0 U/L: Wert erhöht
Therapieempfehlung: Pankreasschwanz-Enukleation

INFORMATIONEN ZUR OPERATION
Datum der stationären Aufnahme: 03.02.2039
OP-Datum: 04.02.2039
Diagnose (gesichert): Neuroendokriner Tumor (NET)
Art der OP: Enukleation, OP-Zugangsmethode: Minimalinvasiver Eingriff (MIC)
Sonstige Informationen aus der OP: Blutverlust von 100 ml, Antibiose nur intraoperativ verabreicht

STATIONÄRER VERLAUF
Verlegung nach OP in Aufwachraum am 03.02.2039, dann zeitnahe Verlegung auf Normalstation
Es zeigten sich reizlose Wundverhältnisse
Mobilisation des Patienten war wie vor Operation möglich
Der Kostaufbau des Patienten erfolgte zeitgerecht
Suffiziente Darmpassage seit 05.02.2039
Entlassungsdatum: 10.02.2039

Summary 16

Generated Discharge Summary

Anamnese und Befund:
Die 40-jährige Patientin wurde am 19.11.2048 erstmals vorstellig, da sie eine Bauchspeicheldrüsenzyste aufwies. Im Rahmen der Diagnostik wurde eine Verdachtsdiagnose auf einen Neuroendokrinen Tumor (NET) am Pankreasschwanz gestellt, die jedoch histologisch nicht gesichert werden konnte. Die Patientin litt unter Verdauungsstörungen mit täglich einem Stuhlgang und Durchfall, zudem trat Übelkeit in letzter Zeit auf. Der Laborwert der Neuronenspezifischen Enolase (NSE) lag mit 23,1 mikrogramm/L erhöht vor. Aufgrund dieser Befunde wurde eine da Vinci-assistierte Enukleation eines NET-Tumors am Pankreasschwanz und eine Cholezystektomie empfohlen.

Therapie und Verlauf:
Die Patientin wurde am 17.08.2049 stationär aufgenommen und am 18.08.2049 einer da Vinci-assistierten Enukleation eines NET-Tumors am Pankreasschwanz unterzogen. Die Operation wurde minimalinvasiv durchgeführt und musste erweitert werden. Der Blutverlust betrug 150 ml, Antibiotika wurden nur intraoperativ verabreicht. Nach der Operation wurde die Patientin in das Aufwachraum verlegt und zeitnah auf die Normalstation überführt. Im Verlauf der stationären Behandlung erhielt die Patientin Analgetika in Form von Opioiden p.o. und patientenkontrollierter Analgesie. Zudem wurde Metoclopramid p.o. von 21.08.2049 bis 22.08.2049 und i.v. am 02.12.2023 verabreicht. Die

Wundverhältnisse waren reizlos, die Mobilisation des Patienten war wie vor der Operation möglich und der Kostaufbau erfolgte zeitgerecht. Die Darmpassage war seit 20.08.2049 suffizient, Erbrechen und Übelkeit traten ab Tag 1 postoperativ und über den dritten Tag postoperativ hinaus auf. Bei erhöhten Blutdrücken wurde die antihypertensive Medikation angepasst, insbesondere die zusätzliche morgendliche Gabe von Candesartan.

EHR Data

ALLGEMEINE INFORMATIONEN
Geschlecht: weiblich
Geburtsdatum: 28.03.2009
Körpergröße: 160 cm, Körpergewicht: 96 kg
Bekannte Vorerkrankungen: Arterielle Hypertonie, FNH, Fettleber
Vorliegende Veränderungen: Kolonpolypen,Ovarialzysten
Weitere Tumorerkrankungen (nicht am Pankreas):
-NFH in der Leber: Therapie: Absetzen der Pille
Vorhergehende abdominelle extrapankreatische OP liegt vor
Familienanamnese: Extrapankreatische Krebserkrankungen liegen vor:
-Art der Erkrankung: Darmkrebs (nicht vererbbar wurde untersucht), Betroffene Familienmitglieder: Eltern, Alter Erkrankungsbeginn: 50
-Art der Erkrankung: Brust, Betroffene Familienmitglieder: Cousine, Alter Erkrankungsbeginn: 40
-Art der Erkrankung: Lunge, Betroffene Familienmitglieder: Onkel,
-Art der Erkrankung: Haut, Betroffene Familienmitglieder: Tante

INFORMATIONEN VOR OPERATION
Grund des Besuchs: Erstvorstellung; Bauchspeicheldrüsenzyste
Datum der Erstdiagnose: 19.11.2048
Aktuelle Verdachtsdiagnose: Neuroendokriner Tumor (NET)
Diagnose histologisch nicht gesichert
Verdauung: Anzahl Stuhlgänge pro Tag: 1, Durchfall liegt vor
Übelkeit liegt in letzter Zeit vor
Laborwerte: Neuronenspezifische Enolase (NSE): 23.1 mikrogramm/L: Wert erhöht
Therapieempfehlung: da Vinci-assistierte Enukleation eines NET-Tumors am Pankreasschwanz, Cholezystektomie

INFORMATIONEN ZUR OPERATION
Datum der stationären Aufnahme: 17.08.2049
OP-Datum: 18.08.2049
Diagnose (gesichert): Neuroendokriner Tumor (NET)
Art der OP: Enukleation, OP-Zugangsmethode: Minimalinvasiver Eingriff (MIC)
OP wurde erweitert
Sonstige Informationen aus der OP: Blutverlust von 150 ml, Antibiose nur intraoperativ verabreicht

STATIONÄRER VERLAUF
Verlegung nach OP in Aufwachraum am 18.08.2049, dann zeitnahe Verlegung auf Normalstation
Folgende Analgetika wurden verabreicht: Opioide p.o., patientenkontrollierte Analgesie
Weitere Medikation: Gabe von Metoclopramid p.o. von 21.08.2049 bis 22.08.2049; Gabe von Metoclopramid i.v. am 02.12.2023

Es zeigten sich reizlose Wundverhältnisse
Mobilisation des Patienten war wie vor Operation möglich
Der Kostaufbau des Patienten erfolgte zeitgerecht
Suffiziente Darmpassage seit 20.08.2049; Erbrechen und Übelkeit ab Tag 1 postoperativ und über den dritten Tag postoperativ hinaus
Sonstige Auffälligkeiten: Bei erhöhten Blutdrücken wurde die antihypertensive Medikation angepasst, insbesondere die zustätzliche morgendliche Gabe von Candesartan. Bei erhöhten Blutdruckwerten wurde bei Unverträglichkeit diverser Blutdruckmedikation eine Einstellung mit 12,5mg HCT begonnen, welche bei Unverträglichkeit nach 1 Tag eingestellt wurde.
Entlassungsdatum: 24.08.2049

Summary 17

Generated Discharge Summary

Anamnese und Befund:
Der 70-jährige Patient wurde am 12.05.2049 erstmals vorstellig mit einer akuten Pankreatitis und Verdacht auf ein Pankreaskarzinom. Der Patient litt unter einem ungewollten Gewichtsverlust von 17 kg in 10 Wochen und zeigte Ikterus seit März 2049. Die Laborwerte zeigten erhöhte Werte für Carbohydrat-Antigen 19-9 (CA 19-9), Gamma-glutamyl Transferase (GGT) und Lipase. Eine radiologische Untersuchung ergab den Verdacht auf ein duktales Adenokarzinom des Pankreas (PDAC) mit lokaler Fortgeschrittenheit. Der Patient erhielt eine neoadjuvante Chemotherapie mit 4 Zyklen, die am 11.08.2049 abgeschlossen wurde. Eine Gallengangsstent wurde implantiert.

Therapie und Verlauf:
Der Patient wurde am 10.04.2050 stationär aufgenommen und am selben Tag einer explorativen Laparotomie unterzogen. Die Operation wurde jedoch aufgrund von Metastasen in der Leber abgebrochen. Intraoperativ wurde eine Robinson-Drainage angelegt und Antibiotika nur intraoperativ verabreicht. Der Patient wurde nach der Operation in den Aufwachraum verlegt und zeitnah auf die Normalstation überführt. Eine stationäre antibiotische Therapie mit Ceftriaxon/Metronidazol wurde von 10.04.2050 bis 17.03.2050 durchgeführt. Der Patient erhielt Opioide p.o. und patientenkontrollierte Analgesie. Die Wundverhältnisse waren reizlos, die Mobilisation des Patienten war wie vor der Operation möglich und der Kostaufbau erfolgte zeitgerecht. Suffiziente Darmpassage trat seit 13.04.2050 auf. Der Patient wurde am 18.04.2050 entlassen.

EHR Data

ALLGEMEINE INFORMATIONEN
Geschlecht: männlich
Geburtsdatum: 23.10.1979
Körpergröße: 174 cm, Körpergewicht: 78 kg
Diabetes mellitus liegt vor seit 03/2049, Insulinpflicht seit 03/2049
Raucherstatus: Raucher: Seit 55 Jahren, Zigaretten pro Tag: 20

INFORMATIONEN VOR OPERATION
Grund des Besuchs: Erstvorstellung; Akute Pankreatitis, Pankreaskarzinom, Bauchspeicheldrüsenzyste
Datum der Erstdiagnose: 12.05.2049
Aktuelle Verdachtsdiagnose: Lokal fortgeschrittenes duktales Adenokarzinom des Pankreas (PDAC)

Diagnose histologisch nicht gesichert
Ungewollter Gewichtsverlust: 17 kg in 10 Wochen
Ikterus liegt vor: seit 03/2049
Laborwerte: Carbohydrat-Antigen 19-9 (CA 19-9): 75.9 U/ml: Wert erhöht; Gamma-glutamyl Transferase (GGT): 69.0 U/L: Wert erhöht; Lipase: 84.0 U/L: Wert erhöht
Präoperative Behandlung: Gallengangsstent, Neoadjuvante Therapie: Chemotherapie, Anzahl Zyklen: 4, Vollständigkeit der Chemotherapie: Vollständig, Datum letzte Chemotherapie: 11.08.2049
Radiologischer Befund der Verdachtsdiagnose: Duktales Adenokarzinom des Pankreas (PDAC) / maligne Pankreastumoren, Resezierbarkeit des Tumors: Lokal fortgeschritten
Therapieempfehlung: explorativen Laparotomie und gegebenenfalls Whipple-Operation, gegebenenfalls mit Gefäßresektion

INFORMATIONEN ZUR OPERATION
Datum der stationären Aufnahme: 10.04.2050
OP-Datum: 10.04.2050
Diagnose (gesichert): Lokal fortgeschrittenes Duktales Adenokarzinom des Pankreas (PDAC)
Art der OP: Exploration und ggf Whipple, OP wurde abgebrochen: Grund: Metastasierung, Lokalisation der Metastasen: Leber; OP-Zugangsmethode: Offene OP
Intraoperative Drainagen: Robinson Drainage
Sonstige Informationen aus der OP: Blutverlust von 50 ml, Antibiose nur intraoperativ verabreicht, Besonderheiten: Bei der Inspektion der Abdominalhöhle fällt eine Infiltration des Leberhilus durch den Tumor auf. . Aufgrund von der Infiltration des Leberhilus ist der Tumor nicht sinnvoll resektabel. Daher kein Fortführen der Pankreasresektion.

STATIONÄRER VERLAUF
Verlegung nach OP in Aufwachraum am 10.04.2050, dann zeitnahe Verlegung auf Normalstation
Stationäre antibiotische Therapie: Gabe von Ceftriaxon/Metronidazol i.v. von 10.04.2050 bis 17.03.2050
Folgende Analgetika wurden verabreicht: Opioide p.o., patientenkontrollierte Analgesie
Es zeigten sich reizlose Wundverhältnisse
Mobilisation des Patienten war wie vor Operation möglich
Der Kostaufbau des Patienten erfolgte zeitgerecht
Suffiziente Darmpassage seit 13.04.2050
Entlassungsdatum: 18.04.2050

Summary 18

Generated Discharge Summary

Anamnese und Befund:
Der 57-jährige Patient wurde am 07.04.2031 mit einer akuten Pankreatitis und einer Bauchspeicheldrüsenzyste vorgestellt. Der Patient litt unter einem ungewollten Gewichtsverlust von 8 kg in 5 Wochen und beschrieb Durchfall und Ikterus seit 04/2031. Die endoskopisch retrograde Cholangiopankreatikographie (ERCP) zeigte eine Stenose, die mit ERCP-Stents behandelt wurde. Die Laborwerte zeigten erhöhte Werte für Carcinoembryonales Antigen (CEA), Carbohydrat-Antigen 19-9 (CA 19-9), Bilirubin gesamt, Alkalische Phosphatase (AP), Gamma-glutamyl Transferase (GGT) und Lipase. Der radiologische Befund bestätigte die Verdachtsdiagnose eines lokal fortgeschrittenen dukalen Adenokarzinoms des Pankreas (PDAC).

Therapie und Verlauf:
Der Patient wurde am 18.05.2031 stationär aufgenommen und am 19.05.2031 einer explorativen Laparotomie unterzogen. Die Operation wurde als Pylorusresizierende Pankreaskopfresektion (prWhipple) durchgeführt, musste jedoch aufgrund von Metastasen in den Lymphknoten abgebrochen werden. Der Patient wurde nach der Operation in den Aufwachraum verlegt und dann auf die Normalstation überführt. Er erhielt eine antibiotische Therapie mit Ceftriaxon/Metronidazol i.v. von 18.05.2031 bis 21.05.2031. Zur Schmerztherapie wurden Nicht steroidale Antirheumatika (NSAID), Opioide p.o. und Regionalanästhesie (inkl. Periduralanästhesie (PDA)) eingesetzt. Der Patient zeigte reizlose Wundverhältnisse und konnte wie vor der Operation mobilisiert werden. Der Kostaufbau des Patienten erfolgte zeitgerecht und die Darmpassage war seit 22.05.2031 suffizient. Der Patient wurde am 24.05.2031 entlassen.

EHR Data

ALLGEMEINE INFORMATIONEN
Geschlecht: männlich
Geburtsdatum: 30.01.1976
Bekannte Vorerkrankungen: Arterielle Hypertonie
Vorhergehende abdominelle extrapankreatische OP liegt vor
Raucherstatus: Raucher: Seit 40 Jahren, Zigaretten pro Tag: 25

INFORMATIONEN VOR OPERATION
Grund des Besuchs: Erstvorstellung; Akute Pankreatitis, Bauchspeicheldrüsenzyste
Datum der Erstdiagnose: 07.04.2031
Aktuelle Verdachtsdiagnose: Lokal fortgeschrittenes duktales Adenokarzinom des Pankreas (PDAC)
Diagnose histologisch gesichert: Material: Probenentnahme operativ, Befund: Duktales Adenokarzinom des Pankreas (PDAC)
Ungewollter Gewichtsverlust: 8 kg in 5 Wochen
Verdauung: Anzahl Stuhlgänge pro Tag: 1, Durchfall liegt vor
Ikterus liegt vor: seit 04/2031
Endoskopisch retrograde Cholangiopankreatikographie liegt vor: ERCP-Stents eingelegt
Laborwerte: Carcinoembryonales Antigen (CEA): 7.6 mikrogramm/L: Wert erhöht; Carbohydrat-Antigen 19-9 (CA 19-9): 278.4 U/ml: Wert erhöht; Bilirubin gesamt: 1.69 mg/dl: Wert erhöht; Alkalische Phosphatase (AP): 344.0 U/L: Wert erhöht; Gamma-glutamyl Transferase (GGT): 112.0 U/L: Wert erhöht; Lipase: 101.0 U/L: Wert erhöht
Radiologischer Befund der Verdachtsdiagnose: Duktales Adenokarzinom des Pankreas (PDAC) / maligne Pankreastumoren, Resezierbarkeit des Tumors: Lokal fortgeschritten
Therapieempfehlung: Explorative Laparotomie

INFORMATIONEN ZUR OPERATION
Datum der stationären Aufnahme: 18.05.2031
OP-Datum: 19.05.2031
Diagnose (gesichert): Lokal fortgeschrittenes Duktales Adenokarzinom des Pankreas (PDAC)
Art der OP: Pylorusresizierende Pankreaskopfresektion (prWhipple), OP wurde abgebrochen: Grund: Metastasierung, Lokalisation der Metastasen: Lymphknoten (fern); OP-Zugangsmethode: Offene OP
Fremdmaterial vorhanden: Magensonde

Schnellschnitt auffällig: Lymphknotenmetastase eines pankreatobiliären Adenokarzinoms, gemäß der morphologischen Befunde passend zu Manifestationen eines klinisch in Betracht gezogenen Pankreaskopfkarzinoms
Sonstige Informationen aus der OP: Antibiose nur intraoperativ verabreicht

STATIONÄRER VERLAUF
Verlegung nach OP in Aufwachraum am 19.05.2031, dann zeitnahe Verlegung auf Normalstation
Stationäre antibiotische Therapie: Gabe von Ceftriaxon/Metronidazol i.v. von 18.05.2031 bis 21.05.2031
Folgende Analgetika wurden verabreicht: Nicht steroidale Antirheumatika (NSAID), Opioide p.o., Regionalanästhesie (inkl. Periduralanästhesie (PDA))
Es zeigten sich reizlose Wundverhältnisse
Mobilisation des Patienten war wie vor Operation möglich
Der Kostaufbau des Patienten erfolgte zeitgerecht
Suffiziente Darmpassage seit 22.05.2031
Sonstige Auffälligkeiten: Aufgrund von positiven aortocavalen Lymphknoten, die in diesem Falle als Fernmetastasen zu werten sind, wurde die Operation nach der Cholezystektomie abgebrochen.
Entlassungsdatum: 24.05.2031

Summary 19

Generated Discharge Summary

Anamnese und Befund:
Die 57-jährige Patientin wurde ~~am~~ 08.03.2039 mit einer akuten Pankreatitis und einer Bauchspeicheldrüsenzyste in unserer Klinik vorgestellt. Die Patientin litt unter Übelkeit, Erbrechen und einem ungewollten Gewichtsverlust von 3 kg innerhalb von 2 Wochen. Ein Ikterus war seit Februar 2039 vorhanden. Die Laborwerte zeigten eine Erhöhung des Bilirubins, der alkalischen Phosphatase, der Gamma-Glutamyl-Transferase, der Amylase und der Lipase. Eine endoskopische retrograde Cholangiopankreatikographie (ERCP) am 14.03.2039 ergab die Einlage eines Gallengangsstents. Der radiologische Befund wies auf ein resektabels duktales Adenokarzinom des Pankreas (PDAC) hin.

Therapie und Verlauf:
Die Patientin wurde am 31.03.2039 stationär aufgenommen und am 01.04.2039 einer explorativen Laparotomie mit Pylorus-resezierender partieller Pankreatikoduodenektomie (Pylorus-resezierender Whipple) unterzogen. Intraoperativ wurde eine Easy-Flow-Drainage und eine Spüldrainage eingelegt, die am 06.04.2039 und 07.04.2039 entfernt wurden. Postoperativ erhielt die Patientin eine antibiotische Therapie mit Ceftriaxon/Metronidazol und Piperacillin/Tazobactam. Es wurden Opioide und Metoclopramid verabreicht. Die Patientin zeigte reizlose Wundverhältnisse und eine suffiziente Darmpassage seit dem 06.04.2039. Es traten Erbrechen und Übelkeit an den Tagen 1-3 postoperativ auf. Eine Bindehaut-Chemosis, die konservativ behandelt wurde, bildete sich vollständig zurück. Die Patientin wurde am 11.04.2039 entlassen.

EHR Data

ALLGEMEINE INFORMATIONEN
Geschlecht: weiblich
Geburtsdatum: 09.06.1981
Alkoholkonsum: Häufigkeit: Täglich

INFORMATIONEN VOR OPERATION
Grund des Besuchs: Erstvorstellung; Akute Pankreatitis,Bauchspeicheldrüsenzyste
Datum der Erstdiagnose: 08.03.2039
Aktuelle Verdachtsdiagnose: Resektables duktales Adenokarzinom des Pankreas (PDAC)
Diagnose histologisch nicht gesichert
Ungewollter Gewichtsverlust: 3 kg in 2 Wochen
Ikterus liegt vor: seit 02/2039
Endoskopisch retrograde Cholangiopankreatikographie liegt vor: Zeitpunkt: 14.03.2039, Anzahl Untersuchungen bisher: 1, ERCP-Stents eingelegt
Übelkeit liegt in letzter Zeit vor
Erbrechen liegt in letzter Zeit vor
Laborwerte: Bilirubin gesamt: 2.51 mg/dl: Wert erhöht; Alkalische Phosphatase (AP): 209.0 U/L: Wert erhöht; Gamma-glutamyl Transferase (GGT): 149.0 U/L: Wert erhöht; Amylase: 309.0 U/L: Wert erhöht; Lipase: 350.0 U/L: Wert erhöht
Präoperative Behandlung: Gallengangsstent
Radiologischer Befund der Verdachtsdiagnose: Duktales Adenokarzinom des Pankreas (PDAC) / maligne Pankreastumoren, Resezierbarkeit des Tumors: Resektabel
Therapieempfehlung: Explorative Laparotomie, Pylorus resezierende, partielle Pankreatikoduodenektomie (Pylorus-resezierende Whipple)

INFORMATIONEN ZUR OPERATION
Datum der stationären Aufnahme: 31.03.2039
OP-Datum: 01.04.2039
Diagnose (gesichert): resect. Duktales Adenokarzinom des Pankreas (PDAC)
Art der OP: Pylorusresizierende Pankreaskopfresektion (prWhipple), OP-Zugangsmethode: Offene OP
Intraoperative Drainagen: Easy-Flow Drainage: Eingelegt am 01.04.2039, entfernt am 06.04.2039; Spüldrainage: Eingelegt am 01.04.2039, entfernt am 07.04.2039
Sonstige Informationen aus der OP: Transfusion gegeben, Blutverlust von 700 ml, Antibiose nur intraoperativ verabreicht, Besonderheiten: Vorliegen aberranter linker Leberarterie aus der Arteria gastrica sinistra

STATIONÄRER VERLAUF
Verlegung nach OP in Aufwachraum am 01.04.2039, dann zeitnahe Verlegung auf Normalstation
Stationäre antibiotische Therapie: Gabe von Ceftriaxon/Metronidazol i.v. von 31.03.2039 bis 05.04.2039, Gabe von Piperacillin/Tazobactam i.v. von 31.03.2039 bis 05.04.2039
Folgende Analgetika wurden verabreicht: Opioide p.o., Opioide i.v.
Weitere Medikation: Gabe von Metoclopramid i.v. am 17.12.2023
Es zeigten sich reizlose Wundverhältnisse
Mobilisation des Patienten war wie vor Operation möglich
Suffiziente Darmpassage seit 06.04.2039; Erbrechen und Übelkeit an Tag 1-3 postoperativ
Sonstige Auffälligkeiten: im Aufwachraum die Transfusion von 2 EKs eingeleitet wurde. Ebenso erfolgte bei niedrigem Albumin-Spiegel eine Humanalbumin-Gabe. . Postoperativ zeigte sich bei der Patientin eine Bindehaut-Chemosis, die nach Rücksprache mit den augenärztlichen Kollegen konservativ behandelt wurde und sich zum 6. Postoperativen Tag vollständig zurückbildete
Entlassungsdatum: 11.04.2039

Summary 20

Generated Discharge Summary

Anamnese und Befund:
Der 43-jährige Patient wurde aufgrund einer chronischen Pankreatitis und einer Bauchspeicheldrüsenzyste in unserer Klinik vorgestellt. Die Erstdiagnose erfolgte am 04.02.2054. Der Patient litt unter stark schwankenden Schmerzen im Oberbauch seit vier Wochen, die täglich auftraten und mit Morphium behandelt wurden. Es bestand ein ungewollter Gewichtsverlust von 3 kg in vier Wochen. Der Patient klagte über Übelkeit und Erbrechen. Die Laborwerte zeigten erhöhte Werte für Carcinoembryonales Antigen (CEA) und Carbohydrat-Antigen 19-9 (CA 19-9). Der radiologische Befund ergab ein duktales Adenokarzinom des Pankreas (PDAC) mit lokaler Fortgeschrittenheit.

Therapie und Verlauf:
Der Patient wurde am 07.03.2054 stationär aufgenommen und am 08.03.2054 einer explorativen Laparotomie unterzogen. Die Operation erfolgte als Pankreaslinksresektion mit minimalinvasivem Eingriff (MIC). Intraoperativ wurde eine Easy-Flow Drainage und eine Spüldrainage eingelegt, die am 21.03.2054 und 30.03.2054 entfernt wurden. Der Patient erhielt eine Transfusion aufgrund eines Blutverlustes von 700 ml. Postoperativ wurde der Patient auf die Normalstation verlegt und erhielt eine antibiotische Therapie mit Pivmecillian 400mg von 25.03.2054 bis 27.03.2054 aufgrund einer unkomplizierten Zystitis. Der Patient erhielt Analgetika in Form von nicht steroidalen Antirheumatika (NSAID), Opioide p.o. und Regionalanästhesie (inkl. Periduralanästhesie (PDA)). Es zeigten sich reizlose Wundverhältnisse, und die Mobilisation des Patienten war wie vor der Operation möglich. Der Patient zeigte eine suffiziente Darmpassage seit 13.03.2054, und Erbrechen und Übelkeit traten ab Tag 1 postoperativ und über den dritten Tag postoperativ hinaus auf. Der Patient wurde am 01.04.2054 entlassen.

EHR Data

ALLGEMEINE INFORMATIONEN
Geschlecht: männlich
Geburtsdatum: 26.07.1980
Körpergröße: 172 cm, Körpergewicht: 83 kg
Bekannte Vorerkrankungen: Arterielle Hypertonie
Vorliegende Veränderungen: Prostatahyperplasie
Weitere Tumorerkrankungen (nicht am Pankreas)
Vorhergehende abdominelle extrapankreatische OP liegt vor
Familienanamnese: Extrapankreatische Krebserkrankungen liegen vor:
-Art der Erkrankung: Leukämie Brustkrebs, Betroffene Familienmitglieder: Geschwister
Raucherstatus: Ehemaliger Raucher: Aufgehört am: 06/2047, Anzahl Jahre: 40, Zigaretten pro Tag: 15
Alkoholkonsum: Häufigkeit: Aufgehört seit: 11/2053, Häufigkeit Konsum vor Aufhören: Gelegentlicher Konsum, Übermäßiger Alkoholkonsum seit 03/2002 bis 11/2053

INFORMATIONEN VOR OPERATION
Grund des Besuchs: Erstvorstellung; Chronische Pankreatitis,Bauchspeicheldrüsenzyste
Datum der Erstdiagnose: 04.02.2054
Aktuelle Verdachtsdiagnose: Lokal fortgeschrittenes duktales Adenokarzinom des Pankreas (PDAC)
Diagnose histologisch nicht gesichert
Ungewollter Gewichtsverlust: 3 kg in 4 Wochen
Chronische Schmerzen liegen vor: Beschwerdedauer: Seit 4 Wochen, Schmerzcharakter: Stark schwankende Schmerzen, Schmerzverlauf: Rezidivierend, Schmerzfrequenz: Täglich, Schmerzmedikation: Morphium
Verdauung: Anzahl Stuhlgänge pro Tag: 1, Erbrechen liegt vor

Akute Pankreatitis liegt vor
Endoskopisch retrograde Cholangiopankreatikographie liegt vor
Übelkeit liegt in letzter Zeit vor
Laborwerte: Carcinoembryonales Antigen (CEA): 7.6 mikrogramm/L: Wert erhöht; Carbohydrat-Antigen 19-9 (CA 19-9): 278.4 U/ml: Wert erhöht
Radiologischer Befund der Verdachtsdiagnose: Duktales Adenokarzinom des Pankreas (PDAC) / maligne Pankreastumoren, Resezierbarkeit des Tumors: Lokal fortgeschritten
Therapieempfehlung: Explorative Laparotomie

INFORMATIONEN ZUR OPERATION
Datum der stationären Aufnahme: 07.03.2054
OP-Datum: 08.03.2054
Diagnose (gesichert): Lokal fortgeschrittenes Duktales Adenokarzinom des Pankreas (PDAC)
Art der OP: Pankreaslinksresektion, OP-Zugangsmethode: Minimalinvasiver Eingriff (MIC)
Intraoperative Drainagen: Easy-Flow Drainage: Eingelegt am 08.03.2054, entfernt am 21.03.2054; Spüldrainage: Eingelegt am 08.03.2054, entfernt am 30.03.2054
Fremdmaterial vorhanden: Magensonde
Intraoperative Bewertung des Pankreasparenchyms/-gewebes: Textur: Hart, Pankreas nicht lipomatös
Sonstige Informationen aus der OP: Transfusion gegeben, Blutverlust von 700 ml, Antibiose nur intraoperativ verabreicht

STATIONÄRER VERLAUF
Verlegung nach OP in Aufwachraum am 08.03.2054, dann zeitnahe Verlegung auf Normalstation
Stationäre antibiotische Therapie: Gabe von Pivmecillian 400mg von 25.03.2054 bis 27.03.2054, Indikation: Unkomplizierte Zystitis
Folgende Analgetika wurden verabreicht: Nicht steroidale Antirheumatika (NSAID), Opioide p.o., Regionalanästhesie (inkl. Periduralanästhesie (PDA))
Weitere Medikation: Gabe von Metoclopramid p.o. von 13.03.2054 bis 27.03.2054
Es zeigten sich reizlose Wundverhältnisse
Mobilisation des Patienten war wie vor Operation möglich
Suffiziente Darmpassage seit 13.03.2054; Erbrechen und Übelkeit ab Tag 1 postoperativ und über den dritten Tag postoperativ hinaus
Entlassungsdatum: 01.04.2054

Summary 21

Generated Discharge Summary

Anamnese und Befund:
Die 57-jährige Patientin wurde aufgrund einer Bauchspeicheldrüsenzyste und Verdachts auf einen Neuroendokrinen Tumor (NET) in unserer Klinik vorgestellt. Die histologische Sicherung erfolgte durch eine Punktion mittels Endoskopischen Ultraschalls, die einen NET bestätigte. Die Patientin litt unter Verdauungsstörungen mit zwei Stuhlgängen pro Tag und Erbrechen. Die Laborwerte zeigten eine verminderte Amylase- und Lipase-Aktivität. Der radiologische Befund ergab ein duktales Adenokarzinom des Pankreas (PDAC) mit einer respektablen Tumorgröße.

Therapie und Verlauf:

Die Patientin wurde am 26.06.2033 stationär aufgenommen und am 27.06.2033 einer totalen Pankreatektomie unterzogen. Intraoperativ wurde eine multiviszerale Resektion des Kolons durchgeführt. Postoperativ wurde die Patientin auf die Intermediate Care Station (IMC) verlegt und erhielt eine antibiotische Therapie wegen einer hospital acquired Pneumonie. Es wurden Opioide und Metoclopramid verabreicht. Die Patientin zeigte reizlose Wundverhältnisse und eine suffiziente Darmpassage. Es trat eine Pneumonie auf, die antibiotisch behandelt wurde. Aufgrund eines Delayed Gastric Emptying (DGE) A (ISGPS 2007) wurde eine Magensonde angelegt. Die Patientin erhielt Blutzucker- und Diabetesschulungen durch spezialisiertes Personal. Die Entlassung erfolgte am 15.07.2033.

EHR Data

ALLGEMEINE INFORMATIONEN
Geschlecht: weiblich
Geburtsdatum: 22.11.1975
Vorhergehende abdominelle extrapankreatische OP liegt vor
Raucherstatus: Ehemaliger Raucher
Alkoholkonsum: Häufigkeit: Nie

INFORMATIONEN VOR OPERATION
Grund des Besuchs: Erstvorstellung; Bauchspeicheldrüsenzyste
Aktuelle Verdachtsdiagnose: Neuroendokriner Tumor (NET)
Diagnose histologisch gesichert: Material: Punktion Endoskopischer Ultraschall, Befund: Neuroendokriner Tumor (NET)
Verdauung: Anzahl Stuhlgänge pro Tag: 2, Erbrechen liegt vor
Laborwerte: Amylase: 4.0 U/L: Wert zu niedrig; Lipase: 18.0 U/L: Wert zu niedrig
Radiologischer Befund der Verdachtsdiagnose: Duktales Adenokarzinom des Pankreas (PDAC) / maligne Pankreastumoren, Resezierbarkeit des Tumors: Resektabel
Therapieempfehlung: Pankreaskopfresektion; gegebenenfalls intraoperativ die Entscheidung zu einer totalen Pankreatektomie

INFORMATIONEN ZUR OPERATION
Datum der stationären Aufnahme: 26.06.2033
OP-Datum: 27.06.2033
Diagnose (gesichert): Neuroendokriner Tumor (NET)
Art der OP: Totale Pankreatektomie, OP-Zugangsmethode: Minimalinvasiver Eingriff (MIC)
OP wurde erweitert: Zusätzliche multiviszerale Resektion des Kolons durchgeführt
Intraoperative Drainagen: Robinson Drainage: Eingelegt am 27.06.2033, entfernt am 05.07.2033
Intraoperative Bewertung des Pankreasparenchyms/-gewebes: Durchmesser: <= 3mm, Dicke: Dick, Pankreas lipomatös
Sonstige Informationen aus der OP: Blutverlust von 0 ml, Antibiose nur intraoperativ verabreicht, Besonderheiten: Umentscheidung von Pankreaskopfresektion zu Totaler Pankreatektomie

STATIONÄRER VERLAUF
Verlegung nach OP in Aufwachraum am 27.06.2033, Verlegung auf Intermediate Care Station (IMC)
Stationäre antibiotische Therapie: Gabe von Piperacillin/Tazobactam i.v. von 30.06.2033 bis 06.07.2033, Indikation: Hospital aquired pneumonia
Folgende Analgetika wurden verabreicht: Opioide p.o., patientenkontrollierte Analgesie
Weitere Medikation: Gabe von Metoclopramid i.v. von 07.07.2033 bis 13.07.2033

Bildgebung: CT Schnittbild:
-Indikation: (Erneuter) Infektwertanstieg, Behandlung durch Gabe von Antibiotika
-Befund: Sonstige Befunde: linksseitige Unterlappenatelektase mit möglicherweise Infiltration
Weitere Komplikationen: Pneumonie
Es zeigten sich reizlose Wundverhältnisse
Mobilisation des Patienten war wie vor Operation möglich
Der Kostaufbau des Patienten erfolgte zeitgerecht
Suffiziente Darmpassage seit 28.06.2033; Erbrechen und Übelkeit ab Tag 1 postoperativ und über den dritten Tag postoperativ hinaus; Erneute Anlage einer Magensonde notwendig: Delayed Gastric Empyting (DGE) A (ISGPS 2007)
Sonstige Auffälligkeiten: Es erfolgten Blutzucker- und Diabetesschulungen durch spezialisiertes Personal. Eine suffiziente Umsetzung durch die Patientin konnte bei teilweise stark schwankenden Blutzuckerspiegeln noch nicht erreicht werden, sodass hier eine regelmässige Kontrolle und Einstellung durch das Personal weiterhin nötig war
Entlassungsdatum: 15.07.2033

Summary 22

Generated Discharge Summary

Anamnese und Befund:
Die 55-jährige Patientin wurde aufgrund einer Verdachtsdiagnose eines oligometastatischen dukalen Adenokarzinoms des Pankreas (PDAC) in unserer Klinik aufgenommen. Die Patientin hatte bereits eine Erstdiagnose von Kolonpolypen und eine positive Familienanamnese für Pankreaserkrankungen und extrapankreatische Krebserkrankungen. Sie litt unter ungewolltem Gewichtsverlust, chronischen Schmerzen, Verdauungsstörungen, Übelkeit und Erbrechen. Die Laborwerte zeigten erhöhte Werte für Carcinoembryonales Antigen (CEA), Carbohydrat-Antigen 19-9 (CA 19-9), Alkalische Phosphatase (AP), Gamma-glutamyl Transferase (GGT) und Lipase. Die Patientin hatte bereits eine neoadjuvante Chemotherapie erhalten, die jedoch abgebrochen wurde.

Therapie und Verlauf:
Die Patientin wurde am 13.08.2041 einer explorativen Laparotomie unterzogen, die jedoch aufgrund von Metastasen abgebrochen wurde. Im Anschluss an die Operation wurde die Patientin auf die Normalstation verlegt und erhielt Analgetika in Form von Opioiden p.o. und Regionalanästhesie. Es zeigten sich reizlose Wundverhältnisse, und der Kostaufbau des Patienten erfolgte zeitgerecht. Suffiziente Darmpassage trat ab dem 16.08.2041 auf. Unter analgetischer Therapie mit einliegendem Periduralkatheter trat eine ausgeprägte Hypotonie auf, die jedoch nach Entfernung des Katheters und Umstellung auf orale Medikation mit Hydromorphon und Novalgin beherrscht werden konnte. Der Kreislaufzustand der Patientin stabilisierte sich zunehmend. Die Patientin wurde am 18.08.2041 entlassen.

EHR Data

ALLGEMEINE INFORMATIONEN
Geschlecht: weiblich
Geburtsdatum: 13.08.1986
Vorliegende Veränderungen: Kolonpolypen (Erstdiagnose: 10/2040)
Familienanamnese: Pankreaserkrankungen liegen vor:
-Pankreaskarzinom: Betroffene Familienmitglieder: Eltern

Familienanamnese: Extrapankreatische Krebserkrankungen liegen vor:
-Art der Erkrankung: Darmkrebs, Betroffene Familienmitglieder: Eltern
Raucherstatus: Raucher: Seit 30 Jahren, Zigaretten pro Tag: 15

INFORMATIONEN VOR OPERATION
Grund des Besuchs: Erstvorstellung; Pankreaskarzinom,Bauchspeicheldrüsenzyste
Datum der Erstdiagnose: 24.01.2041
Aktuelle Verdachtsdiagnose: Oligomet. duktales Adenokarzinom des Pankreas (PDAC)
Diagnose histologisch gesichert
Ungewollter Gewichtsverlust: 18 kg in 10 Wochen
Chronische Schmerzen liegen vor: Schmerzcharakter: Mittelstarke Schmerzen, Schmerzverlauf: Rezidivierend, Schmerzfrequenz: Täglich, Schmerzmedikation: Morphium
Verdauung: Anzahl Stuhlgänge pro Tag: 2, Durchfall liegt vor, Einnahme von Pankreasenzymen: seit 05/2041
Übelkeit liegt in letzter Zeit vor
Erbrechen liegt in letzter Zeit vor
Laborwerte: Carcinoembryonales Antigen (CEA): 5.2 mikrogramm/L: Wert erhöht; Carbohydrat-Antigen 19-9 (CA 19-9): 103.9 U/ml: Wert erhöht; Alkalische Phosphatase (AP): 134.0 U/L: Wert erhöht; Gamma-glutamyl Transferase (GGT): 61.0 U/L: Wert erhöht; Lipase: 19.0 U/L: None
Präoperative Behandlung: Neoadjuvante Therapie: Chemotherapie, Anzahl Zyklen: 4, Vollständigkeit der Chemotherapie: Abgebrochen
Radiologischer Befund der Verdachtsdiagnose: Duktales Adenokarzinom des Pankreas (PDAC) / maligne Pankreastumoren, Resezierbarkeit des Tumors: Metastasiert
Therapieempfehlung: Explorative Laparotomie

INFORMATIONEN ZUR OPERATION
Datum der stationären Aufnahme: 12.08.2041
OP-Datum: 13.08.2041
Diagnose (gesichert): oligomet. Duktales Adenokarzinom des Pankreas (PDAC)
Art der OP: Expl + PE, OP wurde abgebrochen: Grund: Metastasierung, Lokalisation der Metastasen:
OP-Zugangsmethode: Offene OP
Intraoperative Bewertung des Pankreasparenchyms/-gewebes: Textur: Hart, Pankreas nicht lipomatös
Schnellschnitt auffällig: im Bereich von der Leber-PE Segment IV: spärlicher Nachweis von residuellen Adenokarzinomzellen, ebenso Nachweis von spärlich residualen Adenokarzinomzellen im Bereich der Probeexzision links parailiakal und am Abgang der Arteria mesenterica inferior, im Bereich von der Probeexzision von Lebersegment VII zeigen sich atypische Zellen, hier jedoch letztlich nicht klar, ob es sich ebenfalls um residülle Tumormanifestationen handelt oder nicht
Sonstige Informationen aus der OP: Antibiose nur intraoperativ verabreicht

STATIONÄRER VERLAUF
Verlegung nach OP in Aufwachraum am 12.08.2041, dann zeitnahe Verlegung auf Normalstation
Folgende Analgetika wurden verabreicht: Opioide p.o., Regionalanästhesie (inkl. Periduralanästhesie (PDA))
Es zeigten sich reizlose Wundverhältnisse
Der Kostaufbau des Patienten erfolgte zeitgerecht
Suffiziente Darmpassage seit 16.08.2041
Sonstige Auffälligkeiten: Unter analgetischer Therapie mit einliegendem Periduralkatheter bot die Patientin eine ausgeprägte Hypotonie. Der Katheter konnte dann entfernt werden und auf eine orale

Medikation mit Hydromorphon und Novalgin umgestellt werden. Der Kreislaufzustand der Patientin stabilisierte sich zunehmend.
Entlassungsdatum: 18.08.2041

Summary 23

Generated Discharge Summary

Anamnese und Befund:
Der 46-jährige Patient wurde am 13.02.2033 erstmals in unserer Klinik vorgestellt, nachdem er über chronische Schmerzen im Oberbauch und Übelkeit geklagt hatte. Der Patient hatte in den letzten 14 Wochen einen ungewollten Gewichtsverlust von 15 kg erlitten. Die Laborwerte zeigten erhöhte Werte für Carcinoembryonales Antigen (CEA), Bilirubin, Alkalische Phosphatase (AP), Gamma-glutamyl Transferase (GGT), Amylase und Lipase. Die Endoskopisch retrograde Cholangiopankreatikographie (ERCP) vom 27.01.2021 zeigte eine Gallengangsobstruktion, die durch einen Gallengangsstent behandelt wurde. Der radiologische Befund wies auf ein duktales Adenokarzinom des Pankreas (PDAC) hin, das als reszeierbar eingeschätzt wurde.

Therapie und Verlauf:
Der Patient wurde am 05.03.2033 stationär aufgenommen und am 06.03.2033 einer explorativen Laparotomie unterzogen. Die Operation wurde aufgrund von Metastasen in der Leber abgebrochen. Intraoperativ wurde eine Robinson-Drainage angelegt, die zeitgerecht entfernt wurde. Der Patient wurde nach der Operation in das Aufwachraum verlegt und dann auf die Normalstation überführt. Die postoperative Schmerztherapie erfolgte mit Opioiden p.o. und patientenkontrollierter Analgesie. Die Wundverhältnisse waren reizlos, die Mobilisation des Patienten war wie vor der Operation möglich. Der Kostaufbau des Patienten erfolgte zeitgerecht und die Darmpassage war seit dem 11.03.2033 suffizient. Der Patient wurde am 12.03.2033 entlassen.

EHR Data

ALLGEMEINE INFORMATIONEN
Geschlecht: männlich
Geburtsdatum: 04.08.1976
Körpergröße: 180 cm, Körpergewicht: 112 kg
Bekannte Vorerkrankungen: Arterielle Hypertonie
Diabetes mellitus liegt vor Insulinpflicht Einnahme orale Antidiabetika
Raucherstatus: Ehemaliger Raucher

INFORMATIONEN VOR OPERATION
Grund des Besuchs: Erstvorstellung; Akute Pankreatitis, Bauchspeicheldrüsenzyste
Datum der Erstdiagnose: 13.02.2033
Aktuelle Verdachtsdiagnose: Oligomet. duktales Adenokarzinom des Pankreas (PDAC)
Diagnose histologisch nicht gesichert
Ungewollter Gewichtsverlust: 15 kg in 14 Wochen
Chronische Schmerzen liegen vor: Beschwerdedauer: Seit 2 Wochen, Schmerzcharakter: Leichte Schmerzen, Schmerzverlauf: Rezidivierend, Schmerzfrequenz: Täglich, Schmerzmedikation: Morphium
Akute Pankreatitis liegt vor
Ikterus liegt vor

Endoskopisch retrograde Cholangiopankreatikographie liegt vor: Zeitpunkt: 27.01.2021, Anzahl Untersuchungen bisher: 1, ERCP-Stents eingelegt
Übelkeit liegt in letzter Zeit vor
Erbrechen liegt in letzter Zeit vor
Laborwerte: Carcinoembryonales Antigen (CEA): 4.2 mikrogramm/L: Wert erhöht; Bilirubin gesamt: 1.98 mg/dl: Wert erhöht; Alkalische Phosphatase (AP): 110.0 U/L: Wert erhöht; Gamma-glutamyl Transferase (GGT): 230.0 U/L: Wert erhöht; Amylase: 92.0 U/L: Wert erhöht; Lipase: 258.0 U/L: Wert erhöht
Präoperative Behandlung: Gallengangsstent
Präoperative Gallengangsdrainage gelegt: Art der Drainage: endoskopisch (Metallstent)
Radiologischer Befund der Verdachtsdiagnose: Duktales Adenokarzinom des Pankreas (PDAC) / maligne Pankreastumoren, Resezierbarkeit des Tumors: Resektabel
Sonstige radiologische Befunde: Zeichen einer akuten Pankreatitis
Therapieempfehlung: Explorative Laparotomie, 2-fache Probenentnahme der Leber, intraoperativer Ultraschall der Leber

INFORMATIONEN ZUR OPERATION
Datum der stationären Aufnahme: 05.03.2033
OP-Datum: 06.03.2033
Diagnose (gesichert): oligomet. Duktales Adenokarzinom des Pankreas (PDAC)
Art der OP: Expl + PE, OP wurde abgebrochen: Grund: Metastasierung, Lokalisation der Metastasen: Leber; OP-Zugangsmethode: Offene OP
Intraoperative Drainagen: Robinson Drainage wurde zeitgerecht entfernt
Sonstige Informationen aus der OP: Antibiose nur intraoperativ verabreicht

STATIONÄRER VERLAUF
Verlegung nach OP in Aufwachraum am 06.03.2033, dann zeitnahe Verlegung auf Normalstation
Folgende Analgetika wurden verabreicht: Opioide p.o., patientenkontrollierte Analgesie
Es zeigten sich reizlose Wundverhältnisse
Mobilisation des Patienten war wie vor Operation möglich
Der Kostaufbau des Patienten erfolgte zeitgerecht
Suffiziente Darmpassage seit 11.03.2033
Entlassungsdatum: 12.03.2033

Summary 24

Generated Discharge Summary

Anamnese und Befund:
Der 65-jährige Patient wurde ~~am~~ 23.08.2038 mit einer akuten Pankreatitis und einer Bauchspeicheldrüsenzyste in unsere Klinik aufgenommen. Die Verdachtsdiagnose eines Neuroendokrinen Tumors (NET) wurde aufgrund erhöhter Laborwerte (CA 19-9, Chromogranin A) und eines radiologischen Befundes eines ~~duk~~alen Adenokarzinoms des Pankreas (PDAC) gestellt. Der Patient hat eine bekannte Arterielle Hypertonie und einen Diabetes mellitus seit 04/2037, für den er insulinpflichtig ist. Zudem liegen Kolonpolypen und ein Darmkrebs vor, der bereits operativ behandelt wurde.

Therapie und Verlauf:
Der Patient wurde am 27.08.2038 einer explorativen Laparotomie, Pankreaslinksresektion mit Splenektomie und Cholezystektomie unterzogen. Intraoperativ wurde ein Easy-Flow Drainage

eingelegt, das am 02.09.2038 entfernt wurde. Der Blutverlust betrug 300 ml, und Antibiotika wurden nur intraoperativ verabreicht. Postoperativ wurde der Patient auf die Normalstation verlegt und erhielt Analgetika in Form von Opioiden p.o. und i.v. sowie Regionalanästhesie. Die Wundverhältnisse waren reizlos, und die Mobilisation des Patienten war wie vor der Operation möglich. Der Kostaufbau erfolgte zeitgerecht, und eine suffiziente Darmpassage trat ab dem 31.08.2038 auf. Es zeigten sich Blutdruckspitzen von systolisch bis zu 200 mmHg, die durch Anpassung der Antihypertensiva und das Neuansetzen von Bisoprolol 5 mg 1-0-0 erfolgreich behandelt werden konnten. Der Patient wurde am 05.09.2038 entlassen.

EHR Data

ALLGEMEINE INFORMATIONEN
Geschlecht: männlich
Geburtsdatum: 13.12.1972
Bekannte Vorerkrankungen: Arterielle Hypertonie
Diabetes mellitus liegt vor seit 04/2037, Insulinpflicht seit 05/2037
Vorliegende Veränderungen: Kolonpolypen (Erstdiagnose: 04/2038)
Weitere Tumorerkrankungen (nicht am Pankreas):
-Darmkrebs: Therapie: Operation, Zeitpunkt: 05/2038
Vorhergehende abdominelle extrapankreatische OP liegt vor

INFORMATIONEN VOR OPERATION
Grund des Besuchs: Erstvorstellung; Akute Pankreatitis, Bauchspeicheldrüsenzyste
Datum der Erstdiagnose: 23.08.2038
Aktuelle Verdachtsdiagnose: Neuroendokriner Tumor (NET)
Diagnose histologisch nicht gesichert
Laborwerte: Carbohydrat-Antigen 19-9 (CA 19-9): 41.8 U/ml: Wert erhöht; Chromogranin A: 94.9 mikrogramm/L: Wert erhöht; Bilirubin gesamt: 1.06 mg/dl: Wert erhöht
Radiologischer Befund der Verdachtsdiagnose: Duktales Adenokarzinom des Pankreas (PDAC) / maligne Pankreastumoren, Resezierbarkeit des Tumors: Resektabel
Therapieempfehlung: Explorative Laparotomie, Pankreaslinksresektion mit Splenektomie, Cholezystektomie

INFORMATIONEN ZUR OPERATION
Datum der stationären Aufnahme: 26.08.2038
OP-Datum: 27.08.2038
Diagnose (gesichert): Neuroendokriner Tumor (NET)
Art der OP: Pankreaslinksresektion, OP-Zugangsmethode: Offene OP
Intraoperative Drainagen: Easy-Flow Drainage: Eingelegt am 27.08.2038, entfernt am 02.09.2038
Intraoperative Bewertung des Pankreasparenchyms/-gewebes: Textur: Hart, Pankreas nicht lipomatös
Sonstige Informationen aus der OP: Blutverlust von 300 ml, Antibiose nur intraoperativ verabreicht

STATIONÄRER VERLAUF
Verlegung nach OP in Aufwachraum am 27.08.2038, dann zeitnahe Verlegung auf Normalstation
Folgende Analgetika wurden verabreicht: Opioide p.o., Opioide i.v., Regionalanästhesie (inkl. Periduralanästhesie (PDA))
Es zeigten sich reizlose Wundverhältnisse

Mobilisation des Patienten war wie vor Operation möglich
Der Kostaufbau des Patienten erfolgte zeitgerecht
Suffiziente Darmpassage seit 31.08.2038
Sonstige Auffälligkeiten: Blutdruckspitzen von systolisch bis zu 200 mmHg. Anpassung der Antihypertensiva sowie das Neuansetzen von Bisoprolol 5 mg 1-0-0 durchgeführt. Hierunter zeigten sich die Blutdruckspitzen deutlich kupiert.
Entlassungsdatum: 05.09.2038

Summary 25

Generated Discharge Summary

Anamnese und Befund:
Der 62-jährige Patient wurde aufgrund einer Kontrolle ohne Bauchspeicheldrüsenoperation und einer Bauchspeicheldrüsenzyste in unsere Klinik aufgenommen. In der Vorgeschichte fand sich eine vorhergehende abdominelle extrapankreatische OP. Der Patient hat eine positive Familienanamnese für extrapankreatische Krebserkrankungen, insbesondere Lymphome bei seinen Eltern. Der Patient war ehemaliger Raucher und konsumiert Alkohol wöchentlich. Die Laborwerte zeigten erhöhte Werte für CEA, CA 19-9, Bilirubin, AP, GGT und Amylase. Die Verdachtsdiagnose lautete auf Stenose der biliodigestiven Anastomose und Pankreasanastomose. Der Patient hatte bereits eine Pankreaskopfresektion durchgeführt.

Therapie und Verlauf:
Der Patient wurde am 12.06.2054 einer explorativen Laparotomie, Adhäsiolyse, Pankreassegmentresektion mit Neuanlage einer Pankreatojejunostomie, Auflösung und Neuanlage der Hepaticojejunostomie und Gallengangsrevision, Umwandlung von single loop zu Roux-Y unterzogen. Intraoperativ wurde eine Blake Drainage angelegt, die zeitgerecht entfernt wurde. Der Blutverlust betrug 300 ml, und Antibiotika wurden nur intraoperativ verabreicht. Postoperativ wurde der Patient auf die Normalstation verlegt und erhielt Opioide p.o. und patientenkontrollierte Analgesie. Die Wundverhältnisse waren reizlos, und die Mobilisation des Patienten war wie vor der Operation möglich. Der Kostaufbau des Patienten erfolgte zeitgerecht, und eine suffiziente Darmpassage konnte ab dem 17.06.2054 festgestellt werden. Der Patient wurde am 18.06.2054 entlassen.

EHR Data

ALLGEMEINE INFORMATIONEN
Geschlecht: männlich
Geburtsdatum: 21.02.1992
Vorhergehende abdominelle extrapankreatische OP liegt vor
Familienanamnese: Extrapankreatische Krebserkrankungen liegen vor:
-Art der Erkrankung: Lymphom, Betroffene Familienmitglieder: Eltern, Alter Erkrankungsbeginn: 76
Raucherstatus: Ehemaliger Raucher: Aufgehört am: 01/2052, Anzahl Jahre: 15, Zigaretten pro Tag: 8
Alkoholkonsum: Häufigkeit: Wöchentlich

INFORMATIONEN VOR OPERATION
Grund des Besuchs: Kontrolle (ohne Bauchspeicheldrüsenoperation); Bauchspeicheldrüsenzyste
Verdachtsdiagnose: Stenose der biliodigestiven Anastomose und Pankreasanastomose
Histologische Sicherung nicht notwendig

Laborwerte: Carcinoembryonales Antigen (CEA): 4.2 mikrogramm/L: Wert erhöht; Carbohydrat-Antigen 19-9 (CA 19-9): 40.8 U/ml: Wert erhöht; Bilirubin gesamt: 2.43 mg/dl: Wert erhöht; Alkalische Phosphatase (AP): 395.0 U/L: Wert erhöht; Gamma-glutamyl Transferase (GGT): 1808.0 U/L: Wert erhöht; Amylase: 58.0 U/L: Wert erhöht
Vorhergehende Pankreas-OP: Pankreaskopfresektion, Zugang: minimalinvasiv, Datum der OP: 13.02.2052
Therapieempfehlung: Explorative Laparotomie, Neuanlage Hepaticojejunostomie, Neuanlage Pankreatikojejunostomie, Umwandlung nach Roux-Y am 27.11.2023

INFORMATIONEN ZUR OPERATION
Datum der stationären Aufnahme: 08.06.2054
OP-Datum: 12.06.2054
Diagnose (gesichert): Stenose der biliodigestiven Anastomose und Pankreasanastomose bei Z. n. daVinci-assistierter pyloruserhaltender Whipple'scher Operation am 28.07.2021 bei intraampullärer papillärer Neoplasie (IAPN) der Ampulla Vateri
Art der OP: Explorative Laparotomie, Adhäsiolyse, Pankreassegmentresektion mit Neuanlage einer Pankreatojejunostomie, Auflösung und Neuanlage der Hepaticojejunostomie und Gallengangsrevision, Umwandlung von single loop zu Roux-Y, OP-Zugangsmethode: Offene OP
Intraoperative Drainagen: Blake Drainage wurde zeitgerecht entfernt
Intraoperative Bewertung des Pankreasparenchyms/-gewebes: Textur: Hart, Position: Mittig, Durchmesser: > 3mm, Durchmesser in mm: 7, Pankreas nicht lipomatös
Sonstige Informationen aus der OP: Blutverlust von 300 ml, Antibiose nur intraoperativ verabreicht

STATIONÄRER VERLAUF
Verlegung nach OP in Aufwachraum am 12.06.2054, dann zeitnahe Verlegung auf Normalstation
Folgende Analgetika wurden verabreicht: Opioide p.o., patientenkontrollierte Analgesie
Es zeigten sich reizlose Wundverhältnisse
Mobilisation des Patienten war wie vor Operation möglich
Der Kostaufbau des Patienten erfolgte zeitgerecht
Suffiziente Darmpassage seit 17.06.2054
Entlassungsdatum: 18.06.2054
